# Estimating average causal effects with incomplete exposure and confounders

**DOI:** 10.1515/jci-2023-0083

**Published:** 2026-02-20

**Authors:** Lan Wen, Glen McGee

**Affiliations:** Department of Statistics and Actuarial Science, University of Waterloo, Waterloo, ON, Canada

**Keywords:** causal inference, missing not at random, multiple imputation, multiple robustness, outcome-independent missingness, targeted learning, 62D10, 62D20

## Abstract

Standard methods for estimating average causal effects require complete observations of the exposure and confounders. In observational studies, however, missing data are ubiquitous. Motivated by a study on the effect of prescription opioids on mortality, we propose methods for estimating average causal effects when exposures and potential confounders may be missing. We consider missingness at random and additionally propose several specific missing not at random (MNAR) assumptions. Under our proposed MNAR assumptions, we show that the average causal effects are identified from the observed data and derive corresponding influence functions, which form the basis of our proposed estimators. Our simulations show that standard multiple imputation techniques paired with a complete data estimator is unbiased when data are missing at random (MAR) but can be biased otherwise. For each of the MNAR assumptions, we instead propose doubly robust targeted maximum likelihood estimators (TMLE), allowing misspecification of either (i) the outcome models or (ii) the exposure and missingness models. The proposed methods are suitable for any outcome types, and we apply them to a motivating study that examines the effect of prescription opioid usage on all-cause mortality using data from the National Health and Nutrition Examination Survey (NHANES).

## 1 Introduction

Observational studies are often used to investigate causal questions in epidemiology. When outcomes, exposures and confounders are all completely observed, estimating causal effects proceeds via standard estimation techniques like parametric g-formula [1], [2], [3], propensity score-based methods [4], [5], [6], or doubly robust methods [7], [8], [9]. A limitation of observational studies, however, is that data are often missing. Consider, for example, a motivating analysis of the 1999–2004 cycle of the National Health and Nutrition Examination Survey (NHANES), which found that opioid prescriptions increased risk of all-cause mortality [10]. Our interest lies in estimating the average causal effect of opioid prescription on 5-year all-cause mortality among the elderly, and the data include several potential confounders. The NHANES data is linked to the National Death Index mortality data, so while the outcome of interest is fully observed, missing data arise in the exposure and potential confounders. More specifically, 24 % of observations were missing either the exposure of interest or a confounder in the analytic sample, so standard methods that rely on data being complete may not apply.

Extensions have been proposed to accommodate either missing exposures [11], [12], [13], [14] or missing confounders [11], [15], [16], [17], [18], [19], [20], [21], [22], [23], [24], [25], [26], [27], [28], [29], [30], [31]. When the exposure is missing at random (MAR; [32], [33], [34]) and confounders are fully observed, several authors proposed augmented inverse probability weighted estimators [11], [12], [13]. When confounders are MAR and exposure is fully observed, existing methods include multiple imputation (MI [15], [16], [17], [18]), influence function-based estimators [19], 31], 35] and inverse probability weighting (IPW [20]). When confounders are missing not at random (MNAR), further assumptions are needed. Under an outcome-independence assumption [22], proposed approaches include two-stage least squares estimation [23], imputation strategies [24], and doubly robust estimation [25] – though all rely on a rank constraint that precludes binary outcomes. Other authors assumed existence of a shadow variable [26] or that covariates act as confounders only when they are observed [21], 27], 28], 36].

Guidance is limited for estimating average causal effects when both exposures and confounders are partially observed. Several authors provided algorithms for identifiability and/or testability using graphical models [37], [38], [39], [40], some of which explicitly consider causal queries (see e.g., Mohan and Pearl [38], Section 6], which discusses the notion of trivial recoverability). Those graphical approaches derive conditional independence constraints via d-separation on missingness Directed Acyclic Graphs (DAGs) and provide general procedures for deciding recoverability of probabilistic and causal quantities. Building on DAGs depicting missingness mechanisms, Moreno-Betancur et al. [41] examined the identifiability of causal quantities but limited discussion of estimation to available-case analysis and MI.

Our work is complementary to this literature. Building on prior work, we retain Rubin’s classification of missing data mechanisms [32] and propose conditional independence assumptions that are sufficient for identification and expressed in terminology familiar from classical missing data literature. Under these assumptions we (i) give identification results targeted at average causal effects when both exposures and confounders are partially observed, (ii) derive explicit identifying functionals, and (iii) develop practical estimation strategies.

We begin with one of several assumptions about the missingness mechanism(s): besides a MAR assumption, we propose several variations of the outcome-independence assumption. Motivated by the NHANES analysis, the proposed assumptions permit missingness in the exposure – opioid prescription – to depend on missing potential confounders and the missing exposure itself, and allows shared unmeasured common causes between these aforementioned variables. Importantly, none of the assumptions restrict the types of outcomes allowed. Under each set of assumptions, we derive identifying formulae, and corresponding influence functions under a nonparametric model. We show that standard MI (e.g., Joint Multivariate Normal MI, Fully Conditional Specification MI) yields valid estimates when data are MAR but can be biased otherwise. For each of the proposed set of assumptions, we propose targeted maximum likelihood estimators (TMLE) that are doubly robust, allowing misspecification of either (i) the outcome models or (ii) the exposure and missingness models.

## 2 Setup: causal estimand and assumptions

Suppose we collect *n* fully observed, independent and identically distributed observations. Under this setting, let *Y* denote an outcome of interest, *A* an exposure variable taking values in N , and *L* a vector of potential confounders. For ease of exposition, we assume throughout that the outcome *Y* is binary and the covariates *L* are discrete, but methods described herein are generalizable to other outcomes types and to continuous covariates. Let *Y* ^*a*^ denote the potential outcome variable if, possibly contrary to fact, the exposure had taken a value *a* for *a* ∈ supp(*A*), and let *E*(*Y* ^*a*^) denote the corresponding *average* potential outcome of interest, had exposure taken a value *a* for all individuals in a population. To quantify average causal effects, we consider contrasts such as, *E*(*Y* ^*a* = 1^) − *E*(*Y* ^*a* = 0^). Throughout we will assume no interference or the stable unit-treatment value assumption (SUTVA; [42]), in addition to the following standard causal assumptions stated below:

**Assumption 1:**

(Consistency). If *A* = *a*, then *Y* ^*a*^ = *Y*,

**Assumption 2:**

(Conditional Exchangeability). *Y* ^*a*^ ⊥⊥ *A*∣*L*.

When the exposure or confounders are partially missing, we must make additional assumptions in order to identify *E*(*Y* ^*a*^), for any *a* ∈ supp(*A*). In this paper, we allow the exposure *A* and confounders *L* to be partially missing; in particular we let *L*_*M*_ represent the subvector of confounders that are partially missing, and *L*_*O*_ represent the subvector of confounders that are fully observed for all individuals; i.e., *L* = (*L*_*M*_, *L*_*O*_). In Sections 3 and 4, we consider various missingness mechanisms for *A* and *L*_*M*_. In Section 5, we show simulation results and illustrate theoretical findings empirically, and in Section 6, we apply the new methods to study the effect of opioid prescriptions on mortality using data from NHANES. We conclude with a discussion in Section 7.

## 3 Data that are missing at random

Due to its convenient implementation, standard off-the-shelf MI is frequently used in social and epidemiological studies to handle missing data in estimating average causal effects [10], [43], [44], [45], [46], [47], [48], [49]. This, however, implicitly requires that data are MAR. In this section, we briefly discuss identification and estimation under this assumption.

Separating the observation indicators for *L*_*M*_ and *A* would induce non-monotone missing data, for which an MAR mechanism is challenging to justify scientifically [50], [51], [52]. Hence, in this section, we treat *A* and *L*_*M*_ as either entirely missing or fully observed for each subject. To account for this, let *R* denote the observation status of (*L*_*M*_, *A*) such that *R* = 1 if both *L*_*M*_ and *A* are observed and *R* = 0 otherwise. As such, the observed data for an individual are *O* = (*L*_*O*_, *R*, *RL*_*M*_, *RA*, *Y*) ∼ *P*. We consider an (everywhere) MAR assumption [33] for both exposure and confounders as well as a positivity assumption for this missingness process:

**Assumption 3:**

(MAR). *R* ⊥⊥{*A*, *L*_*M*_}|*Y*, *L*_*O*_.

**Assumption 4:**

(Positivity: MAR). *P*(*R* = 1∣*L*_*O*_ = *l*, *Y* = *y*) > 0, *∀*(*l*_*O*_, *y*) ∈ supp(*L*_*O*_, *Y*).

Under assumptions 3 and 4, the causal quantity *E*(*Y* ^*a*^) is identified by the following:

(1) $$\begin{array}{ll} \Psi_{MAR}^{a} & =E\left[\frac{\sum_{y}yp\left(L_{M},a|L_{O},y,R=1\right)p\left(y|L_{O}\right)}{\sum_{y}p\left(L_{M},a|L_{O},y,R=1\right)p\left(y|L_{O}\right)}\right]\\ & =E\left[\frac{\beta \left(L\right)}{\gamma \left(L\right)}\frac{R}{P\left(R=1|L_{O},Y\right)}\right], \end{array}$$

where *β*(*L*) = *∑*_*y*_ *yp*(*L*_*M*_, *a*∣*L*_*O*_, *y*, *R* = 1)*p*(*y*∣*L*_*O*_) and *γ*(*L*) = *∑*_*y*_ *p*(*L*_*M*_, *a*∣*L*_*O*_, *y*, *R* = 1)*p*(*y*∣*L*_*O*_). This generalizes Levis et al. [31], who considered missing confounders only.

**Figure 1:**
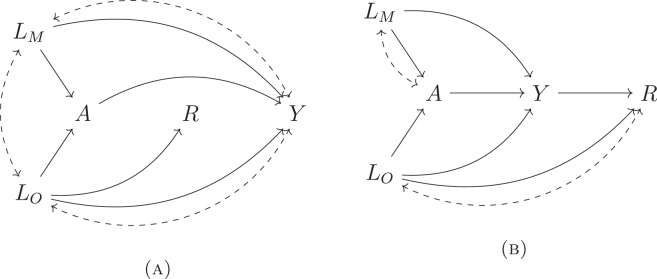
DAGs satisfying assumption 3 (MAR). Dashed lines represent potential unmeasured common causes.

MAR allows missingness status *R* to depend on observed confounders *L*_*O*_. In our data application, ‘income’ and ‘marital status’ are partially observed, and individuals’ reluctance to disclose these variables may vary with age, which is fully observed. We present two examples in Figure 1 in which Assumptions 2 and 3 are satisfied (note that these are non-exhaustive). In our DAG examples, the outcome may affect missingness in Figure 1[b] (e.g., in a retrospective study, one’s health outcome may affect how likely they are to stay in a study and report a past exposure status), but missingness may not affect the outcome in Figure 1[a]. The latter is a property that we assume throughout (see Srinivasan et al. [53] who relax this assumption). In general, the MAR assumption 3 can be restrictive, because neither the missing exposure *A* nor missing confounders *L*_*M*_ are allowed to affect *R*.

**Remark 1.**

Note that in Figure 1[a], the conditional independence implied by Assumption 3 holds under a different condition – conditioning on *L*_*O*_ alone, so there can be multiple sufficient sets of assumptions for that structure. We use this DAG purely as an illustrative example of a structure that satisfies our MAR assumption.

In Web Appendix E, we derive an influence function for $\Psi_{MAR}^{a}$ given by identifying formula (1) under a nonparametric model for *P*. In contrast to the other identifying formulae we present, the form of the influence function requires specifying the joint distribution of *L*_*M*_ and *A*, hence using this as a basis for estimation is practically challenging. Alternatively, we can leverage the MAR assumption and exploit standard MI with available software – a common practice in social and epidemiological studies – then apply existing TMLE approach to the imputed complete data ([54]; see Web Appendix F for more details). While MI is easy to implement, the MAR assumption can be overly restrictive, as described above. In the next section, we propose assumptions that deviate from MAR and hence correspond to MNAR in the missing data literature [55].

## 4 Data that are not missing at random

We now consider separating the observation indicators of exposure *A* and confounders *L*_*M*_. In the following, we require that the missingness mechanism be *outcome-independent*: *R* ⊥⊥ *Y*|*A*, *L* [23], 26], 56], but do not assume any rank conditions. The outcome-independence assumption may be reasonable e.g., in prospective studies where covariates and exposure are measured before the outcome occurs [23], but may be less plausible if the reasons for missingness (e.g., poor underlying health conditions) also affect the outcome of interest.

In what follows, let *R*_*A*_ denote the observation indicator for exposure (*R*_*A*_ = 1 when *A* is observed and *R*_*A*_ = 0 otherwise). In addition, we posit two classes of missing data mechanisms for the partially observed covariates *L*_*M*_ that fall outside the full-data MAR framework. These two missingness mechanisms impose additional, stronger assumptions for the missingness in *L*_*M*_ than those imposed for the missingness in the exposure.

### 4.1 Identification and estimation under an MNAR mechanism

Let *R*_*L*_ denote the observation indicator for covariates *L*_*M*_ (*R*_*L*_ = 1 when *L*_*M*_ is entirely observed and *R*_*L*_ = 0 otherwise). Here, *R*_*A*_ may differ from *R*_*L*_, and we now observe *O* = (*L*_*O*_, *R*_*A*_, *R*_*L*_, *R*_*L*_ *L*_*M*_, *R*_*A*_ *A*, *Y*) ∼ *P*. Consider the following set of missingness assumptions $\left(I_{A}\right)$ :

**Assumption 5:**

( $I_{A}$ : Outcome-independence with MAR relative to *L*).

(1) *R*_*A*_ *-outcome independence condition*: *R*_*A*_ ⊥⊥ *Y*|*A*, *L*
(2) *R*_*L*_ *-outcome independence condition*: *R*_*L*_ ⊥⊥ *Y*|*A*, *L*, *R*_*A*_, and
(3) *MAR for* *L*_*M*_ *(relative to* *L* *)*: *R*_*L*_ ⊥⊥ *L*_*M*_|*L*_*O*_.

**Remark 2.**

In assumption 5.(3), *R*_*L*_ can share unmeasured common causes with *A*, and thus the mechanism is not MAR relative to the full data in the traditional sense.

**Assumption 6:**

(Positivity: $I_{A}$ ).

$$P\left(A=a|R=1,L=l\right)>0,\;\;\forall l\in \text{supp}\left(L\right),\;\text{and}\;P\left(R_{L}=1|L_{O}=l_{O}\right)>0,\;\;\forall l_{O}\in \text{supp}\left(L_{O}\right).$$

Under assumptions 5 and 6, *E*(*Y* ^*a*^) can be identified using the following functional:

(2) $$\Psi_{I_{A}}^{a}=\underset{l}{\sum }E\left(Y|A=a,R_{A}=1,R_{L}=1,L\right)p\left(l_{M}|l_{O},R_{L}=1\right)p\left(l_{O}\right).$$

**Figure 2:**
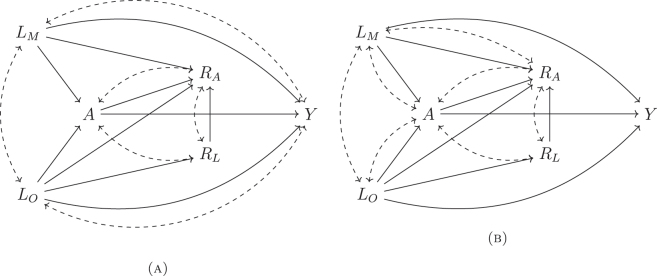
Example DAG satisfying $I_{A}$ assumption 5. Dashed lines represent potential unmeasured common causes.

Figure 2 shows two DAGs satisfying these assumptions, where *R*_*A*_ can be affected by *A* and *L*_*M*_, and both *R*_*A*_ and *R*_*L*_ can share common causes with *A*. This might be the case in observational studies if exposure information is sensitive, such as opioid intake. For instance, it is possible that opioid usage affects one’s perceived social status, which in turn can affect whether someone reports their opioid usage. It is also possible that an individual’s perception of certain social factors (e.g., societal and cultural norms) can affect whether they take opioids (exposure) and whether they report their exposure and covariate statuses.

#### 4.1.1 Estimation

Our work builds on recent advances in influence-function-based estimation under missing data (see Kennedy [13] for missingness in exposure; Levis et al. [31] for missingness in covariates; Bia et al. [57], de Aguas et al. [58] for missingness in outcomes). We first derive the corresponding influence function for the functional $\Psi_{I_{A}}^{a}$ given by identifying formula (2) under a nonparametric model for *P*. The resulting influence function is given by:

(3) $$\phi_{P_{I_{A}}}^{1}\left(O\right)=\frac{I\left(A=a,R_{A}=1,R_{L}=1\right)}{\pi_{A}\left(L\right)\pi_{R_{A}}\left(L\right)\pi_{R_{L}}\left(L_{O}\right)}\left\{Y-T_{1}\left(L\right)\right\}+\frac{I\left(R_{L}=1\right)}{\pi_{R_{L}}\left(L_{O}\right)}\left\{T_{1}\left(L\right)-T_{0}\left(L_{O}\right)\right\}+T_{0}\left(L_{O}\right)-\Psi_{I_{A}}^{a}$$

where *π*_*A*_(*L*) = *P*(*A* = *a*∣*R* = 1, *L*), $\pi_{R_{A}}\left(L\right)=P\left(R_{A}=1|L,R_{L}=1\right)$ , $\pi_{R_{L}}\left(L_{O}\right)=P\left(R_{L}=1|L_{O}\right)$ , *T*_1_(*L*) = *E*(*Y*∣*A* = *a*, *R* = 1, *L*), and *T*_0_(*L*_*O*_) = *E*(*T*_1_(*L*)∣*L*_*O*_, *R*_*L*_ = 1). With this influence function in hand, we can construct consistent estimators of $\Psi_{I_{A}}^{a}$ , such as the solution to estimating equations or the TMLE. We propose a TMLE via Algorithm in Table 1, which reduces to the complete-data TMLE when there is no missing data [54].

**Table 1:** Algorithm for TMLE-A.

Algorithm 1 TMLE-A under $I_{A}$
1: Obtain estimates ${\hat{\pi }}_{A}\left(L\right)$ , ${\hat{\pi }}_{R_{A}}\left(L\right)$ and ${\hat{\pi }}_{R_{L}}\left(L_{O}\right)$ of *π*_*A*_(*L*), $\pi_{R_{A}}\left(L\right)$ and $\pi_{R_{L}}\left(L_{O}\right)$ , respectively.
2: *Obtain initial prediction of* *T*_1_(*L*)*,* ${\hat{T}}_{1}^{0}\left(L\right)$ : Among those with *R* = 1, fit a regression model $\eta_{1}\left(A,L;\kappa_{1}\right)=g^{-1}\left({\left[A,L\right]}^{\prime }\kappa_{1}\right)$ by regressing *Y* on *A* and *L, where *g^−1^. *denotes a known inverse link function*. Obtain predictions ${\hat{T}}_{1}^{0}\left(L\right)=\eta_{1}\left(A,L;{\hat{\kappa }}_{1}\right)$ for these individuals.
3: *Targeting step for* *T*_1_(*L*) *to obtain updated prediction* ${\hat{T}}_{1}^{*}\left(L\right)$ : Among those with (*A*, *R*) = (*a*, 1), regress *Y* on an intercept with observational weight ${\left\{{\hat{\pi }}_{A}\left(L\right){\hat{\pi }}_{R_{A}}\left(L\right){\hat{\pi }}_{R_{L}}\left(L_{O}\right)\right\}}^{-1}$ and an offset given by $g\left\{{\hat{T}}_{1}^{0}\left(L\right)\right\}$ , i.e., solve for *ϵ*_1_ in $$P_{n}\left\{\frac{I\left(A=a,R_{A}=1,R_{L}=1\right)}{{\hat{\pi }}_{A}\left(L\right){\hat{\pi }}_{R_{A}}\left(L\right){\hat{\pi }}_{R_{L}}\left(L_{O}\right)}\left(Y-g^{-1}\left[g\left\{{\hat{T}}_{1}^{0}\left(L\right)\right\}+\epsilon_{1}\right]\right)\right\}=0$$ Among those with *R*_*L*_ = 1, predict *T*_1_(*L*) using ${\hat{T}}_{1}^{*}\left(L\right)=g^{-1}\left[g\left\{{\hat{T}}_{1}^{0}\left(L\right)\right\}+{\hat{\epsilon }}_{1}\right]$ .
4: *Obtain initial prediction of* *T*_0_(*L*_*O*_)*,* ${\hat{T}}_{0}^{0}\left(L_{O}\right)$ : Among those with *R*_*L*_ = 1, fit a regression model $\eta_{0}\left(L_{O};\kappa_{0}\right)=g^{-1}\left(L_{O}^{\prime }\kappa_{0}\right)$ by regressing ${\hat{T}}_{1}^{*}\left(L\right)$ on *L*_*O*_. Obtain predictions ${\hat{T}}_{0}^{0}\left(L_{O}\right)=\eta_{0}\left(L_{O};{\hat{\kappa }}_{0}\right)$ for these individuals.
5: *Targeting step for* *T*_0_(*L*_*O*_) to obtain updated prediction ${\hat{T}}_{0}^{*}\left(L_{O}\right)$ : Among those with *R*_*L*_ = 1, regress ${\hat{T}}_{1}^{*}\left(L\right)$ on an intercept with observational weight ${\hat{\pi }}_{R_{L}}{\left(L_{O}\right)}^{-1}$ and an offset given by $g\left\{{\hat{T}}_{0}^{0}\left(L_{O}\right)\right\}$ , i.e., solve for *ϵ*_0_ in $$P_{n}\left\{\frac{I\left(R_{L}=1\right)}{{\hat{\pi }}_{R_{L}}\left(L_{O}\right)}\left({\hat{T}}_{1}^{*}\left(L\right)-g^{-1}\left[g\left\{{\hat{T}}_{0}^{0}\left(L_{O}\right)\right\}+\epsilon_{0}\right]\right)\right\}=0$$ Predict *T*_0_(*L*_*O*_) using ${\hat{T}}_{0}^{*}\left(L_{O}\right)=g^{-1}\left[g\left\{{\hat{T}}_{0}^{0}\left(L_{O}\right)\right\}+{\hat{\epsilon }}_{0}\right]$ for all observations.
6: Calculate the TMLE-A estimator ${\hat{\Psi }}_{TMLE,I_{A}}^{a}=P_{n}\left\{{\hat{T}}_{0}^{*}\left(L_{O}\right)\right\}$ .

**Proposition 1.**

TMLE-A is multiply robust: it is consistent for $\Psi_{I_{A}}^{a}$ if (i) *T*_1_(*L*) and *T*_0_(*L*_*O*_) are correctly specified; (ii) *π*_*A*_(*L*), $\pi_{R_{A}}\left(L\right)$ , and $\pi_{R_{L}}\left(L\right)$ are correctly specified; or (iii) *T*_1_(*L*) and $\pi_{R_{L}}\left(L\right)$ are correctly specified. When all nuisance functions are correctly specified, it attains the nonparametric efficiency bound for the functional $\Psi_{I_{A}}^{a}$ .

We also describe the asymptotic properties of TMLE-A when the nuisance functions are estimated with flexible machine learning algorithms, and consider the challenges in the Discussion. This can be accomplished by estimating the nuisance functions via, e.g., generalized additive models, adaptive regression splines or other flexible machine learning algorithms.

**Theorem 1:**

(Weak convergence of TMLE-A). Suppose that the conditions given in Web Appendix H hold, and further suppose that the following condition also holds:

$$\begin{array}{ll} & {\left\|{\hat{T}}_{1}\left(L\right)-T_{1}\left(L\right)\right\|}_{2}\;{\left\|{\hat{\pi }}_{A}\left(L\right){\hat{\pi }}_{R_{A}}\left(L\right)-\pi_{A}\left(L\right)\pi_{R_{A}}\left(L\right)\right\|}_{2}\\ & +{\left\|{\hat{T}}_{0}\left(L_{O}\right)-T_{0}\left(L_{O}\right)\right\|}_{2}\;{\left\|{\hat{\pi }}_{R_{L}}\left(L_{O}\right)-\pi_{R_{L}}\left(L_{O}\right)\right\|}_{2}=o_{p}\left(n^{-1/2}\right). \end{array}$$

where ${\left\|f\left(x\right)\right\|}_{2}={\left\{\int |f\left(x\right){|}^{2}dP\left(x\right)\right\}}^{1/2}$ , i.e. the *L*_2_(*P*) norm. Then, $\sqrt{n}\left({\hat{\Psi }}_{TMLE,I_{A}}^{a}-\Psi_{I_{A}}^{a}\right)⇝N\left(0,\sigma^{2}\right),\;\;\sigma^{2}=\mathrm{Var}\left(\phi_{P_{I_{A}}}^{1}\right)$ .

As such, when the nuisance functions are estimated with machine learning algorithms, the variance of TMLE-A can be estimated empirically with $P_{n}\left[{\left({\hat{\phi }}_{P_{I_{A}}}^{1}\right)}^{2}\right]$ , where all nuisance functions are replaced with their estimates. When the nuisance functions are estimated with parametric models, this variance estimator remains applicable provided that all nuisance functions are correctly specified. However, in practice, we recommend using the nonparametric bootstrap to estimate the variance, as we are more susceptible to model misspecification with parametric models. By treating missingness in *A* and *L*_*M*_ separately, we are able to consider a more flexible set of assumptions. In particular, this set of assumptions allows *R*_*A*_ to depend on *A* and *L*_*M*_. However, it is still quite restrictive with respect to confounder missingness, as *R*_*L*_ still cannot depend on any variables in *L*_*M*_. Again, we can make progress by taking a more granular approach; in the following section, we consider distinct missingness mechanisms for different confounders.

### 4.2 A strategy for separating observation indicators in *L*_*M*_

Suppose that there are *q* variables in *L*_*M*_ such that *L*_*M*_ = (*L*_*M*1_, …, *L*_*Mq*_). Let *R*_*Lk*_ denote the observation indicator for covariate *L*_*Mk*_ for *k* = 1, …, *q* (i.e., *R*_*Lk*_ = 1 if *L*_*Mk*_ is observed and *R*_*Lk*_ = 0 otherwise). Furthermore, for any random variable *X* of length *q*, let ${\overset{®}{X}}_{k}=\left(X_{1},\ldots ,X_{k}\right)$ , $X:={\overset{®}{X}}_{q}=\left(X_{1},\ldots ,X_{q}\right)$ and ${\underline{X}}_{k}=\left(X_{k},\ldots ,X_{q}\right)$ . We now observe *O* = (*L*_*O*_, *R*_*L*1_, …, *R*_*Lq*_, *R*_*A*_, *R*_*L*1_ *L*_*M*1_, …, *R*_*Lq*_ *L*_*Mq*_, *R*_*A*_ *A*, *Y*) ∼ *P* and consider the following assumptions $\left(I_{B}\right)$ for identification in this setting:

**Assumption 7:**

( $I_{B}$ : Outcome-independence with block-conditional MAR relative to *L*).

(1) *R*_*A*_ *-outcome independence condition:* *R*_*A*_ ⊥⊥ *Y*|*A*, *L*
(2) *R*_*L*_ *-outcome independence condition*: (*R*_*L*1_, …, *R*_*Lq*_) ⊥⊥ *Y*|*A*, *L*, *R*_*A*_, and
(3) *Block-conditional MAR for* *L*_*Mk*_ *(relative to L)*: $R_{Lk}⊥\;\;\;\;\;\;\;⊥{\underline{L}}_{Mk}|{\overset{®}{R}}_{L,k-1},{\overset{®}{L}}_{M,k-1},L_{O}$ , *∀k*.

**Remark 3.**

Assumption 7.(3) follows the block-conditional MAR framework of Zhou et al. [59]; as they emphasize, “*this mechanism is not in general MAR*”, but rather a form of MNAR model. In our setting, *R*_*Lk*_ can depend on ${\overset{®}{L}}_{M,k-1}$ which are partially observed (similarly argued in [59]); thus MAR generally does not hold.

**Assumption 8:**

(Positivity: $I_{B}$ ).

$$P\left(A=a|R=1,L\right)>0,\;\;\text{and}\;\;P\left(R_{Lk}=1|{\overset{®}{R}}_{L,k-1}=1_{k-1},{\overset{®}{L}}_{M,k-1},L_{O}\right)>0,\;\;\forall k=1,\ldots ,q$$

where 1_*k*_ denotes a vector of ones of length *k*, for any *k*.

**Figure 3:**
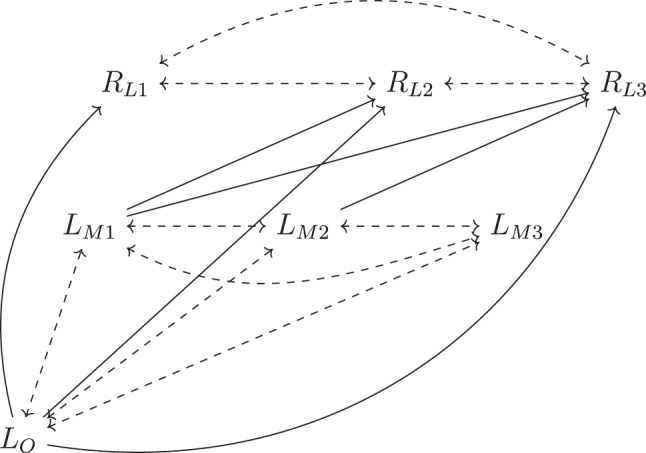
Examples of a DAG satisfying missingness assumption $R_{Lk}⊥\;\;\;\;\;\;\;⊥{\underline{L}}_{Mj}|{\overset{®}{R}}_{j-1},{\overset{®}{L}}_{M,j-1},L_{O}$ , for *q* = 3. Dashed lines represent potential unmeasured common causes.

Note that $R_{Lk}⊥\;\;\;\;\;\;\;⊥{\underline{L}}_{Mk}|{\overset{®}{R}}_{L,k-1},{\overset{®}{L}}_{M,k-1},L_{O}$ is equivalent to ${\overset{®}{R}}_{Lk}⊥\;\;\;\;\;\;\;⊥{\underline{L}}_{Mk}|{\overset{®}{L}}_{M,k-1},L_{O}$ . Figure 3 illustrates a scenario satisfying $R_{Lk}⊥\;\;\;\;\;\;\;⊥{\underline{L}}_{Mk}|{\overset{®}{R}}_{L,k-1},{\overset{®}{L}}_{M,k-1},L_{O}$ for *q* = 3. Neither assumption 5 nor 7 imply one another. Specifically, it can be shown that in general, $\prod_{k=1}^{q}R_{Lk}⊥\;\;\;\;\;\;\;⊥L_{M}|L_{O}$ does not imply $R_{Lk}⊥\;\;\;\;\;\;\;⊥{\underline{L}}_{Mk}|{\overset{®}{R}}_{L,k-1},{\overset{®}{L}}_{M,k-1},L_{O},\;\;\forall k=1,\ldots ,q$ , nor vice versa.

Under assumptions 7 and 8, *E*(*Y* ^*a*^) can be identified using the following functional:

(4) $$\Psi_{I_{B}}^{a}=\underset{l}{\sum }E\left(Y|A=a,R_{A}=1,{\overset{®}{R}}_{Lq}=1,L\right)\overset{q}{\underset{k=1}{\prod }}p\left(l_{Mk}|l_{O},{\overset{®}{l}}_{M,k-1},{\overset{®}{R}}_{Lk}=1_{k}\right)p\left(l_{O}\right).$$

where ${\overset{®}{L}}_{M0}=\emptyset$ by definition.

The ordering of the confounders plays an important role here. First, the assumptions for *R*_*Lj*_ and *R*_*Lk*_ differ for *j* < *k*. In particular, one could order the confounders in *L*_*M*_ to best capture the underlying causal structure where, e.g., *L*_*Mj*_ may potentially affect *R*_*Lk*_ but *L*_*Mk*_ does not affect *R*_*Lj*_. In our data application, education was subject to missingness and was thought to potentially affect the missingness status of other covariates like income. Hence, including education as the first covariate in *L*_*M*_ (i.e., as *L*_*M*1_) allows missingness status of income to depend on education. Second, even if data are not monotone missing (monotone in the sense that if *L*_*Mk*_ is observed, {*L*_*M*1_, …, *L*_*M*,*k*−1_} must be observed, *∀k*), monotone missingness will be implicitly enforced in estimators that are based on identifying formula (4). That is, *L*_*Mk*_ will be treated as missing if any of {*L*_*M*1_, …, *L*_*M*,*k*−1_} are missing. Hence, in our example, income will be treated as though it were missing whenever education is missing. In the following, we will see how this artificial monotonicity can affect estimation.

#### 4.2.1 Estimation

We consider estimation of the functional $\Psi_{I_{B}}^{a}$ given by identifying formula (4) using a TMLE motivated by the corresponding influence function for $\Psi_{I_{B}}^{a}$ under a nonparametric model for *P*:

(5) $$\begin{array}{ll} \phi_{P_{I_{B}}}^{1} & =\frac{I\left(A=a,R_{A}=1,{\overset{®}{R}}_{Lq}=1_{q}\right)}{\pi_{A}\left(L\right)\pi_{R_{A}}\left(L\right)\prod_{k=1}^{q}\pi_{R_{Lk}}\left({\overset{®}{L}}_{M,k-1},L_{O}\right)}\left\{Y-{\tilde{T}}_{q}\left(L\right)\right\}\\ & \;+\overset{q}{\underset{k=1}{\sum }}\frac{I\left({\overset{®}{R}}_{Lk}=1_{k}\right)}{\prod_{j=1}^{k}\pi_{R_{Lj}}\left({\overset{®}{L}}_{M,j-1},L_{O}\right)}\left\{{\tilde{T}}_{k}\left({\overset{®}{L}}_{Mk},L_{O}\right)-{\tilde{T}}_{k-1}\left({\overset{®}{L}}_{M,k-1},L_{O}\right)\right\}+{\tilde{T}}_{0}\left(L_{O}\right)-\Psi_{I_{B}}^{a} \end{array}$$

where the probability of observing each covariate *k* in *L*_*M*_ is given by:

$$\pi_{R_{Lk}}\left({\overset{®}{L}}_{M,k-1},L_{O}\right)=P\left(R_{Lk}=1|{\overset{®}{R}}_{L,k-1}=1_{k-1},{\overset{®}{L}}_{M,k-1},L_{O}\right),$$

for *k* = 1, …, *q*, and the new outcomes are given by:

$${\tilde{T}}_{k}\left({\overset{®}{L}}_{Mk},L_{O}\right)=E\left\{{\tilde{T}}_{k+1}\left({\overset{®}{L}}_{M,k+1},L_{O}\right)|{\overset{®}{L}}_{Mk},L_{O},{\overset{®}{R}}_{Lk}=1_{k}\right\}$$

defined iteratively backwards from *k* = *q* − 1 to *k* = 0, with ${\tilde{T}}_{q}\left(L\right)=T_{1}\left(L\right)$ . In Table 2, we present a TMLE motivated by the influence function given by (5).

**Table 2:** Algorithm for TMLE-B.

Algorithm 2 TMLE-B under $I_{B}$
1: Obtain estimates ${\hat{\pi }}_{A}\left(L\right)$ and ${\hat{\pi }}_{R_{A}}\left(L\right)$ and ${\hat{\pi }}_{R_{Lk}}\left({\overset{®}{L}}_{M,k-1},L_{O}\right)$ of *π*_*A*_(*L*), $\pi_{R_{A}}\left(L\right)$ and $\pi_{R_{Lk}}\left({\overset{®}{L}}_{M,k-1},L_{O}\right)$ (for *k* = 1, …, *q*), respectively.
2: *Obtain initial prediction of* ${\tilde{T}}_{q}\left(L\right)$ *,* ${\hat{\tilde{T}}}_{q}^{0}\left(L\right)$ : Among those with *R*_*A*_ = 1 and ${\overset{®}{R}}_{Lq}=1_{q}$ , fit a regression model *ζ*(*A*, *L*; *κ*) = *g* ^−1^([*A*, *L*]′*κ*) by regressing *Y* on *A* and *L*. Obtain predictions ${\hat{\tilde{T}}}_{q}^{0}\left(L\right)=\zeta \left(A,L;\hat{\kappa }\right)$ for these individuals.
3: *Targeting step for* ${\tilde{T}}_{q}\left(L\right)$ *to obtain updated predictions* ${\hat{\tilde{T}}}_{q}^{*}\left(L\right)$ *:* Among those with *A* = *a*, *R*_*A*_ = 1 and ${\overset{®}{R}}_{Lq}=1_{q}$ , regress *Y* on an intercept with observational weight ${\left\{{\hat{\pi }}_{A}\left(L\right){\hat{\pi }}_{R_{A}}\left(L\right)\prod_{k=1}^{q}{\hat{\pi }}_{R_{Lk}}\left({\overset{®}{L}}_{M,k-1},L_{O}\right)\right\}}^{-1}$ and offset given by $g\left\{{\hat{\tilde{T}}}_{q}^{0}\left(L\right)\right\}$ , i.e., solve for *ϵ* in $$P_{n}\left\{\frac{I\left(A=a,R_{A}=1,{\overset{®}{R}}_{Lq}=1_{q}\right)}{{\hat{\pi }}_{A}\left(L\right){\hat{\pi }}_{R_{A}}\left(L\right)\prod_{k=1}^{q}{\hat{\pi }}_{R_{L}}\left({\overset{®}{L}}_{M,k-1},L_{O}\right)}\left(Y-g^{-1}\left[g\left\{{\hat{\tilde{T}}}_{q}^{0}\left(L\right)\right\}+\epsilon \right]\right)\right\}=0$$ Among those with ${\overset{®}{R}}_{Lq}=1_{q}$ , predict ${\tilde{T}}_{q}\left(L\right)$ using ${\hat{\tilde{T}}}_{q}^{*}\left(L\right)=g^{-1}\left[g\left\{{\hat{\tilde{T}}}_{q}^{0}\left(L\right)\right\}+\hat{\epsilon }\right]$ .
4: Recursively from *k* = *q*, …, 1: (A) *Obtain initial prediction of* ${\tilde{T}}_{k-1}\left({\overset{®}{L}}_{M,k-1},L_{O}\right)$ *,* ${\hat{\tilde{T}}}_{k-1}^{0}\left({\overset{®}{L}}_{M,k-1},L_{O}\right)$ : Among those with ${\overset{®}{R}}_{Lk}=1_{k}$ , fit a regression model $\eta_{k-1}\left({\overset{®}{L}}_{M,k-1},L_{O};\omega_{k-1}\right)=g^{-1}\left({\left[{\overset{®}{L}}_{M,k-1},L_{O}\right]}^{\prime }\omega_{k-1}\right)$ by regressing ${\hat{\tilde{T}}}_{k}\left({\overset{®}{L}}_{Mk},L_{O}\right)$ on ${\overset{®}{L}}_{M,k-1}$ and *L*_*O*_. Obtain predictions ${\hat{\tilde{T}}}_{k-1}^{0}\left(L\right)=\eta_{k-1}\left({\overset{®}{L}}_{M,k-1},L_{O};{\hat{\omega }}_{k-1}\right)$ for these individuals. (B) *Targeting step for* ${\tilde{T}}_{k-1}\left({\overset{®}{L}}_{M,k-1},L_{O}\right)$ *to obtain updated predictions* ${\hat{\tilde{T}}}_{k-1}^{*}\left({\overset{®}{L}}_{M,k-1},L_{O}\right)$ *:* Among those with ${\overset{®}{R}}_{Lk}=1_{k}$ , regress ${\hat{\tilde{T}}}_{k}^{*}\left({\overset{®}{L}}_{Mk},L_{O}\right)$ on an intercept with observational weight $\prod_{j=1}^{k}{\hat{\pi }}_{R_{Lj}}{\left({\overset{®}{L}}_{M,j-1},L_{O}\right)}^{-1}$ and an offset term given by $g\left\{{\hat{\tilde{T}}}_{k-1}^{0}\left({\overset{®}{L}}_{M,k-1},L_{O}\right)\right\}$ , i.e., solve for *ν*_*k*−1_ in $$P_{n}\left\{\frac{I\left({\overset{®}{R}}_{Lk}=1_{k}\right)}{\prod_{j=1}^{k}{\hat{\pi }}_{R_{Lj}}\left({\overset{®}{L}}_{M,j-1},L_{O}\right)}\left({\hat{\tilde{T}}}_{k}^{*}\left({\overset{®}{L}}_{Mk},L_{O}\right)-g^{-1}\left[g\left\{{\hat{\tilde{T}}}_{k-1}^{0}\left({\overset{®}{L}}_{M,k-1},L_{O}\right)\right\}+\nu_{k-1}\right]\right)\right\}=0$$ Among those with ${\overset{®}{R}}_{L,k-1}=1_{k-1}$ , predict ${\tilde{T}}_{k-1}\left({\overset{®}{L}}_{M,k-1},L_{O}\right)$ using ${\hat{\tilde{T}}}_{k-1}^{*}\left({\overset{®}{L}}_{M,k-1},L_{O}\right)=g^{-1}\left[g\left\{{\hat{\tilde{T}}}_{k-1}^{0}\left({\overset{®}{L}}_{M,k-1},L_{O}\right)\right\}+{\hat{\nu }}_{k-1}\right]$ .
5: Calculate the TMLE estimator ${\hat{\Psi }}_{TMLE,I_{B}}=P_{n}\{{\hat{\tilde{T}}}_{0}^{*}\left(L_{O}\right)\}$ .

TMLE-B requires one to specify more models than the estimators proposed earlier, as now we are separately considering the missingness in each incompletely observed covariate. For instance, if we have *q* incompletely observed covariates, then we will need to specify *q* − 1 more missingness models as well as *q* − 1 more outcome models compared with TMLE-A.

**Proposition 2.**

TMLE-B is multiply robust: it is consistent for $\Psi_{I_{B}}^{a}$ if (i) the models for *π*_*A*_(*L*), $P\left(R_{A}=1|L,{\overset{®}{R}}_{Lq}=1_{q}\right)$ and $P\left(R_{Lk}=1|{\overset{®}{L}}_{M,k-1},R_{L,k-1}=1_{k},L_{O}\right),\;\forall k=1,\ldots ,q$ are correctly specified; (ii) the models for ${\tilde{T}}_{k}\left({\overset{®}{L}}_{Mk},L_{O}\right),\;\forall k=0,\ldots ,q$ are correctly specified; or (iii) for each *j* = 1, …, *q*, $P\left(R_{Lh}=1|{\overset{®}{L}}_{M,h-1},R_{L,h-1}=1_{h},L_{O}\right)$ are correctly specified for *h* = 1, …, *j* and ${\tilde{T}}_{k}\left({\overset{®}{L}}_{Mk},L_{O}\right)$ are correctly specified for *k* = *j*, …, *q*.

The asymptotic properties of TMLE-B when the nuisance functions are estimated with machine learning algorithms are given as follows:

**Theorem 2:**

(Weak convergence of TMLE-B). Suppose that the conditions given in Web Appendix H hold, and further suppose that the following condition also holds:

$$\begin{array}{ll} & \overset{q}{\underset{k=1}{\sum }}{\left\|{\hat{\tilde{T}}}_{k-1}\left(L_{O},{\overset{®}{L}}_{M,k-1}\right)-{\tilde{T}}_{k-1}\left(L_{O},{\overset{®}{L}}_{M,k-1}\right)\right\|}_{2}{\left\|{\hat{\pi }}_{R_{Lk}}\left(L_{O},{\overset{®}{L}}_{M,k-1}\right)-\pi_{R_{Lk}}\left(L_{O},{\overset{®}{L}}_{M,k-1}\right)\right\|}_{2}\\ & +{\left\|{\hat{\tilde{T}}}_{q}\left(L_{O},{\overset{®}{L}}_{Mq}\right)-{\tilde{T}}_{q}\left(L_{O},{\overset{®}{L}}_{Mq}\right)\right\|}_{2}\;{\left\|{\hat{\pi }}_{A}\left(L\right){\hat{\pi }}_{R_{A}}\left(L\right)-\pi_{A}\left(L\right)\pi_{R_{A}}\left(L\right)\right\|}_{2}=o_{p}\left(n^{-1/2}\right). \end{array}$$

Then,

$$\sqrt{n}\left({\hat{\Psi }}_{TMLE,I_{B}}^{a}-\Psi_{I_{B}}^{a}\right)⇝N\left(0,\sigma^{2}\right),\;\;\text{where}\;\sigma^{2}=\mathrm{Var}\left(\phi_{P_{I_{B}}}^{1}\right).$$

The variance of TMLE-B can be estimated as the empirical variance of ${\hat{\phi }}_{P_{I_{B}}}^{1}$ (where all nuisance functions are replaced with their estimates) or via nonparametric bootstrap.

**Remark 4.**

Since $\Psi_{I_{A}}^{a}$ and $\Psi_{I_{B}}^{a}$ are not functions of the exposure or missingness distributions, these TMLEs will attain the efficiency bound under a submodel of the nonparametric model where the observed propensity score and missingness models are known or modelled parametrically [60]. As such, estimators based on our influence function under correctly specified nuisance functions will be at least as efficient as IPW estimators.

Clearly, different orderings of variables in *L*_*M*_ correspond to different influence functions for $\Psi_{I_{B}}^{a}$ identified by (4). We have already seen that if the *j*th covariate in *L*_*M*_ potentially affect the observation status of the *k*th covariate in *L*_*M*_, then it must be that *j* < *k* in order to satisfy assumption 7. However, if there exist a subset of covariates in *L*_*M*_ that do not affect the observation status of any covariate in *L*_*M*_, then there may be different valid orderings, all of which satisfy assumption 7. In situations such as these, the ordering of *L*_*M*_ may have important implications for efficiency. Without loss of generality, suppose that variables in *L*_*M*_ include ‘education’, ‘income’ and ‘marital status’ (i.e., *q* = 3). We let *L*_*M*1_ denote ‘education’ as it can potentially affect the observation status of ‘income’ and ‘marital status’, but we hypothesize that ‘income’ and ‘marital status’ do not affect the observation status of any covariate in *L*_*M*_. In this case, the ordering of ‘income’ and ‘marital status’ in *L*_*M*_ is arbitrary, and any ordering will satisfy $\left(L_{M2},L_{M3}\right)⊥\;\;\;\;\;\;\;\;⊥{\overset{®}{R}}_{L2}|L_{O},L_{M1}$ . However, to make the most use of the available data and potentially optimize efficiency, we may want to place ‘income’ and ‘marital status’ in the order of increasing amount of missingness.

## 5 Simulations

We conducted a series of simulations to demonstrate that: (1) MI followed by application of the complete-data TMLE yields valid estimates of the average potential outcome under the MAR assumption, but may yield biased estimates otherwise; (2) the proposed TMLE approaches yield valid inference under the proposed MNAR assumptions; and (3) the proposed TMLE approaches remain robust against misspecification of either the (a) outcome or (b) exposure and missingness models.

### 5.1 Setup and design

In each simulated data set, *Y* was a fully observed binary outcome, *A* was a partially observed binary exposure, and *L*_*O*_ and *L*_*M*_ = (*L*_*M*1_, *L*_*M*2_) were common causes of {*A*, *Y*} that were fully and partially observed, respectively. We allowed for unmeasured common causes of {*L*_*O*_, *L*_*M*_}, {*L*_*M*_, *Y*} and {*L*_*O*_, *Y*}.

We generated 1,000 datasets of size *n* = 2,500 in three scenarios corresponding to different missingness mechanisms. In Scenario I, we observed {*A*, *L*_*M*_} when *R* = 1 (simultaneous missingness). This scenario satisfied the MAR assumption (see Figure 1[a]; *R* depended only on *L*_*O*_). We considered separate missingness mechanisms in Scenarios II–III. Scenario II satisfied $I_{A}$ (Figure 2[a]; included unmeasured common causes of {*A*, *R*_*A*_} and {*A*, *R*_*L*_}, and *R*_*A*_ depended on *L*_*M*_ and *A*), and scenario III satisfied $I_{B}$ (same as II except *R*_*L*1_ and *R*_*L*2_ are separately generated, and *R*_*L*2_ depended on *L*_*M*1_). In each case, the level of missingness was around 20–30 %. Full details are reported in Web Appendix M.

To each dataset we applied five estimators: (i) complete case analysis paired with complete-data TMLE (valid under Missing Completely at Random or MCAR; see Web Appendix J); (ii) MI (via mice package in R) paired with complete-data TMLE estimator (see Web Appendix F); (iii) an iterative conditional expectation (ICE [61]; see also Web Appendix G) estimator for each identifying formulae given throughout; (iv) an IPW estimator for each identifying formulae (see Web Appendix G); and (v) the proposed TMLEs. We compared bias, empirical standard error, and 95 % coverage probability (CP; based on nonparametric bootstrap with 1,000 resamples) of each estimator.

ICE estimators require a model for the outcome process, IPW estimators require models for the exposure and missingness processes, and the proposed TMLEs require all the aforementioned nuisance function models. To investigate robustness to misspecification of these working models, we considered each of these estimators when: (i) all models were correctly specified (i.e., the data-generating models were used), (ii) the outcome model was misspecified, and (iii) the exposure model was misspecified. Details regarding the specification of the parametric models used for the nuisance model can be found in the Web Appendix M. We applied TMLE-A in MAR Scenario I under the assumption that *R* = *R*_*A*_ = *R*_*L*_, which also satisfies the conditions defining $I_{A}$ . For all three scenarios, we also obtained results from TMLE using Highly Adaptive Lasso for fitting the nuisance functions (HAL [62]). Under the condition that the nuisance functions have finite sectional variation norm, HAL can estimate nuisance functional parameters at an approximate rate of *n* ^−1/3^ [63].

### 5.2 Simulation results

Table 3 shows results for simulation Scenarios I–III. As expected, biases from the complete case estimator are large in all scenarios. In MAR scenario I, MI is nearly unbiased and the confidence intervals cover roughly at nominal 95 % level. However, in all other scenarios (II–III), MI exhibits greater bias and poor coverage. In all scenarios, ICE is the most efficient compared with all other estimators.

**Table 3:** Results for simulations I–III for *n* = 2,500: Bias, standard error (SE), and 95 % confidence interval coverage probability (CP) all multiplied by 100. True value of *E*(*Y* ^*a* = 1^) = 0.288.

	(i) Correctly specified			(ii) Misspecified outcome model			(iii) Misspecified exposure model		
**I (MAR)**	Bias	SE	CP	Bias	SE	CP	Bias	SE	CP

CC	−1.77	2.84	85.5	−1.78	2.83	86.0	−1.77	2.85	85.7
MI	−0.04	2.36	95.5	0.00	2.36	95.4	−0.04	2.37	95.4
ICE-A	0.09	2.14	95.1	−2.71	1.89	68.1	0.09	2.14	95.1
IPW-A	0.11	2.56	94.4	0.11	2.56	94.4	−4.99	2.13	34.6
TMLE-A	0.12	2.55	94.5	0.11	2.56	94.5	0.11	2.38	94.4

**II** $\left(I_{A}\right)$	Bias	SE	CP	Bias	SE	CP	Bias	SE	CP

CC	1.85	1.79	81.8	1.76	1.81	83.0	1.85	1.79	81.8
MI	−1.12	1.50	86.5	−1.11	1.52	87.7	−1.12	1.50	86.6
ICE-A	0.04	1.78	93.7	−1.96	1.69	75.7	0.04	1.78	93.7
IPW-A	0.01	1.88	94.4	0.01	1.88	94.4	−2.60	1.80	65.3
TMLE-A	0.06	1.87	94.2	0.04	1.87	94.7	0.06	1.84	94.2

**III** $\left(I_{B}\right)$	Bias	SE	CP	Bias	SE	CP	Bias	SE	CP

CC	3.87	2.00	50.1	3.87	2.00	50.1	3.88	2.00	50.0
MI	−1.59	1.47	78.5	−1.59	1.47	79.3	−1.59	1.47	78.5
ICE-B	0.00	1.98	93.4	−1.63	1.91	83.3	0.00	1.98	93.4
IPW-B	−0.03	2.11	94.1	−0.03	2.11	94.1	−1.40	2.11	88.1
TMLE-B	0.03	2.09	93.7	0.01	2.10	93.7	0.03	2.10	93.9

When all models are correctly specified, IPW, ICE and TMLE are nearly unbiased across all scenarios. Moreover, as long as one set of nuisance function models (outcome models or exposure/missingness models) are correctly specified, TMLE remains nearly unbiased and achieves close to the nominal 95 % coverage. In contrast, ICE and IPW exhibit larger biases and lower coverage rates. In Scenarios I–III, TMLEs are at least as efficient as IPW when all the models are correctly specified, as expected from theory (see Remark 4). In Web Appendix M, we show an additional simulation study to illustrate efficiency gains discussed in Section 4 when it is reasonable to place variables in the order of increasing amount of missingness. For Scenarios I–III, the results from TMLE, with nuisance functions estimated using HAL, are given by: (Bias × 100, SE × 100, 95 % CP) = (0.12, 2.51, 93.7), (Bias × 100, SE × 100, 95 % CP) = (0.06, 1.86, 93.5), (Bias × 100, SE × 100, 95 % CP) = (0.04, 2.09, 93.5), respectively, where coverage probability is calculated based on the proposed asymptotic variance estimator.

## 6 Data analysis: opioid prescriptions and mortality among the elderly

The first wave of the opioid epidemic in the United States began in the late 1990s, which resulted in a rise of deaths attributed to prescription opioid overdoses [64]. Recent studies have shown that elderly patients are at a high risk of developing opioid use disorder, partly due to the challenges of pain management, leading to an increased risk of addiction and drug misuse in this population [65], 66]. Between 2010 and 2015, opioid-related hospitalization increased by 34 % among those 65 years and older [67]. Thus, quantifying the effects of opioid prescription on mortality among the elderly is thus an important public health goal.

Using data from the 1999–2004 cycles (i.e., during the first wave of the opioid epidemic) of the National Health and Nutrition Examination Survey (NHANES) study with linkage to mortality databases (National Death Index) through 2015, Inoue et al. [10] estimated the effect of opioid prescription on mortality during the 5-year follow-up after the NHANES household interview. As a case study, we investigated the effect of opioid prescription on all-cause death using the same data but restricted to those 65 years and older. Our sample contained *n* = 3,807 individuals, and included individuals’ prescription opioid use (*A* ∈ {0, 1}), all-cause mortality (*Y* ∈ {0, 1}), and covariates (*L*) including age, sex assigned at birth (male and female), race (non-Hispanic White, non-Hispanic Black, Mexican-American, or others), education levels (less than high school; high school or General Education Degree; more than high school), poverty-income ratio (the ratio of household income to the poverty threshold), health insurance coverage, marital status, smoking, alcohol intake, and chronic pain status.

As described in Inoue et al. [10], data on prescription medications used in the past 30 days were collected in the in-person interview. Those who responded ‘yes’ to using prescription medication were asked to show their medication containers to the interviewer: if the medication containers were not available, then the interviewer recorded the information verbally reported by the interviewee. Opioids identified through this process include codeine, fentanyl, oxycodone, pentazocine and morphine. About 6.5 % of the observed individuals in the sample reported using prescription opioids, and approximately 6 % of the individuals did not show their prescription medication container to the interviewer. Detailed data description can be found in Inoue et al. [10].

Complete outcome data were available, but data were partially missing for prescription opioid use (*n* = 43), education (*n* = 19), marital status (*n* = 117), poverty-income ratio (*n* = 410), smoking status (*n* = 10), alcohol intake (*n* = 257), health insurance coverage (*n* = 46), chronic pain status (*n* = 16), with 24 % of the subjects missing either the exposure or a confounder. We estimated the causal contrast *E*(*Y*) − *E*(*Y* ^*a* = 0^), which quantifies how the observed risk of death differs from the risk of death had no one taken prescription opioids. Since some participants might not have reported prescription opioids use despite taking them (possibly illicitly), we further conducted a sensitivity analysis in which subjects who did not show their prescription medication container to the interviewer were treated as having missing exposures (for a total of *n* = 242 missing exposure measurements).

Whereas Inoue et al. [10] handled missing data via MI, we compared: complete case analysis and MI (both followed by the usual complete-data TMLE), as well as our proposed TMLE estimators, as part of a sensitivity analysis. We conjecture that prescription opioid users may be less likely to report their true exposure status due to the nature of the in-person interview, hence the MAR assumption – which assumes *A* cannot affect *R*_*A*_ – may be unrealistic here. To address this, we applied TMLE-A under the $I_{A}$ assumption 5, treating all of *L*_*M*_ as unobserved when at least one of the covariates is unobserved. We further hypothesize that, in addition to demographic variables such as “age”, “sex” and “race” which are fully observed, “education” may also influence the observation of other potential confounders in *L*_*M*_. Thus, we also applied TMLE-B under the $I_{B}$ assumption 7 by including “education” as the first element of *L*_*M*_ to accommodate this conjecture. TMLE-B implicitly enforces a monotonic missingness in the covariates *L*_*M*_: here education is included first due to concerns outlined above. Absent any concerns about further confounders affecting missingness, the remaining confounders are included in the order of missingness. All 95 % confidence intervals (CIs) were based on the 2.5th and 97.5th percentiles of a nonparametric bootstrap procedure with 5,000 resamples.

We report results for the main and sensitivity analysis in Table 4. The MI estimate was very close to the complete case estimate (0.24 % vs. 0.23 %) and the 95 % CI of both estimators covered zero. That said, the MAR assumption may be violated here due to the sensitive nature of the exposure, and the TMLE estimates (under $I_{A}$ and $I_{B}$ ) were consistently twice as large, albeit with 95 % CIs that still covered zero. Even the largest estimate, that of TMLE-A, was small (0.62 %, 95 % CI: [−0.12, 1.39]), which is unsurprising given that we are comparing the risk of death had no one taken prescription opioids to the *observed* risk – and the observed prevalence of prescription opioid use was only 6.5 %. The sensitivity analyses showed a similar pattern of results but with larger effect estimates overall, with the TMLE effect estimate under $I_{A}$ as high as 0.98 % (95 % CI: 0.10 %, 1.83 %) and under $I_{B}$ as high as 0.92 % (95 % CI: 0.05 %, 1.74 %). In Web Appendix N, we provide results from TMLE when nuisance functions are estimated using HAL, which closely mirror those obtained here with parametric models.

**Table 4:** Results for data analysis using the NHANES study (three cycles from 1999 to 2004). CC denotes complete case analysis; ${\text{TMLE}}_{I_{A}}$ and ${\text{TMLE}}_{I_{B}}$ denote TMLE estimators for the identifying formulae under $I_{A}$ and $I_{B}$ , respectively. All results are multiplied by 100 (in %). All 95 % confidence intervals (CIs) were based on the 2.5th and 97.5th percentiles of a nonparametric bootstrap procedure with 5,000 resamples.

	Main analysis			
	CC	MI	${\mathrm{TMLE}}_{I_{A}}$	${\mathrm{TMLE}}_{I_{B}}$
Estimate 95 % CI	0.23 % (−0.19, 0.66)	0.21 % (−0.17, 0.65)	0.62 % (−0.12, 1.39)	0.55 % (−0.19, 1.31)

	Sensitivity analysis			
	CC	MI	${\mathrm{TMLE}}_{I_{A}}$	${\mathrm{TMLE}}_{I_{B}}$
Estimate 95 % CI	0.25 % (−0.20, 0.69)	0.20 % (−0.17, 0.71)	0.98 % (0.10, 1.83)	0.92 % (0.10, 1.77)

## 7 Discussion

Our work is motivated by missingness in both exposures and confounders, but it is nevertheless instructive to compare with methods for handling missinginess in one of these alone. When confounders alone are missing – and unless they can safely be assumed MAR – identifying average potential outcomes requires further assumptions. Some methods have relied on existence of a shadow variable [26] or on the assumption that missing covariates act as confounders only when they are observed [27], 28]. More commonly, methods have assumed that missingness is independent of the outcome [22] – however, this assumption alone is not sufficient for identification. Previous methods relying on this assumption further require a rank constraint – one that precludes the use of binary outcomes, and sometimes discrete outcomes altogether when there are multiple partially observed confounders [22], [23], [24], [25]. We too make an outcome-independence assumption, but drop the rank constraint in favour of a further assumption about the confounder missingness mechanism, which, as a consequence, allows our estimators to be applied to outcome types of any kind. The cost of this trade-off is that we make stricter assumptions about missingness in the confounders. Nevertheless, our proposed assumptions still permit certain missingness indicators to be associated with partially observed variables, offering a degree of flexibility not typically allowed under MAR. Future work will explore ways to integrate the self-censoring models [68] into the missingness assumptions for the *L*_*M*_ variables.

Note that the outcome-independence assumption may impose restrictions on the observed-data distribution (e.g., when outcome is continuous but covariates are discrete [69]). However, we share the same sentiment as Bartlett et al. [69], who write “we tend not to worry about [the potential testability of the outcome-independence assumption] in more realistic settings where power to refute the assumption will typically be very low”. As such, it may be possible to find semiparametric efficient influence functions by projecting the nonparametric efficient influence functions derived herein onto the tangent space of semiparametric models $\left(M_{semi*}\right)$ , which incorporates any restrictions on the observed data law imposed by our proposed assumptions. Estimators based on these semiparametric efficient influence functions will attain semiparametric efficiency bounds under $M_{semi*}$ , which may be smaller than the nonparametric efficiency bounds. We do not pursue this further in this paper and defer it as an open problem for future research.

The missingness assumptions and methods considered herein can also be adapted to data with missing values in all variables including the outcome (see Web Appendix L). When missingness is disjoint – i.e., some subjects are missing only exposure or only confounders – it is natural to treat *R*_*A*_ and *R*_*L*_ as distinct. But when *R*_*A*_ and *R*_*L*_ are highly correlated – i.e., most subjects are missing both or neither of *A* and *L*_*M*_ – analysts might opt to handle them as simultaneously missingness as in Section 3 (by treating both *A* and *L*_*M*_ as missing whenever *R*_*A*_ × *R*_*L*_ = 0). The advantages of this approach are that (a) one needs not worry about how observation indicators are related with one another, and (b) it may be easier to reason about the missingness mechanisms and requisite assumptions (i.e., via simpler DAGs). By contrast, treating missingness in the exposure and confounders separately as in Section 4 has the key advantage of loosening restrictions about the missingness mechanisms of the exposure variable. An analogous decision must be made about whether to treat missingness in different confounders separately. Treating confounders as either all missing or all observed as in Section 4.1 requires one to specify fewer missingness models, but treating them separately as in Section 4.2 allows for some partially missing confounders to affect whether other confounders are observed. In particular, analysts may order the elements of *L*_*M*_ to best leverage this additional flexibility. In general one need not choose between either extreme – lumping all incomplete data together, or treating each incomplete variable separately. One might instead opt for a balance of the two, for example treating education separately because it may affect missingness in other covariates, while treating all other covariates as entirely observed or missing simultaneously. In practice, we suggest that it may be sensible to apply the various TMLE methods described herein to a data set as a simple form of sensitivity analysis. This can help determine whether the overall findings are robust to different methodological approaches.

Our simulation studies show that all proposed estimators yield valid inference, and ICE estimators are most efficient, when models are correctly specified. We also find that when models may be misspecified, the proposed TMLEs provide more than one chance to achieve valid inference. In practice, nuisance models are generally unknown, and machine learning algorithms can be used to estimate the nuisance functions in any of the TMLE estimators. These estimators can achieve $\sqrt{n}$ -consistency and asymptotic normality provided that the estimators of nuisance functions are consistently estimated at a rate faster than *n* ^−1/4^ [70], 71], and our simulations indicated good performance in finite samples. We note, however, that consistency is not guaranteed when more flexible algorithms such as neural network or random forest are used. In these cases – when standard Donsker conditions are not met – sample splitting and cross-fitting can be used to satisfy necessary empirical process conditions [72], 73].

In this work, we rely on the conditional exchangeability assumption as a sufficient condition for identifying our causal estimand *E*(*Y* ^*a*^). However, weaker or alternative assumptions, such as those in proximal learning [74], may be applicable depending on the setting and available information. Addressing missing data under such frameworks is an important direction for future research.

## Supplementary Material

Supplementary Material

## References

[1] Taubman SL, Robins JM, Mittleman MA, Hernán MA (2009). Intervening on risk factors for coronary heart disease: an application of the parametric g-formula. Int J Epidemiol.

[2] Lajous M, Willett WC, Robins J, Young JG, Rimm E, Mozaffarian D (2013). Changes in fish consumption in midlife and the risk of coronary heart disease in men and women. Am J Epidemiol.

[3] Lodi S, Phillips A, Logan R, Olson A, Costagliola D, Abgrall S (2015). Comparative effectiveness of immediate antiretroviral therapy versus CD4-based initiation in HIV-positive individuals in high-income countries: observational cohort study. Lancet HIV.

[4] Hernán MA, Brumback B, Robins JM (2000). Marginal structural models to estimate the causal effect of zidovudine on the survival of HIV-positive men. Epidemiology.

[5] Cain L, Robins JM, Lanoy E, Logan R, Costagliola D, Hernán MA (2010). When to start treatment? A systematic approach to the comparison of dynamic regimes using observational data. Int J Biostat.

[6] Neugebauer R, Schmittdiel JA, Van Der Laan MJ (2014). Targeted learning in real-world comparative effectiveness research with time-varying interventions. Stat Med.

[7] Bang H, Robins JM (2005). Doubly robust estimation in missing data and causal inference models. Biometrics.

[8] Tran L, Yiannoutsos C, Wools-Kaloustian K, Siika A, Van Der Laan M, Petersen M (2019). Double robust efficient estimators of longitudinal treatment effects: comparative performance in simulations and a case study. Int J Biostat.

[9] Wen L, Marcus JL, Young JG (2023). Intervention treatment distributions that depend on the observed treatment process and model double robustness in causal survival analysis. Stat Methods Med Res.

[10] Inoue K, Ritz B, Arah OA (2022). Causal effect of chronic pain on mortality through opioid prescriptions: application of the front-door formula. Epidemiology.

[11] Jane Williamson E, Forbes A, Wolfe R (2012). Doubly robust estimators of causal exposure effects with missing data in the outcome, exposure or a confounder. Stat Med.

[12] Zhang Z, Liu W, Zhang B, Tang L, Zhang J (2016). Causal inference with missing exposure information: methods and applications to an obstetric study. Stat Methods Med Res.

[13] Kennedy EH (2020). Efficient nonparametric causal inference with missing exposure information. Int J Biostat.

[14] Sperrin M, Martin GP (2020). Multiple imputation with missing indicators as proxies for unmeasured variables: simulation study. BMC Med Res Methodol.

[15] Qu Y, Lipkovich I (2009). Propensity score estimation with missing values using a multiple imputation missingness pattern (MIMP) approach. Stat Med.

[16] Crowe BJ, Lipkovich IA, Wang O (2010). Comparison of several imputation methods for missing baseline data in propensity scores analysis of binary outcome. Pharm Stat.

[17] Mitra R, Reiter JP (2011). Estimating propensity scores with missing covariate data using general location mixture models. Stat Med.

[18] Seaman S, White I (2014). Inverse probability weighting with missing predictors of treatment assignment or missingness. Commun Stat Theor Methods.

[19] Evans K, Fulcher I, Tchetgen Tchetgen EJ (2020). A coherent likelihood parametrization for doubly robust estimation of a causal effect with missing confounders. ..

[20] Ross RK, Breskin A, Breger TL, Westreich D (2022). Reflection on modern methods: combining weights for confounding and missing data. Int J Epidemiol.

[21] D’Agostino RB, Rubin DB (2000). Estimating and using propensity scores with partially missing data. J Am Stat Assoc.

[22] Ding P, Geng Z (2014). Identifiability of subgroup causal effects in randomized experiments with nonignorable missing covariates. Stat Med.

[23] Yang S, Wang L, Ding P (2019). Causal inference with confounders missing not at random. Biometrika.

[24] Guan Q, Yang S (2019). A unified framework for causal inference with multiple imputation using martingale. ..

[25] Sun Z, Liu L (2021). Semiparametric inference of causal effect with nonignorable missing confounders. Stat Sin.

[26] Miao W, Tchetgen Tchetgen E (2018). Identification and inference with nonignorable missing covariate data. Stat Sin.

[27] Blake HA, Leyrat C, Mansfield KE, Tomlinson LA, Carpenter J, Williamson EJ (2020). Estimating treatment effects with partially observed covariates using outcome regression with missing indicators. Biom J.

[28] Blake HA, Leyrat C, Mansfield KE, Seaman S, Tomlinson LA, Carpenter J (2020). Propensity scores using missingness pattern information: a practical guide. Stat Med.

[29] Lu B, Ashmead R (2018). Propensity score matching analysis for causal effects with MNAR covariates. Stat Sin.

[30] Mattei A (2009). Estimating and using propensity score in presence of missing background data: an application to assess the impact of childbearing on wellbeing. Stat Methods Appl.

[31] Levis AW, Mukherjee R, Wang R, Haneuse S (2025). Robust causal inference for point exposures with missing confounders. Can J Stat.

[32] Rubin DB (1976). Inference and missing data. Biometrika.

[33] Seaman S, Galati J, Jackson D, Carlin J (2013). What is meant by “missing at random”?. Stat Sci.

[34] Mohan K, Pearl J (2021). Graphical models for processing missing data. J Am Stat Assoc.

[35] Scharfstein DO, Rotnitzky A, Robins JM (1999). Adjusting for nonignorable drop-out using semiparametric nonresponse models. J Am Stat Assoc.

[36] Paul RR, Rubin DB (1984). Reducing bias in observational studies using subclassification on the propensity score. J Am Stat Assoc.

[37] Mohan K, Pearl J, Tian J (2013). Graphical models for inference with missing data. Advances in neural information processing systems.

[38] Mohan K, Pearl J, Ghahramani Z, Welling M, Cortes C, Lawrence N, Weinberger KQ (2014). Graphical models for recovering probabilistic and causal queries from missing data. Advances in neural information processing systems.

[39] Mohan K, Pearl J (2014). On the testability of models with missing data. Artificial intelligence and statistics.

[40] Bhattacharya R, Nabi R, Shpitser I, Robins JM (2020). Identification in missing data models represented by directed acyclic graphs. Uncertainty in artificial intelligence.

[41] Moreno-Betancur M, Lee KJ, Leacy FP, White IR, Simpson JA, Carlin JB (2018). Canonical causal diagrams to guide the treatment of missing data in epidemiologic studies. Am J Epidemiol.

[42] Rubin DB (1980). Randomization analysis of experimental data: the fisher randomization test comment. J Am Stat Assoc.

[43] Lopoo LM, Western B (2005). Incarceration and the formation and stability of marital unions. J Marriage Fam.

[44] Leslie HH, Karasek DA, Harris LF, Chang E, Abdulrahim N, Maloba M (2014). Cervical cancer precursors and hormonal contraceptive use in HIV-positive women: application of a causal model and semi-parametric estimation methods. PLoS One.

[45] Lippold MA, Coffman DL, Greenberg MT (2014). Investigating the potential causal relationship between parental knowledge and youth risky behavior: a propensity score analysis. Prev Sci.

[46] Kreif N, Gruber S, Radice R, Grieve R, Sekhon JS (2016). Evaluating treatment effectiveness under model misspecification: a comparison of targeted maximum likelihood estimation with bias-corrected matching. Stat Methods Med Res.

[47] Decruyenaere A, Steen J, Colpaert K, Benoit DD, Decruyenaere J, Vansteelandt S (2020). The obesity paradox in critically ill patients: a causal learning approach to a casual finding. Crit Care.

[48] Anthony ER, Cho Y, Fischer RL, Matthews L (2021). Examining the causal impact of prenatal home visiting on birth outcomes: a propensity score analysis. Matern Child Health J.

[49] Ghazaleh Dashti S, Lee KJ, Simpson JA, White IR, Carlin JB, Moreno-Betancur M (2021). Handling missing data when estimating causal effects with targeted maximum likelihood estimation. ..

[50] Robins JM, Gill RD (1997). Non-response models for the analysis of non-monotone ignorable missing data. Stat Med.

[51] Vansteelandt S, Rotnitzky A, Robins JM (2007). Estimation of regression models for the mean of repeated outcomes under nonignorable nonmonotone nonresponse. Biometrika.

[52] Sun BL, Tchetgen EJT (2018). On inverse probability weighting for nonmonotone missing at random data. J Am Stat Assoc.

[53] Srinivasan R, Bhattacharya R, Nabi R, Ogburn EL, Shpitser I (2023). Graphical models of entangled missingness. ..

[54] van der Laan MJ, Rose S (2011). Targeted learning: causal inference for observational and experimental data.

[55] Little RJA, Rubin DB (2002). Statistical analysis with missing data.

[56] Zhao J, Shao J (2015). Semiparametric pseudo-likelihoods in generalized linear models with nonignorable missing data. J Am Stat Assoc.

[57] Bia M, Huber M, Lafférs L (2024). Double machine learning for sample selection models. J Bus Econ Stat.

[58] de Aguas J, Pensar J, Pérez TV, Biele G (2025). Recovery and inference of causal effects with sequential adjustment for confounding and attrition. J Causal Inference.

[59] Zhou Y, Little RJA, Kalbfleisch JD (2010). Block-conditional missing at random models for missing data. Stat Sci.

[60] Tsiatis AA (2006). Semiparametric theory and missing data.

[61] Wen L, Young JG, Robins JM, Hernán MA (2021). Parametric g-formula implementations for causal survival analyses. Biometrics.

[62] Hejazi NS, Coyle JR, van der Laan MJ (2020). hal9001: scalable highly adaptive lasso regression in R. J Open Source Software.

[63] van der Laan M (2017). A generally efficient targeted minimum loss based estimator based on the highly adaptive lasso. Int J Biostat.

[64] Dowell D, Haegerich TM, Chou R (2016). CDC guideline for prescribing opioids for chronic pain—United States, 2016. JAMA.

[65] Guerriero F (2017). Guidance on opioids prescribing for the management of persistent non-cancer pain in older adults. World J Clin Cases.

[66] Dufort A, Samaan Z (2021). Problematic opioid use among older adults: epidemiology, adverse outcomes and treatment considerations. Drugs Aging.

[67] Weiss AJ, Heslin KC, Barrett ML, Izar R, Bierman AS (2018). Opioid-related inpatient stays and emergency department visits among patients aged 65 years and older, 2010 and 2015.

[68] Li Y, Miao W, Shpitser I, Tchetgen EJT (2023). A self-censoring model for multivariate nonignorable nonmonotone missing data. Biometrics.

[69] Bartlett JW, Carpenter JR, Tilling K, Vansteelandt S (2014). Improving upon the efficiency of complete case analysis when covariates are MNAR. Biostatistics.

[70] Robins J, Li L, Tchetgen Tchetgen E, Van Der Vaart A (2008). Higher order influence functions and minimax estimation of nonlinear functionals. Probability and statistics: essays in honor of David A. Freedman.

[71] Robins JM, Li L, Tchetgen Tchetgen E, Van Der Vaart A (2016). Asymptotic normality of quadratic estimators. Stochastic Process Appl.

[72] Van Der Vaart AW (2000). Asymptotic statistics.

[73] Chernozhukov V, Chetverikov D, Demirer M, Duflo E, Hansen C, Newey W (2018). Double/debiased machine learning for treatment and structural parameters. Econometrics J.

[74] Tchetgen Tchetgen EJ, Ying A, Cui Y, Shi X, Miao W (2020). An introduction to proximal causal learning. ..

