# Supplementary material for: Estimating average causal effects with incomplete exposure and confounders
Source: J Causal Inference. 2026 Feb 20;14(1):20230083. doi: 10.1515/jci-2023-0083 (PMC12922761; doi:10.1515/jci-2023-0083)
Supplement: Supplementary file 1 — Supplementary Material [file j_jci-2023-0083_suppl_001.pdf]

WEB APPENDIX FOR "ESTIMATING AVERAGE CAUSAL EFFECTS WHEN EXPOSURE AND CONFOUNDERS ARE MISSING"

Contents

A Simultaneous Missingness 3

A.1 Identification 3

A.2 Estimation 3

B Additional DAGs 5

C Proof of identification 6

C.1 Identification under Assumption 3 (MAR) 6

C.2 Identification under Assumption A.1 ($\mathcal{I}_{joint}$) 6

C.3 Identification under Assumption 5 ($\mathcal{I}_A$) 7

C.4 Identification under Assumption 7 ($\mathcal{I}_B$) 7

D Alternative Assumptions 8

D.1 An alternative MNAR assumption to $\mathcal{I}_A$ 8

E Derivation of efficient influence functions 10

E.1 Efficient influence function under Assumption A.1 ($\mathcal{I}_{joint}$) 10

E.2 Efficient influence function under Assumption 5 ($\mathcal{I}_A$) 12

E.3 Efficient influence function under Assumption 7 ($\mathcal{I}_B$) 12

E.4 Efficient influence function under Assumption C.3 ($\mathcal{I}_C$) 14

E.5 Efficient influence function under Assumption 3 (MAR) 15

F Consistency of multiple imputation under MAR 17

G ICE and IPW Estimators 19

G.1 ICE Estimator Under Assumption A.1 $\mathcal{I}_{Joint}$ 19

G.2 IPW Estimator Under Assumption A.1 $\mathcal{I}_{Joint}$ 19

G.3 ICE Estimator Under Assumption 5 $\mathcal{I}_A$ 20

G.4 IPW Estimator Under Assumption 5 $\mathcal{I}_A$ 20

G.5 ICE Estimator Under Assumption 7 $\mathcal{I}_B$ 21

G.6 IPW Estimator Under Assumption 7 $\mathcal{I}_B$ 21

G.7 ICE Estimator Under Assumption C.3 $\mathcal{I}_C$ 21

G.8 IPW Estimator Under Assumption C.3 $\mathcal{I}_C$ 22

H Asymptotic properties of TMLE 23

I Proof of multiple robustness 25

I.1 Proof of double robustness for $\mathcal{I}_{joint}$ and $\mathcal{I}_A$ 25

I.2 Proof of double robustness for $\mathcal{I}_B$ 26

I.3 Proof of double robustness for $\mathcal{I}_C$ 26

I.4 Additional robustness against model misspecification 28

J Complete case analysis 30

K Missing Exposure Only 31

K.1 Missingness mechanism assumptions associated with missing exposure or treatment variable 31

K.2 DAGs with Missing Treatment 32

K.3 TMLE estimator under treatment-outcome conditional independence 34

L Missing values in all variables 35

L.1 Simultaneous missingness 35

L.2 Separating observation indicators 35

M Simulation details and additional simulation studies 37

M.1 Additional simulation study to show efficiency gain in TMLE-B 38

M.2 Additional simulation study under $\mathcal{I}_C$ 39

N Additional results from data analysis 39

A. Simultaneous Missingness

In the main text, we considered the missing at random (MAR) assumption for $R{=}R_AR_L$. Now we now consider a set of assumptions that do not satisfy MAR and hence correspond to MNAR in the missing data literature (Little and Rubin, 2002) in this context. The observed data for an individual are $O{=}(L_O,R,RL_M,RA,Y)$.

A.1 Identification

*Assumption A.*1 ($\mathcal{I}_{Joint}$). $R \perp\!\!\!\perp Y|A,L$, and $R \perp\!\!\!\perp L_M|L_O$.

*Assumption A.*2 (Positivity: $\mathcal{I}_{Joint}$).

$$P(A{=}a|R{=}1,L{=}l){>}0,\ \ \forall l{\in}\text{supp}(L),\ \text{and}\ P(R{=}1|L_O{=}l_O){>}0,\ \ \forall l_O{\in}\text{supp}(L_O).$$

Under assumptions A.1 and A.2, $E(Y^a)$ is identified by the following formula:

$$\Psi^a_{\mathcal{I}_{joint}}{=}\sum_l E(Y|A{=}a,R{=}1,L)p(l_M|l_O,R{=}1)p(l_O) \tag{A.1}$$

The DAGs in Figure 1 satisfy $\mathcal{I}_{joint}$ assumption A.1 but fail to satisfy MAR assumption 3 because they include unmeasured common causes of exposure A and missingness R. This might occur in an observational study if exposure information is sensitive, such as opioid intake. For instance, it is possible that an individual's perception to certain social factors (e.g., societal and cultural norms) can affect whether they take opioids (exposure) and whether they report their exposure status.

[Figure 1 about here.]

We note that the outcome-independence assumption precludes Y from affecting R and hence the MAR scenarios when A and L_M are observed prior to occurrence of the outcome also satisfy $\mathcal{I}_{joint}$ assumptions A.1 (e.g., Figure 1[a]). In contrast, the DAG in Figure 1[b] for MAR allows the outcome to affect missingness, hence $\mathcal{I}_{joint}$ assumption A.1 is not a strict generalization of the MAR assumption 3.

A.2 Estimation When the MAR assumption does not hold, we propose consistent estimators based on efficient influence functions under $\mathcal{I}_{joint}$ assumption A.1. The efficient influence function for $\Psi^a_{\mathcal{I}_{joint}}$ given by identifying formula (A.1) under $\mathcal{M}_{np}$ equals:

$$\phi^1_{P_{\mathcal{I}_{\mathbf{joint}}}}(O){=}\frac{I(A{=}a,R{=}1)}{\pi_A(L)\pi_R(L_O)}\{Y{-}T_1(L)\}{+}\frac{I(R{=}1)}{\pi_R(L_O)}\{T_1(L){-}T_0(L_O)\}{+}T_0(L_O){-}\Psi^a_{\mathcal{I}_{joint}} \tag{A.2}$$

where $\pi_A(L){=}P(A{=}a|R{=}1,L)$, $\pi_R(L_O){=}P(R{=}1|L_O)$, $T_1(L){=}E(Y|A{=}a,R{=}1,L)$ and $T_0(L_O){=}E(T_1(L)|R{=}1,L_O)$ (see Web Appendix E for derivation). With the efficient influence function in hand, we can construct consistent estimators of $\Psi^a_{\mathcal{I}_{joint}}$, such as the solution

to estimating equations or the TMLE. We propose a TMLE via Algorithm 1, which conveniently reduces to the complete-data TMLE when there is no missing data (van der Laan and Rose, 2011). Throughout, we will use $\mathcal{M}$ with subscripts "T_1" and T_0, and "π_A" and "π_L to denote models with the correct specification of the outcomes defined by T-functions, and the propensity score and missingness probabilities, respectively.

Algorithm 1 Algorithm for TMLE-Joint under $\mathcal{I}_{joint}$

1: Obtain estimates $\hat{\pi}_A(L)$ and $\hat{\pi}_R(L_O)$ of $\pi_A(L)$ and $\pi_R(L_O)$, respectively.
2: *Obtain Initial Estimate of* $T_1(L)$. Among subjects with $R{=}1$, fit a regression model $\eta_1(A,L;\kappa_1){=}g^{-1}\{(A,L)'\kappa_1\}$ by regressing Y on A and L, where κ_1 is a vector of parameters to be estimated and g^{-1} denotes a known inverse link function (alternatively, regress Y on L among those with $A{=}a$).
3: *Targeting Step for* $T_1(L)$. Among those with $A{=}a$ and $R{=}1$, fit an intercept-only regression model for Y with weight $\{\hat{\pi}_A(L)\hat{\pi}_R(L_O)\}^{-1}$ and an offset given by $g\{\eta_1(A,L;\hat{\kappa}_1)\}$, i.e., solve for ϵ_1 in

$$\mathbb{P}_n\left\{\frac{I(A{=}a,R{=}1)}{\hat{\pi}_A(L)\hat{\pi}_R(L_O)}\big(Y-g^{-1}[g\{\eta_1(A,L;\hat{\kappa}_1)\}+\epsilon_1]\big)\right\}=0$$

4: Among those with $R{=}1$, predict $T_1(L)$ using $\hat{T}_1(L){=}g^{-1}[g\{\eta_1(A{=}a,L;\hat{\kappa}_1)\}+\hat{\epsilon}_1]$.
5: *Obtain Initial Estimate of* $T_0(L_O)$. Among those with $R{=}1$, fit a regression model $\eta_0(L_O;\kappa_0){=}g^{-1}(L_O'\kappa_0)$ by regressing $\hat{T}_1(L)$ on L_O, where κ_0 is a vector of parameters to be estimated.
6: *Targeting Step for* $T_0(L_O)$. Among those with $R{=}1$, fit an intercept-only regression model for $\hat{T}_1(L)$ with weight $\hat{\pi}_R(L_O)^{-1}$ and an offset given by $g\{\eta_0(L_O;\hat{\kappa}_0)\}$, i.e., solve for ϵ_0 in

$$\mathbb{P}_n\left\{\frac{I(R{=}1)}{\hat{\pi}_R(L_O)}\Big(\hat{T}_1(L)-g^{-1}[g\{\eta_0(L_O;\hat{\kappa}_0)\}+\epsilon_0]\Big)\right\}=0$$

7: Predict $T_0(L_O)$ using $\hat{T}_0(L_O){=}g^{-1}[g\{\eta_0(L_O;\hat{\kappa}_0)\}+\hat{\epsilon}_0]$ for all observations.
8: Calculate the TMLE-Joint estimator $\widehat{\Psi}^a_{\mathbf{TMLE},\mathcal{I}_\mathbf{A}}{=}\mathbb{P}_n\{\hat{T}_0(L_O)\}$.

Proposition A.1. The proposed TMLE-Joint is consistent for $\Psi^a_{\mathcal{I}_{joint}}$ under $(\mathcal{M}_{T_1}\cap\mathcal{M}_{T_0})\cup(\mathcal{M}_{\pi_A}\cap\mathcal{M}_{\pi_R})$.

B. Additional DAGs

We present additional DAGs that satisfy the missingness assumptions discussed in the main manuscript. Figures 2 and 3 consist of DAGs that satisfy Assumption 3 from the main manuscript. Figure 4 consists of DAGs that satisfy Assumption A.1 from the main manuscript. Figure 5 consists of DAGs that satisfy Assumption 5 from the main manuscript.

[Figure 2 about here.]

[Figure 3 about here.]

[Figure 4 about here.]

[Figure 5 about here.]

C. Proof of identification

Under consistency and outcome-treatment exchangeability, the average potential outcome if, possibly contrary to fact, the treatment had taken a value a is given by:

$$E(Y^a)=\sum_l E(Y|A=a,L=l)p(l_O,l_M).$$

When there are missingness in exposure and confounders, the identifying formula depends on the missingness assumptions.

C.1 Identification under Assumption 3 (MAR)

$$\begin{aligned}E(Y^a)&=\sum_l E(Y|A=a,L=l)p(l_O,l_M)\\&=E\left\{\sum_y y\frac{p(y,a,L)}{p(a,L)}\right\}\\&=E\left\{\frac{\sum_y yp(L_M,a|L_O,y,R=1)p(y|L_O)}{\sum_{y'}p(L_M,a|L_O,y',R=1)p(y'|L_O)}\right\}\\&=E\left\{\frac{\beta(L)}{\gamma(L)}\frac{R}{P(R=1|L_O,Y)}\right\}\end{aligned}$$

where as before, $\beta(L)=\sum_y yp(L_M,a|L_O,y,R=1)p(y|L_O)$ and $\gamma(L)=\sum_{y'}p(L_M,a|L_O,y',R=1)p(y'|L_O)$. Here, we used the fact that:

$$\begin{aligned}p(y,a,l)&=\frac{P(R=1,Y=y,A=a,L=l)}{P(R=1|Y=y,A=a,L=l)}\\&=\frac{p(l_M,a|l_O,y,R=1)p(l_O,y,R=1)}{P(R=1|Y=y,L_O=l_O)}\\&=p(l_M,a|l_O,y,R=1)p(l_O,y)\end{aligned}$$

Thus, it follows that: $p(a,l)=\sum_y p(l_M,a|l_O,y,R=1)p(l_O,y)$.

C.2 Identification under Assumption A.1 ($\mathcal{I}_{joint}$)

$$\begin{aligned}E(Y^a)&=\sum_l E(Y|A=a,L=l)p(l_O,l_M)\\&=\sum_l E(Y|A=a,L=l,R=1)p(l_O,l_M)\\&=\sum_l E(Y|A=a,L=l,R=1)p(l_M|l_O,R=1)p(l_O)\end{aligned}$$

C.3 Identification under Assumption 5 ($\mathcal{I}_A$)

$$\begin{aligned}E(Y^a)&=\sum_l E(Y|A=a,L=l)p(l_O,l_M)\\&=\sum_l E(Y|A=a,L=l,R_A=1,R_L=1)p(l_O,l_M)\\&=\sum_l E(Y|A=a,L=l,R=1)p(l_M|l_O,R_L=1)p(l_O)\end{aligned}$$

C.4 Identification under Assumption 7 ($\mathcal{I}_B$) In assumption 7, we assume $R_{Lk}\perp\!\!\!\perp \underline{L}_{Mk}|\bar{R}_{L,k-1},\bar{L}_{M,k-1},L_O,\ \forall k=1,\ldots,q$.

It is straightforward to show that $R_{Lk}\perp\!\!\!\perp \underline{L}_{Mk}|\bar{R}_{L,k-1},\bar{L}_{M,k-1},L_O,\ \forall k=1,\ldots,q$ implies:

$$P(R_{Lk}=1|\bar{R}_{L,k-1}=1_{k-1},\bar{L}_M,L_O)=P(R_{Lk}=1|\bar{R}_{L,k-1}=1_{k-1},\bar{L}_{M,k-1},L_O),$$

which also is equivalent to $P(\bar{R}_{Lk}=1_k|\bar{L}_M,L_O)=P(\bar{R}_{Lk}=1_k|\bar{L}_{M,k-1},L_O)$. This follows directly via induction (see Wen *and others* (2017) for a similar proof). By Bayes Rule, it can be shown that

$$P(\bar{R}_{Lk}=1_k|\bar{L}_M=\bar{l}_M,L_O=l_O)=P(\bar{R}_{Lk}=1_k|\bar{L}_{M,k-1}=l_{M,k-1},L_O=l_O),\ \ \forall k$$

is equivalent to: $p(l_{Mk},\ldots,l_{Mq}|\bar{l}_{M,k-1},\bar{R}_{Lk}=1_k,l_O)=p(l_{Mk},\ldots,l_{Mq}|\bar{l}_{M,k-1},l_O),\ \forall k$. As such, it then follows that

$$p(l_{Mk}|\bar{l}_{M,k-1},\bar{R}_{Lk}=1_k,l_O)=p(l_{Mk}|\bar{l}_{M,k-1},l_O),\ \ \forall k$$

We continue the proof as follows:

$$\begin{aligned}E(Y^a)&=\sum_l E(Y|A=a,L=l)p(l_O,l_M)\\&=\sum_l E(Y|A=a,L=l,R_A=1,R_L=1)p(l_O,l_M)\\&=\sum_l E(Y|A=a,L=l,R=1)p(l_{M1},\ldots,l_{Mq}|l_O)p(l_O)\\&=\sum_l E(Y|A=a,L=l,R=1)p(l_{Mq}|\bar{l}_{M,q-1},l_O,\bar{R}_{Lq}=1_q)p(\bar{l}_{M,q-1}|l_O)p(l_O)\\&=\ldots\\&=\sum_l E(Y|A=a,L=l,R=1)\prod_{k=2}^{q}p(l_{Mk}|\bar{l}_{M,k-1},l_O,\bar{R}_{Lk}=1_k)p(\bar{l}_{M1}|l_O)p(l_O)\\&=\sum_l E(Y|A=a,L=l,R=1)\prod_{k=2}^{q}p(l_{Mk}|\bar{l}_{M,k-1},l_O,\bar{R}_{Lk}=1_k)p(\bar{l}_{M1}|l_O,R_{L1}=1)p(l_O)\\&=\sum_l E(Y|A=a,L=l,R=1)\prod_{k=1}^{q}p(l_{Mk}|\bar{l}_{M,k-1},l_O,\bar{R}_{Lk}=1_k)p(l_O)\end{aligned}$$

D. Alternative Assumptions

D.1 An alternative MNAR assumption to $\mathcal{I}_A$ An alternative set of MNAR assumptions is:

*Assumption D.*3 ($\mathcal{I}_C$). $(R_A,R_L) \perp\!\!\!\perp Y|A,L$ and $R_L \perp\!\!\!\perp L_M|L_O,R_A$.

*Assumption D.*4 (Positivity: $\mathcal{I}_C$). $P(A{=}a|R{=}1,L{=}l){>}0$, $\forall l{\in}\text{supp}(L)$, and $P(R_L{=}1|L_O{=}l_O,R_A{=}r_A){>}0$, $\forall (l_O,r_A){\in}\text{supp}(L_O,R_A)$,

Under Assumptions C.3 and 4, $E(Y^a)$ can be identified by the following formula:

$$\Psi^a_{\mathcal{I}_C}{=}\sum_l E(Y|A{=}a,R{=}1,L)\sum_{r_A} p(l_M|r_A,l_O,R_L{=}1)p(r_A|l_O)p(l_O) \tag{D.3}$$

Assumptions 5 and C.3 differ in that in Assumption C.3, if R_A is affected by exposure or exposure status A and/or missing covariates L_M, we allow R_A to also affect R_L. This may be reasonable in the following context: suppose that taking over-the-counter medication prompts individuals to follow-up with their healthcare provider such that R_A (whether exposure is observed by the healthcare provider) depends on A. In this scenario, it is reasonable to assume that a healthcare provider may ask for more information on a patient's history or baseline characteristics if the healthcare provider observed their patient's exposure status. Consequently R_L will depend on R_A and $R_L \not\perp\!\!\!\perp L_M|L_O$, but it may be possible that $R_L \perp\!\!\!\perp L_M|L_O,R_A$ (See Figure 6). Another example could be in survey studies where a question on a covariate in L could depend on whether a subject answered a question pertaining to the exposure A.

[Figure 6 about here.]

Estimation under Assumption D.3 ($\mathcal{I}_C$)

Next we consider identifying formula (D.3) under Assumption C.3. The efficient influence function for $\Psi^a_{\mathcal{I}_C}$ in identifying formula (D.3) equals:

$$\begin{aligned}\phi^1_{P_{\mathcal{I}_C}}(O){=}&\frac{I(A{=}a,R_A{=}1,R_L{=}1)}{\pi_A(L)\pi_{R_A}(L)}\left\{\sum_{r_A}\frac{P(R_A{=}r_A|L,R_L{=}1)}{P(R_L{=}1|L_O,R_A{=}r_A)}\right\}\{Y-T_1(L)\}\\&+\frac{I(R_L{=}1)}{P(R_L{=}1|L_O,R_A)}\{T_1(L)-\widetilde{T}_0(L_O)\}+\widetilde{T}_0(L_O)-\Psi^a_{\mathcal{I}_C}\end{aligned} \tag{D.4}$$

where $T_1(L){=}E(Y|A{=}a,L,R_A{=}1,R_L{=}1)$, and $\widetilde{T}_0(L_O){=}E(T_1(L)|L_O,R_A,R_L{=}1)$. We compute the TMLE based on this efficient influence function via Algorithm 2.

Algorithm 2 Algorithm for TMLE-C under $\mathcal{I}_C$

1: Obtain estimates of $\hat{\pi}_A(L)$, $\hat{\pi}_{R_A}(L)$ and $\hat{P}(R_L{=}1|L_O,R_A)$ of $\pi_A(L)$, $\pi_{R_A}(L)$ and $P(R_L{=}1|L_O,R_A)$.

2: Among those with $R{=}1$, fit a regression model $\eta_1(A,L;\kappa_1){=}g^{-1}([A,L]'\kappa_1)$ by regressing Y on A and L.
3: Among those with $(A,R){=}(a,1)$, regress Y on an intercept with observational weight $\{\hat{\pi}_A(L)\hat{\pi}_{R_A}(L)\}^{-1}\left[\sum_{r_A}\frac{r_A\hat{\pi}_{R_A}(L)+(1-r_A)(1-\hat{\pi}_{R_A}(L))}{\hat{P}(R_L=1|L_O,R_A=r_A)}\right]$ and an offset given by $g\{\eta_1(A,L_M,L_O;\hat{\kappa}_1)\}$, i.e., solve for ϵ_1 in

$$\mathbb{P}_n\left\{\frac{I(A{=}a,R{=}1)}{\hat{\pi}_A(L)\hat{\pi}_{R_A}(L)}\left[\sum_{r_A}\frac{r_A\hat{\pi}_{R_A}(L)+(1-r_A)(1-\hat{\pi}_{R_A}(L))}{\hat{P}(R_L{=}1|L_O,R_A{=}r_A)}\right]\left(Y-g^{-1}[g\{\eta_1(A,L;\hat{\kappa}_1)\}+\epsilon_1]\right)\right\}{=}0$$

4: Among those with $R_L{=}1$, predict $T_1(L)$ using $\hat{T}_1(L){=}g^{-1}[g\{\eta_1(A{=}a,L;\hat{\kappa}_1)\}+\hat{\epsilon}_1]$.
5: Among those with $R_L{=}1$, fit a regression model $\eta_0(L_O,R_A;\kappa_0){=}g^{-1}([L_O,R_A]'\kappa_0)$ by regressing $\hat{T}_1(L)$ on L_O and R_A.
6: Among those with $R_L{=}1$, regress $\hat{T}_1(L)$ on an intercept with observational weight $\hat{P}(R_L{=}1|L_O,R_A)^{-1}$ and an offset given by $g\{\eta_0(L_O,R_A;\hat{\kappa}_0)\}$, i.e., solve for ϵ_0 in

$$\mathbb{P}_n\left\{\frac{I(R_L{=}1)}{\hat{P}(R_L{=}1|L_O,R_A)}\left(\hat{T}_1(L)-g^{-1}[g\{\eta_0(L_O,R_A;\hat{\kappa}_0)\}+\epsilon_0]\right)\right\}{=}0$$

7: Predict $\widetilde{T}_0(L_O)$ using $\widehat{\widetilde{T}}_0(L_O){=}g^{-1}[g\{\eta_0(L_O,R_A;\hat{\kappa}_0)\}+\hat{\epsilon}_0]$ for all observations.
8: Calculate the TMLE-C estimator $\widehat{\Psi}^a_{\mathbf{TMLE},\mathcal{I}_\mathbf{C}}{=}\mathbb{P}_n(\widehat{\widetilde{T}}_0(L_O))$.

Remark 1. Under $\mathcal{I}_C$ (Assumption C.3), estimators based on the efficient influence function are not guaranteed to be at least as efficient as IPW estimators. This is because the identifying formula for $\Psi^a_{\mathcal{I}_C}$ under $\mathcal{I}_C$ (equation D.3) depends on the distribution of R_A, and so estimators based on the efficient influence function may not attain the efficiency bound for $\Psi^a_{\mathcal{I}_C}$ under semiparametric models where missingness models are known or modeled parametrically.

E. Derivation of efficient influence functions

For any parameter Ψ, the efficient influence function in the nonparametric model $\mathcal{M}_{np}$ is defined as the unique mean zero, finite variance random variable $\varphi^{\text{eff}}(O)$ such that

$$\frac{d\Psi(\theta_t)}{dt}\bigg|_{t=0} = E\{\varphi^{\text{eff}}(O)S(O)\}$$

where $d\Psi(\theta_t)/dt|_{t=0}$ is known as the pathwise derivative of parameter Ψ along a parametric submodel θ_t indexed by t, $S(O)$ is the score function of the parametric submodel evaluated at t=0, and O denotes the observed data as before. We now show the efficient influence function under various missingness assumptions. We provide more details of the derivation in the first proof; details in other proofs are omitted to the similar nature of the efficient influence function derivations.

E.1 Efficient influence function under Assumption A.1 ($\mathcal{I}_{joint}$) The efficient influence function for $\Psi^a_{\mathcal{I}_{joint}}$ under given by identifying formula (A.1) under $\mathcal{M}_{np}$ equals:

$$\phi^1_{P_{\mathcal{I}_{joint}}}(O) = \frac{I(A=a,R=1)}{\pi_A\pi_R}(Y-T_1) + \frac{I(R=1)}{\pi_R}(T_1-T_0) + T_0 - \Psi^a_{\mathcal{I}_{joint}}$$

where we let $\pi_A = P(A=a|R=1,L)$, $\pi_R = P(R=1|L_O)$, $T_1 := T_1(L) = E(Y|A=a,R=1,L)$ and $T_0 := T_0(L_O) = E(T_1|R=1,L_O)$.

Proof. Under Assumption A.1, the identifying formula can be written as:

$$\Psi^a_{\mathcal{I}_{joint}} = E[E\{E(Y|L,A=a,R=1)|L_O,R=1\}].$$

Then,

$$\frac{d\Psi^a_{\mathcal{I}_{joint}}(\theta_t)}{dt}\bigg|_{t=0} = E[E\{E(YS(Y|L,A=a,R=1)|L,A=a,R=1)|L_O,R=1\}] + \tag{E.5}$$

$$E\left[E\left\{\underbrace{E(Y|L,A=a,R=1)}_{T_1}S(L_M|L_O,R=1)|L_O,R=1\right\}\right] + \tag{E.6}$$

$$E\left[\underbrace{E\{E(Y|L,A=a,R=1)|L_O,R=1\}}_{T_0}S(L_O)\right] \tag{E.7}$$

where $S(Y|L,A,R)$, $S(L_M|L_O,R)$, and $S(L_O)$ denote the conditional scores of Y, L_M and marginal score of L_O, respectively. Expressions (E.5), (E.6) and (E.7) respectively equal:

$$(\text{E.5}) = E\left\{\frac{I(R=1,A=a)}{P(A=a|L,R=1)P(R=1|L_O)}YS(Y|L,A,R)\right\}$$

$$=E\left\{\frac{I(R{=}1,A{=}a)}{P(A{=}a|L,R{=}1)P(R{=}1|L_O)}S(Y|L,A,R)(Y-E(Y|L,A,R))\right\}$$
$$=E\left\{\frac{I(R{=}1,A{=}a)}{P(A{=}a|L,R{=}1)P(R{=}1|L_O)}(Y-T_1)S(O)\right\}$$

where the first line holds by probability rules; the second line holds by the fact that $E(Y|L,A,R)$ is a function of (L,A,R) and that

$$E\left\{\frac{I(R{=}1,A{=}a)}{P(A{=}a|L,R{=}1)P(R{=}1|L_O)}T_1S(Y|L,A,R)\right\}=0;$$

and the third line holds by the fact that

$$E\left\{\frac{I(R{=}1,A{=}a)}{P(A{=}a|L,R{=}1)P(R{=}1|L_O)}(Y-T_1)S(L,A,R)\right\}=0.$$

Since $I(R{=}1)S(Y|L,A,R)=I(R{=}1)S(Y|L_O,RL_M,RA,R)$ and since $I(R{=}1)S(L,A,R)= I(R{=}1)S(L_O,RL_M,RA,R)$, it then follows that $S(O)=S(Y|L,A,R)+S(L,A,R)$.

$$(\text{E.6})=E\left\{\frac{I(R{=}1)}{P(R{=}1|L_O)}T_1S(L_M|L_O,R)\right\}$$
$$=E\left\{\frac{I(R{=}1)}{P(R{=}1|L_O)}(T_1-E(T_1|R,L_O))S(L_M|L_O,R)\right\}$$
$$=E\left\{\frac{I(R{=}1)}{P(R{=}1|L_O)}(T_1-T_0)S(O)\right\}$$

where the first line holds by probability rules; the second line holds by the fact that $E(T_1|R,L_O)$ is a function of (R,L_O) and that

$$E\left\{\frac{I(R{=}1)}{P(R{=}1|L_O)}E(T_1|R,L_O)S(L_M|L_O,R)\right\}=0;$$

and the third line holds by the facts that

$$E\left\{I(R{=}1)P(R{=}1|L_O)^{-1}(T_1-T_0)S(Y,A|L,R)\right\}=0$$
$$E\left\{I(R{=}1)P(R{=}1|L_O)^{-1}(T_1-T_0)S(L_O,R)\right\}=0.$$

Since $I(R{=}1)S(Y,A|L,R)=I(R{=}1)S(Y,RA|L_O,RL_M,R)$, $I(R{=}1)S(L_M|,L_O,R)=I(R{=}1)S(RL_M|,L_O,R)$, it then follows that $S(O)=S(Y,A|L,R)+S(L_M|L_O,R)+S(L_O,R)$.

$$(\text{E.7})=E\left\{(T_0-\Psi^a_{\mathcal{I}_{joint}})S(L_O)\right\}$$
$$=E\left\{(T_0-\Psi^a_{\mathcal{I}_{joint}})S(O)\right\}$$

where the first equality holds by the fact that $\Psi^a_{\mathcal{I}_{joint}} E\{S(L_O)\}=0$; and the second line follows by the fact that $E\Big\{(T_0 - \Psi^a_{\mathcal{I}_{joint}}) S(Y, RA, RL_M, R|L_O)\Big\}=0$ and $S(O)= S(Y, RA, RL_M, R|L_O) + S(L_O)$.

Hence $\phi^1_{P_{\mathcal{I}_{joint}}}(O)$ satisfies $\frac{d\Psi^a_{\mathcal{I}_{joint}}(\theta_t)}{dt}\Big|_{t=0} = E\{\phi^1_{P_{\mathcal{I}_{joint}}}(O)S(O)\}$. □

E.2 Efficient influence function under Assumption 5 ($\mathcal{I}_A$) The efficient influence function for $\Psi^a_{\mathcal{I}_A}$ given by identifying formula (2) under $\mathcal{M}_{np}$ equals:

$$\phi^1_{P_{\mathcal{I}_A}} = \frac{I(A=a, R_A=1, R_L=1)}{\pi_A \pi_{R_A} \pi_{R_L}}(Y - T_1) + \frac{I(R_L=1)}{\pi_{R_L}}(T_1 - T_0) + T_0 - \Psi^a_{\mathcal{I}_A}$$

where we let $\pi_A = P(A=a|L, R_A=1, R_L=1)$, $\pi_{R_A} = P(R_A=1|L, R_L=1)$, $\pi_{R_L} = P(R_L=1|L_O)$, $T_1 := T_1(L) = E(Y|A=a, L, R_A=1, R_L=1)$ and $T_0 := T_0(L_O) = E(T_1|L_O, R_L=1)$.

Proof. Under Assumption 5, the identifying formula can be written as:

$$\Psi^a_{\mathcal{I}_A} = E[E\{E(Y|L, A=a, R=1)|L_O, R_L=1\}].$$

Then,

$$\frac{d\Psi^a_{\mathcal{I}_A}(\theta_t)}{dt}\Big|_{t=0} = E[E\{E(YS(Y|L, A=a, R=1)|L, A=a, R=1)|L_O, R_L=1\}] + \tag{E.8}$$

$$E\left[E\left\{\underbrace{E(Y|L, A=a, R=1)}_{T_1} S(L_M|L_O, R=1)|L_O, R_L=1\right\}\right] + \tag{E.9}$$

$$E\left[\underbrace{E\{E(Y|L, A=a, R=1)|L_O, R_L=1\}}_{T_0} S(L_O)\right] \tag{E.10}$$

It can be shown that Expressions (E.8), (E.9) and (E.10) respectively equal,

$$E\left\{\frac{I(R=1, A=a)}{P(A=a|L, R=1)P(R_A=1|L, R_L=1)P(R_L=1|L_O)}(Y - T_1)S(O)\right\}$$

$$E\left\{(T_1 - T_0)\frac{I(R_L=1)}{P(R_L=1|L_O)}S(O)\right\}$$

$$E\big\{(T_0 - \Psi^a_{\mathcal{I}_A})S(O)\big\}$$

□

E.3 Efficient influence function under Assumption 7 ($\mathcal{I}_B$) The derivation of the efficient influence function for $\Psi^a_{\mathcal{I}_B}$ given by identifying formula 5 under $\mathcal{M}_{np}$ is given below.

First note that the identifying formula 5 can be written as follows:

$$\Psi^a_{\mathcal{I}_B} = E\left(E\left[\ldots E\left\{\underbrace{E(Y|A=1,R=1,L)}_{\tilde{T}_q:=\tilde{T}_q(L)}|L_O,\bar{L}_{M,q-1},\bar{R}_{Lq}=1_q\right\}\ldots|L_O,R_{L1}=1\right]\right)$$

$$=E\left(E\left[\ldots\underbrace{E\left(\tilde{T}_q|L_O,\bar{L}_{M,q-1},\bar{R}_{Lq}=1_q\right)}_{\tilde{T}_{q-1}:=\tilde{T}_{q-1}(L_{M,q-1},L_O)}\ldots|L_O,R_{L1}=1\right]\right)=\ldots=E\{\underbrace{\tilde{T}_0(L_O)}_{:=\tilde{T}_0}\}$$

Proof.

$$\frac{d\Psi^a_{\mathcal{I}_B}(\theta_t)}{dt}\Big|_{t=0} = E\Big(\ldots E\{YS(Y,L,A=a,R=1)|L,A=a,R=1\}\ldots\Big) \tag{E.11}$$

$$+\sum_{k=1}^{q} E\Big(\ldots E\Big\{\tilde{T}_k S(\bar{L}_{Mk},L_O\bar{R}_{Lk}=1_k)|\bar{L}_{M,k-1},L_O\bar{R}_{Lk}=1_k)\Big\}\ldots\Big) \tag{E.12}$$

$$+E\Big\{\tilde{T}_0 S(L_O)\Big\} \tag{E.13}$$

After some algebra, it can be shown that the right-hand-side of Equation (E.11) is equivalent to:

$$E\Big(\ldots E\{YS(Y,L,A=a,R=1)|L,A=a,R=1\}\ldots\Big)$$
$$=E\left[\frac{I(A=a,R_A=1,\bar{R}_{Lq}=1_q)}{\pi_A(L)\pi_{R_A}(L)\prod_{j=1}^{q}\pi_{R_{Lj}}(\bar{L}_{M,j-1},L_O)}(Y-\tilde{T}_q)S(O)\right]$$

Each kth element in Expression (E.12) can be shown to be equivalent to:

$$E\Big(\ldots E\Big\{\tilde{T}_k S(\bar{L}_{Mk},L_O\bar{R}_{Lk}=1_k)|\bar{L}_{M,k-1},L_O\bar{R}_{Lk}=1_k)\Big\}\ldots\Big)$$
$$=E\left[\frac{I(\bar{R}_{Lk}=1_k)}{\prod_{j=1}^{k}\pi_{R_{Lj}}(\bar{L}_{M,j-1},L_O)}\{\tilde{T}_k(\bar{L}_{Mk},L_O)-\tilde{T}_{k-1}(\bar{L}_{M,k-1},L_O)\}S(O)\right]$$

Finally, Expression (E.13) is equivalent to:

$$E\Big\{\tilde{T}_0 S(L_O)\Big\} = E\Big\{(\tilde{T}_0-\Psi^a_{\mathcal{I}_B})S(O)\Big\}$$

We obtain our desired result after combining (E.11), (E.12) and (E.13):

$$\phi^1_{P_{\mathcal{I}_\mathbf{B}}} = \frac{I(A=a,R_A=1,\bar{R}_{Lq}=1_q)}{\pi_A(L)\pi_{R_A}(L)\prod_{j=1}^{q}\pi_{R_{Lj}}(\bar{L}_{M,j-1},L_O)}\{Y-\tilde{T}_q(L)\}+$$

$$\sum_{k=1}^{q}\frac{I(\bar{R}_{Lk}=1_k)}{\prod_{j=1}^{k}\pi_{R_{Lj}}(\bar{L}_{M,j-1},L_O)}\{\tilde{T}_k(\bar{L}_{Mk},L_O)-\tilde{T}_{k-1}(\bar{L}_{M,k-1},L_O)\}+\tilde{T}_0(L_O)-\Psi^a_{\mathcal{I}_B}$$

□

E.4 Efficient influence function under Assumption C.3 ($\mathcal{I}_C$) The efficient influence function for $\Psi^a_{\mathcal{I}_C}$ given by identifying formula (2) under $\mathcal{M}_{np}$ equals:

$$\phi^1_{P_{\mathcal{I}_C}}=\frac{I(A=a,R_A=1,R_L=1)}{\pi_A\pi_{R_A}}\left\{\sum_{r_A}\frac{P(R_A=r_A|L,R_L=1)}{P(R_L=1|L_O,R_A=r_A)}\right\}(Y-T_1)$$
$$+\frac{I(R_L=1)}{P(R_L=1|L_O,R_A)}(T_1-\widetilde{T}_0)+\widetilde{T}_0-\Psi^a_{\mathcal{I}_C}$$

where $\pi_A=P(A=a|L,R_A=1,R_L=1)$, $\pi_{R_A}=P(R_A=1|L,R_L=1)$, $T_1:=T_1(L)=E(Y|A=a,L,R_A=1,R_L=1)$, and $\widetilde{T}_0:=\widetilde{T}_0(L_O)=E(T_1|L_O,R_A,R_L=1)$.

Proof. Under Assumption C.3, the identifying formula can be written as:

$$\Psi^a_{\mathcal{I}_C}=E[E\{E(Y|L,A=a,R=1)|R_A,L_O,R=1\}].$$

Then,

$$\left.\frac{d\Psi^a_{\mathcal{I}_C}(\theta_t)}{dt}\right|_{t=0}=E[E\{E(YS(Y|L,A=a,R=1)|L,A=a,R=1)|L_O,R=1,R_A\}]+ \tag{E.14}$$

$$E\left[E\left\{\underbrace{E(Y|L,A=a,R=1)}_{T_1}S(L_M|L_O,R=1,Y)|L_O,R=1,R_A\right\}\right]+ \tag{E.15}$$

$$E\left[\underbrace{E\{E(Y|L,A=a,R=1)|L_O,R=1,R_A\}}_{\tilde{T}_0}S(L_O,R_A)\right] \tag{E.16}$$

It can be shown that Expressions (E.14), (E.15) and (E.16) respectively equal,

$$E\left\{\frac{I(A=a,R=1)}{P(A=a|R=1,L)P(R_A=1|L,R_L=1)}\left(\sum_{r_A}\frac{P(R_A=r_A|L,R_L=1)}{P(R_L=1|L_O,R_A=r_A)}\right)(Y-T_1)S(O)\right\}$$

$$E\left\{(T_1-\tilde{T}_0)\frac{I(R_L=1)}{P(R_L=1|L_O,R_A)}S(O)\right\}$$

$$E\left\{(\tilde{T}_0-\Psi^a_{\mathcal{I}_C})S(O)\right\}$$

□

E.5 Efficient influence function under Assumption 3 (MAR) The efficient influence function for Ψ^a_{MAR} given by identifying formula (1) under $\mathcal{M}_{np}$ is given by:

$$\begin{aligned}\phi^1_{P_{MAR}} =& E\left\{\frac{\beta(L)}{\gamma(L)}|L_O,Y,R=1\right\}+\frac{R}{P(R=1|L_O,Y)}\left[\frac{\beta(L)}{\gamma(L)}-E\left\{\frac{\beta(L)}{\gamma(L)}|L_O,Y,R=1\right\}\right]-\Psi^a_{MAR}\\ &+E\left[\frac{I(A=a)\Omega(L)}{\gamma(L)}\left(Y-\frac{\beta(L)}{\gamma(L)}\right)|L_O,Y,R=1\right]\\ &+\frac{R}{P(R=1|L_O,Y)}\left[\frac{I(A=a)\Omega(L)}{\gamma(L)}\left(Y-\frac{\beta(L)}{\gamma(L)}\right)-E\left\{\frac{I(A=a)\Omega(L)}{\gamma(L)}\left(Y-\frac{\beta(L)}{\gamma(L)}\right)|L_O,Y,R=1\right\}\right]\end{aligned}$$

where $\beta(L)=\sum_y yp(L_M,a|L_O,y,R=1)p(y|L_O)$ and $\gamma(L)=\sum_{y'}p(L_M,a|L_O,y',R=1)p(y'|L_O)$, and $\Omega(L)=\sum_y p(L_M|y,L_O,R=1)p(y|L_O)$. A consistent estimator of Ψ^a_{MAR} can then be derived based on this efficient influence function. However, this would be challenging in this case because it requires specifying the joint distribution of L_M and A, and may require numerical integration if Y is a continuous variable.

Proof.

$$\frac{d\Psi^a_{MAR}(\theta_t)}{dt}\Big|_{t=0}=\underbrace{\frac{d}{dt}E_{P_t}\left[E_P\left\{\frac{\beta(L)R}{\gamma(L)\pi}|L\right\}\right]}_{(A)}+\underbrace{E_P\left\{\frac{\frac{d}{dt}\beta_t(L)}{\gamma(L)}-\frac{\beta(L)\frac{d}{dt}\gamma_t(L)}{\gamma(L)^2}\right\}}_{(B)}-\underbrace{E_P\left\{\frac{\beta(L)R}{\gamma(L)\frac{d\pi_t}{dt}}\right\}}_{(C)}$$

where notational simplicity, we let $\pi=P(R=1|L_O,Y)$ and $\pi_t=P_t(R=1|L_O,Y)$. We look at each (A), (B), and (C) terms separately. After some algebra, it can be shown that term (A) equals:

$$(\text{A})=E\left[\left\{\frac{R}{\pi}\frac{\beta(L)}{\gamma(L)}-\Psi^a_{MAR}\right\}S(O)\right],$$

term (B) equals:

$$\begin{aligned}(\text{B})=&E\left[\frac{RY}{\pi}\left\{\frac{I(A=a)\Omega(L)}{\gamma(L)}-E\left(\frac{I(A=a)\Omega(L)}{\gamma(L)}|L_O,Y,R\right)\right\}S(O)\right]+\\ &E\left\{\frac{I(A=a)R\Omega(L)}{\gamma(L)\pi}YS(Y|L_O)\right\}-\\ &E\left[\frac{R}{\pi}\left\{\frac{I(A=a)\Omega(L)\beta(L)}{\gamma(L)^2}-E\left(\frac{I(A=a)\Omega(L)\beta(L)}{\gamma(L)^2}|L_O,Y,R\right)\right\}S(O)\right]-\\ &E\left\{\frac{I(A=a)R\Omega(L)\beta(L)}{\gamma(L)^2\pi}YS(Y|L_O)\right\},\end{aligned}$$

This reduces to:

$$(\text{B})=E\left[\frac{RY}{\pi}\left\{\frac{I(A=a)\Omega(L)}{\gamma(L)}-E\left(\frac{I(A=a)\Omega(L)}{\gamma(L)}|L_O,Y,R\right)\right\}S(O)\right]-$$

$$E\left[\frac{R}{\pi}\left\{\frac{I(A{=}a)\Omega(L)\beta(L)}{\gamma(L)^2}-E\left(\frac{I(A{=}a)\Omega(L)\beta(L)}{\gamma(L)^2}|L_O,Y,R\right)\right\}S(O)\right]+$$
$$E\left[E\left\{\frac{I(A{=}a)\Omega(L)}{\gamma(L)}\left(Y-\frac{\beta(L)}{\gamma(L)}\right)|L_O,Y,R{=}1\right\}S(O)\right]$$

Finally, it can be shown that term (C) equals:

$$\text{(C)}{=}E\left[E\left\{\frac{\beta(L)}{\gamma(L)}|L_O,Y,R{=}1\right\}(R-\pi)\frac{1}{\pi}S(O)\right]$$

Combining the results for (A), (B) and (C) allows us to see the final expression for the efficient influence function of Ψ^a_{MAR}.

□

F. Consistency of multiple imputation under MAR

In the absence of missing data, the identifying formula for Ψ^a_{MAR} is given by:

$$\Psi^a_{MAR}{=}\sum_l E(Y|A{=}a,L{=}l)p(l).$$

When there are missing data in A and L_M, we can impute missing values when $R{=}0$ under the simultaneous missingness mechanism using e.g., multiple imputation using chained equations.

Let M be the number of imputations. For $m{=}1,\ldots,M$, we impute missing A and L_M values via off-the-shelf standard multiple imputation algorithms such as `mice`. The imputation model should include L_O, Y and possibly their interactions. In the mth imputed complete dataset, we can estimate $E(Y^a)$ using TMLE (or any other complete-data estimator) and obtain a complete-data estimate $\hat{E}_m(Y^a)$. We then obtain the MI estimator by averaging over the M estimates: $\widehat{\Psi}^a_{MAR,MI}{=}M^{-1}\sum_{m=1}^M\hat{E}_m(Y^a)$. Standard errors can be obtained following Rubin's combining rules (Rubin, 2004), provided that the imputation models and nuisance function models in the complete-case TMLE estimator are compatible, and that there is no model misspecification (Hughes *and others*, 2016); otherwise the bootstrap can be used.

Suppose that we use a parametric g-formula estimator such as iterative conditional expectation (ICE) that first aims to estimate $E(Y|A{=}a,L)$ for each observation, and then takes an empirical average of the estimated values of $E(Y|A{=}a,L)$ to obtain an estimate of $E(Y^a)$. We will aim to show that the estimating equations for estimating $E(Y|A{=}a,L)$ is unbiased under multiple imputation. Suppressing the iteration of the imputed data set, we can estimate $E(Y|A{=}a,L)$ by solving for β in the following set of estimating equations:

$$0{=}\mathbb{P}_n\Big[I(A{=}a)R\phi(L_M)\{Y{-}T_1(L_O,L_M;\beta)\}{+}I(A(\hat{\theta}){=}a)(1{-}R)\phi(L_M(\hat{\theta}))\{Y{-}T_1(L_O,L_M(\hat{\theta});\beta)\}\Big]$$

where $\phi(L_M)$ is a known function of L_M; $T_1(L_O,L_M;\beta)$ is a model for $E(Y|A{=}a,L)$ parameterized by β; $A(\hat{\theta})$ and $L_M(\hat{\theta})$ denote random draws of A and L_M (respectively) from an estimated probability distribution of an imputation model given by $f(A,L_M|L_O,Y;\hat{\theta})$ for $f(A,L_M|L_O,Y)$ where $\hat{\theta}$ is an estimated value (e.g., maximum likelihood estimate) for θ. We argue from the perspective of improper imputation, but arguments can be applied to proper imputation where $A(\hat{\theta})$ and $L_M(\hat{\theta})$ are drawn from the posterior predictive distribution $f(A,L_M|L_O,Y;\hat{\theta})$ for $f(A,L_M|L_O,Y)$ where $\hat{\theta}$ is a random draw from an observed-data posterior distribution for θ. Paik (1997) and Wang and Robins (1998) have previously shown that proper and improper imputation are equivalent when $n{\to}\infty$ and $M{\to}\infty$.

It suffices to show that the set of estimating equations has mean zero when $n{\to}\infty$. Suppose that β^* is the limiting value of $\hat{\beta}$ (i.e., the value of β that solves the aforementioned set of estimating equations). If the model for $E(Y|A{=}a,L)$ is correctly specified, then $\beta^*{=}\beta$. Moreover, let θ^* denote the limiting value of $\hat{\theta}$. When the imputation model is correctly specified, $f(A,L_M|L_O,Y;\theta^*){=}f(A,L_M|L_O,Y)$. Thus, it follows that:

$$E\left[\underbrace{I(A{=}a)R\phi(L_M)\{Y-T_1(L_O,L_M;\beta^*)\}}_{(A)}+\underbrace{I(A(\theta^*){=}a)(1-R)\phi(L_M(\theta^*))\{Y-T_1(L_O,L_M(\theta^*);\beta^*)\}}_{(B)}\right]$$

where the expectation of term (B) equals:

$$\begin{aligned}(B)&=E\left[\sum_{l_M}(1-R)P(A{=}a|L_O,l_M,Y;\theta^*)\phi(l_M)\{Y-E(Y|A{=}a,l_M,L_O;\beta^*)\}f(l_M|L_O,Y,R{=}0;\theta^*)\right]\\&=E\left[\sum_{l_M}P(R{=}0|L_O,Y)P(A{=}a|L_O,l_M,Y;\theta^*)\phi(l_M)\{Y-E(Y|A{=}a,l_M,L_O;\beta^*)\}f(l_M|L_O,Y;\theta^*)\right]\end{aligned}$$

where the last line follows from MAR. Under correctly specified imputation models, this expectation becomes:

$$(B)=E\left[\sum_{l_M}P(R{=}0|L_O,Y)P(A{=}a|L_O,l_M,Y)\phi(l_M)\{Y-E(Y|A{=}a,l_M,L_O;\beta^*)\}f(l_M|L_O,Y)\right]$$

Moreover, the expectation of term (A) equals:

$$(A)=E\left[\sum_{l_M}P(R{=}1|L_O,Y)P(A{=}a|L_O,l_M,Y)\phi(l_M)\{Y-E(Y|A{=}a,l_M,L_O;\beta^*)\}f(l_M|L_O,Y)\right]$$

Thus, it follows that the sum of expectations of (A) and (B) equals:

$$\begin{aligned}&E\left[\sum_{l_M}P(A{=}a|L_O,l_M,Y)\phi(l_M)\{Y-E(Y|A{=}a,l_M,L_O;\beta^*)\}f(l_M|L_O,Y)\right]\\&=E[I(A{=}a)\phi(L_M)\{Y-E(Y|A{=}a,L;\beta^*)\}]\end{aligned}$$

which equals 0 under the correct model for $E(Y|A{=}a,L)$ (whereby $\beta^*{=}\beta$).

G. ICE and IPW Estimators

G.1 ICE Estimator Under Assumption A.1 $\mathcal{I}_{Joint}$ Straightforward non-iterative conditional estimator (NICE) parametric g-formula computation based on formula (A.1; Wen *and others*, 2021) would require a model for $p(l_M|l_O,R{=}1)$, which is particularly challenging when L_M is high dimensional. Instead, we re-write the identifying formula (A.1) under Assumption A.1 as:

$$\begin{aligned}\Psi^a_{\mathcal{I}_{joint}} &= \sum_{l_O}\sum_{l_M} E(Y|A{=}a,R{=}1,L_M{=}l_M,L_O{=}l_O)p(l_M|l_O,R{=}1)p(l_O)\\ &= \sum_{l_O}\sum_{l_M} T_1 p(l_M|l_O,R{=}1)p(l_O)\\ &= \sum_{l_O} E[T_1|L_O{=}l_O,R{=}1]p(l_O)\\ &= \sum_{l_O} T_0 p(l_O)\end{aligned}$$

where $T_1{=}E(Y|A{=}a,R{=}1,L)$ and $T_0{=}E(T_1|R{=}1,L_O)$. Using this formulation, we propose an ICE, $\hat{\Psi}^a_{ICE-Joint}$, computed as follows:

Algorithm 3 Algorithm for ICE-Joint under $\mathcal{I}_{joint}$

1: Among subjects with $R{=}1$, fit a regression model $\eta_1(A,L;\kappa_1){=}g^{-1}([A,L]'\kappa_1)$ by regressing Y on A and L.
2: Among those with $R{=}1$, predict $\hat{T}_1$ (estimate of T_1) from $\eta_1(A{=}a,L;\hat{\kappa}_1)$.
3: Among those with $R{=}1$, fit a regression model $\eta_0(L_O;\kappa_0){=}g^{-1}(L_O'\kappa_0)$ by regressing $\hat{T}_1$ on L_O.
4: Predict $\hat{T}_0$ (estimate of T_0) from $\eta_0(L_O;\hat{\kappa}_0)$ for all observations.
5: Calculate the ICE estimator $\hat{\Psi}^a_{ICE-Joint}{=}\mathbb{P}_n(\hat{T}_0)$.

G.2 IPW Estimator Under Assumption A.1 $\mathcal{I}_{Joint}$ We re-write the identifying formula (A.1) under Assumption A.1 as:

$$\Psi^a_{\mathcal{I}_{joint}} = E\left\{\frac{I(A{=}a,R{=}1)}{P(A{=}a|L,R{=}1)P(R{=}1|L_O)}Y\right\}$$

Using the inverse probability weighted (IPW) representation of the identifying formula, we can solve for an IPW estimator by solving for $\Psi^a_{IPW-Joint}$ in the following set of estimating equations:

$$\mathbb{P}_n\left\{\frac{I(A{=}a,R{=}1)}{\hat{P}(A{=}a|L,R{=}1)\hat{P}(R{=}1|L_O)}(Y-\Psi^a_{IPW-Joint})\right\}$$

where $\hat{P}(A{=}a|,L,R{=}1){:=}P(A{=}a|,L,R{=}1;\hat{\alpha}_A)$ and $\hat{P}(R{=}1|L_O){:=}P(R{=}1|L_O;\hat{\alpha}_{R,\mathcal{I}_A})$ denote estimates of $P(A{=}a|L,R{=}1)$ and $P(R{=}1|L_O)$, which are parameterized by α_A and $\alpha_{R,\mathcal{I}_A}$, respectively.

G.3 ICE Estimator Under Assumption 5 $\mathcal{I}_A$ We re-write the identifying formula (2) under Assumption 5 as:

$$\begin{aligned}\Psi^a_{\mathcal{I}_A} &= \sum_{l_O}\sum_{l_M} E(Y|A{=}a,R_A{=}1,R_L{=}1,L_O{=}l_O,L_M{=}l_M)p(l_M|l_O,R_L{=}1)p(l_O)\\ &= \sum_{l_O}\sum_{l_M} T_1 p(l_M|l_O,R_L{=}1)p(l_O)\\ &= \sum_{l_O} E[T_1|L_O{=}l_O,R_L{=}1]p(l_O)\\ &= \sum_{l_O} T_0 p(l_O)\end{aligned}$$

where $T_1{=}E(Y|A{=}a,L_M,L_O,R_A{=}1,R_L{=}1)$ and $T_0{=}E(T_1|L_O,R_L{=}1)$. Using this formulation, we propose an ICE estimator $\hat{\Psi}^a_{ICE-A}$ computed as follows:

Algorithm 4 Algorithm for ICE-A under $\mathcal{I}_A$

1: Among subjects with $R_A{=}1$ and $R_L{=}1$, fit a regression model $\eta_1(A,L;\kappa_1){=}g^{-1}([A,L]'\kappa_1)$ by regressing Y on A and L.
2: Among those with $R_L{=}1$, predict $\hat{T}_1$ (estimate of T_1) from $\eta_1(A{=}a,L;\hat{\kappa}_1)$.
3: Among those with $R_L{=}1$, fit a regression model $\eta_0(L_O;\kappa_0){=}g^{-1}(L_O'\kappa_0)$ by regressing $\hat{T}_1$ on L_O.
4: Predict $\hat{T}_0$ (estimate of T_0) from $\eta_0(L_O;\hat{\kappa}_0)$ for all observations.
5: Calculate the ICE estimator $\hat{\Psi}^a_{ICE-A}{=}\mathbb{P}_n(\hat{T}_0)$.

G.4 IPW Estimator Under Assumption 5 $\mathcal{I}_A$ We re-write the identifying formula (2) under Assumption 5 as:

$$\Psi^a_{\mathcal{I}_A} = E\left\{\frac{I(A{=}a,R{=}1)}{P(A{=}a|L,R{=}1)P(R_A{=}1|L,R_L{=}1)P(R_L{=}1|L_O)}Y\right\}$$

Using the inverse probability weighted (IPW) representation of the identifying formula, we can solve for an IPW estimator by solving for Ψ^a_{IPW-A} in the following set of estimating equations:

$$\mathbb{P}_n\left\{\frac{I(A{=}a,R{=}1)}{\hat{P}(A{=}a|L,R{=}1)\hat{P}(R_A{=}1|L,R_L{=}1)\hat{P}(R_L{=}1|L_O)}(Y-\Psi^a_{IPW-A})\right\}$$

where $\hat{P}(A{=}a|,L,R{=}1){:=}P(A{=}a|,L,R{=}1;\hat{\alpha}_A)$, $\hat{P}(R_A{=}1|L,R_L{=}1){:=}P(R_A{=}1|L,R_L{=}1;\hat{\alpha}_{R_A,\mathcal{I}_A})$ and $\hat{P}(R_L{=}1|L_O){:=}P(R_L{=}1|L_O;\hat{\alpha}_{R_L,\mathcal{I}_A})$ denote estimates of $P(A{=}a|L,R{=}1)$,

$P(R_A{=}1|L,R_L{=}1)$ and $P(R_L{=}1|L_O)$, which are parameterized by α_A, $\alpha_{R_A,\mathcal{I}_A}$, and $\alpha_{R_L,\mathcal{I}_A}$ respectively.

G.5 ICE Estimator Under Assumption 7 $\mathcal{I}_B$ We propose an ICE estimator $\hat{\Psi}^a_{ICE-B}$ computed as follows:

Algorithm 5 Algorithm for ICE-B under $\mathcal{I}_B$

1: Among those with $R_A{=}1$ and $\bar{R}_{Lq}{=}1_q$, fit a regression model $\zeta(A,L;\kappa){=}g^{-1}([A,L]'\kappa)$ by regressing Y on A and L.
2: Among those with $\bar{R}_{Lq}{=}1_q$, predict $\tilde{T}_q(L)$ using $\hat{\tilde{T}}_q(L){=}g^{-1}[g\{\zeta(A{=}a,L;\hat{\kappa})\}]$.
3: Recursively from $k{=}q,...,1$:
   (A) Among those with $\bar{R}_{Lk}{=}1_k$, fit a regression model $\eta_{k-1}(\bar{L}_{M,k-1},L_O;\omega_{k-1}){=}g^{-1}([\bar{L}_{M,k-1},L_O]'\omega_{k-1})$ by regressing $\hat{\tilde{T}}_k(\bar{L}_{Mk},L_O)$ on $\bar{L}_{M,k-1}$ and L_O.
   (B) Among those with $\bar{R}_{L,k-1}{=}1_{k-1}$, Predict $\tilde{T}_{k-1}(\bar{L}_{M,k-1},L_O)$ using $\hat{\tilde{T}}_{k-1}(\bar{L}_{M,k-1},L_O){=}g^{-1}\left[g\{\eta_{k-1}(\bar{L}_{M,k-1},L_O;\hat{\omega}_{k-1})\}\right]$.
4: Calculate the ICE estimator $\widehat{\Psi}_{\mathbf{ICE},\mathcal{I}_\mathbf{B}}{=}\mathbb{P}_n\{\hat{\tilde{T}}_0(L_O)\}$.

G.6 IPW Estimator Under Assumption 7 $\mathcal{I}_B$ Using the inverse probability weighted (IPW) representation of the identifying formula, we can solve for an IPW estimator by solving for Ψ^a_{IPW-B} in the following set of estimating equations:

$$\mathbb{P}_n\left\{\frac{I(A{=}a,R{=}1)}{\hat{P}(A{=}a|L,R{=}1)\hat{P}(R_A{=}1|L,\bar{R}_{Lq}{=}1_q)\prod_{k=1}^q\hat{P}(R_{Lk}{=}1|\bar{R}_{L,k-1}{=}1_{k-1},\bar{L}_{M,k-1},L_O)}(Y-\Psi^a_{IPW-B})\right\}$$

where $\hat{P}(A{=}a|,L,R{=}1){:=}P(A{=}a|,L,R{=}1;\hat{\alpha}_A)$, $\hat{P}(R_A{=}1|L,R_L{=}1){:=}P(R_A{=}1|L,R_L{=}1;\hat{\alpha}_{R_A,\mathcal{I}_B})$ and $\hat{P}(R_{Lk}{=}1|\bar{R}_{L,k-1}{=}1_{k-1},\bar{L}_{M,k-1},L_O;\hat{\alpha}_{R_L,\mathcal{I}_B})$ denote estimates of $P(A{=}a|L,R{=}1)$, $P(R_A{=}1|L,R_L{=}1)$ and $P(R_{Lk}{=}1|\bar{R}_{L,k-1}{=}1_{k-1},\bar{L}_{M,k-1},L_O)$, which are parameterized by α_A, $\alpha_{R_A,\mathcal{I}_B}$, and $\alpha_{R_L,\mathcal{I}_B}$ respectively.

G.7 ICE Estimator Under Assumption C.3 $\mathcal{I}_C$ We re-write the identifying formula (D.3) under Assumption C.3 as:

$$\begin{aligned}\Psi^a_{\mathcal{I}_C}&=\sum_{l_O}\sum_{l_M}E(Y|A{=}a,R_A{=}1,R_L{=}1,L_O{=}l_O,L_M{=}l_M)\sum_{r_A}p(l_M|r_A,l_O,R_L{=}1)p(r_A|l_O)p(l_O)\\&=\sum_{l_O}\sum_{l_M}T_1\sum_{r_A}p(l_M|r_A,l_O,R_L{=}1)p(r_A|l_O)p(l_O)\\&=\sum_{l_O}\sum_{r_A}\sum_{l_M}T_1p(l_M|r_A,l_O,R_L{=}1)p(r_A|l_O)p(l_O)\\&=\sum_{l_O}\sum_{r_A}E[T_1|R_A{=}r_A,L_O{=}l_O,R_L{=}1]p(r_A|l_O)p(l_O)\end{aligned}$$

$$=\sum_{l_O}\sum_{r_A}\widetilde{T}_0 p(r_A|l_O)p(l_O)$$

where $T_1{=}E(Y|A{=}a,L_M,L_O,R_A{=}1,R_L{=}1)$, and $\widetilde{T}_0{=}E(T_1|L_O,R_A,R_L{=}1)$. Using this formulation, we propose an ICE estimator $\hat{\Psi}^a_{ICE-C}$ computed as follows:

Algorithm 6 Algorithm for ICE-C under $\mathcal{I}_C$

1: Among subjects with $R_A{=}1$ and $R_L{=}1$, fit a regression model $\eta_1(A,L;\kappa_1){=}g^{-1}([A,L]'\kappa_1)$ by regressing Y on A and L.
2: Among those with $R_L{=}1$, predict $\hat{T}_1$ (estimate of T_1) from $\eta_1(A{=}a,L;\hat{\kappa}_1)$.
3: Among those with $R_L{=}1$, fit a regression model $\eta_0(L_O,R_A;\kappa_0){=}g^{-1}([L_O,R_A]'\kappa_0)$ by regressing $\hat{T}_1$ on L_O and R_A.
4: Predict $\widehat{\widetilde{T}}_0$ from $\eta_0(L_O,R_A;\hat{\kappa}_0)$ for all observations.
5: Calculate the ICE estimator $\hat{\Psi}^a_{ICE-C}{=}\mathbb{P}_n(\widehat{\widetilde{T}}_0)$.

G.8 IPW Estimator Under Assumption C.3 $\mathcal{I}_C$ We re-write the identifying formula (D.3) under Assumption C.3 as:

$$\Psi^a_{\mathcal{I}_C}{=}E\left\{\frac{I(A{=}a,R{=}1)}{P(A{=}a|L,R{=}1)P(R_A{=}1|L,R_L{=}1)}\left\{\sum_{r_A}\frac{P(R_A{=}r_A|L,R_L{=}1)}{P(R_L{=}1|L_O,R_A{=}r_A)}\right\}Y\right\}$$

Using the inverse probability weighted (IPW) representation of the identifying formula, we can solve for an IPW estimator by solving for Ψ^a_{IPW-C} in the following set of estimating equations:

$$\mathbb{P}_n\left\{\frac{I(A{=}a,R{=}1)}{\hat{P}(A{=}a|L,R{=}1)\hat{P}(R_A{=}1|L,R_L{=}1)}\left\{\sum_{r_A}\frac{\hat{P}(R_A{=}r_A|L,R_L{=}1)}{\hat{P}(R_L{=}1|L_O,R_A{=}r_A)}\right\}(Y-\Psi^a_{IPW-C})\right\}$$

where $\hat{P}(A{=}a|,L,R{=}1){:=}P(A{=}a|,L,R{=}1;\hat{\alpha}_A)$, $\hat{P}(R_A{=}1|L,R_L{=}1){:=}P(R_A{=}1|L,R_L{=}1;\hat{\alpha}_{R_A,\mathcal{I}_C})$ and $\hat{P}(R_L{=}1|L_O,R_A){:=}P(R_L{=}1|L_O,R_A;\hat{\alpha}_{R_L,\mathcal{I}_C})$ denote estimates of $P(A{=}a|L,R{=}1)$, $P(R_A{=}1|L,R_L{=}1)$ and $P(R_L{=}1|L_O,R_A)$, which are parameterized by α_A, $\alpha_{R_A,\mathcal{I}_C}$, and $\alpha_{R_L,\mathcal{I}_C}$ respectively.

H. Asymptotic properties of TMLE

TMLE-Joint

Consider the following conditions:

C1. $E\left[\phi^1_{\mathbf{P}_{\mathcal{I}_{\mathbf{joint}}}}(O)^2\right]<\infty$

C2. $\phi^1_{\mathbf{P}_{\mathcal{I}_{\mathbf{joint}}}}(O)$ and $\phi^1_{\hat{\mathbf{P}}_{\mathcal{I}_{\mathbf{joint}}}}(O)$ belong to a Donsker family.

C3. $\left\|\phi^1_{\hat{\mathbf{P}}_{\mathcal{I}_{\mathbf{joint}}}}(O)-\phi^1_{\mathbf{P}_{\mathcal{I}_{\mathbf{joint}}}}(O)\right\|_2^2 \xrightarrow{p} 0$

where $\phi^1_{\hat{\mathbf{P}}_{\mathcal{I}_{\mathbf{joint}}}}(O)$ denotes an estimator of $\phi^1_{\mathbf{P}_{\mathcal{I}_{\mathbf{joint}}}}(O)$ where all nuisance functions estimators are exactly the same as those in the TMLE-Joint estimator. It is not hard to show that $\mathbb{P}_n\{\phi^1_{\hat{\mathbf{P}}_{\mathcal{I}_{\mathbf{joint}}}}(O)\}=0$ by construction.

Theorem H.1 (Weak convergence of TMLE-Joint)**.** *Suppose that the conditions C1–C3 hold, and further suppose that the following condition also holds:*

$$\left\|\hat{T}_1(L)-T_1(L)\right\|_2 \|\hat{\pi}_A(L)-\pi_A(L)\|_2+\left\|\hat{T}_0(L_O)-T_0(L_O)\right\|_2 \|\hat{\pi}_R(L_O)-\pi_R(L_O)\|_2=o_p(n^{-1/2}).$$

Then,

$$\sqrt{n}(\widehat{\Psi}^a_{\mathbf{TMLE},\mathcal{I}_\mathbf{A}}-\Psi^a_{\mathcal{I}_{joint}})\rightsquigarrow N(0,\sigma^2), \quad \sigma^2=Var(\phi^1_{P_{\mathcal{I}_{\mathbf{joint}}}}).$$

The variance of TMLE-Joint can be estimated empirically using the sandwich variance estimator or via bootstrap.

Proof. For notational brevity, we suppress O in the notations below and let $\hat{\Psi}^a$ (Ψ^a) denote our TMLE-Joint estimator (functional under $\mathcal{I}_{Joint}$, respectively). An expansion of our estimator gives us:

$$\begin{aligned}\sqrt{n}(\hat{\Psi}^a-\Psi^a)&=\sqrt{n}\left[\mathbb{P}_n(\phi^1_{\hat{\mathbf{P}}})-P(\phi^1_{\hat{\mathbf{P}}})\right]+\sqrt{n}\left[\hat{\Psi}^a+P(\phi^1_{\hat{\mathbf{P}}})-\Psi^a\right]\\&=\mathbb{G}_n(\phi^1_{\mathbf{P}})+\mathbb{G}_n[\phi^1_{\hat{\mathbf{P}}}-\phi^1_{\mathbf{P}}]+\sqrt{n}\left[\hat{\Psi}+P(\phi^1_{\hat{\mathbf{P}}})-\Psi^a\right]\\&=\underbrace{\mathbb{G}_n(\phi^1_{\mathbf{P}})}_{\text{Term 1}}+\underbrace{\mathbb{G}_n[\phi^1_{\hat{\mathbf{P}}}-\phi^1_{\mathbf{P}}]}_{\text{Term 2}}+\sqrt{n}\left[\underbrace{\hat{\Psi}^a+P(\phi^1_{\hat{\mathbf{P}}})-\Psi^a}_{\text{Rem}}\right]\end{aligned}$$

where $\mathbb{G}_n[X]=\sqrt{n}(\mathbb{P}_n-P)(X)$ for any X. Term 1 is a centered sample average which converges to a mean zero Normal distribution by the Central Limit Theorem and by condition C1. Term 2 is known as an empirical process term, which can be shown to be $o_p(1)$ if we assume conditions C2–C3 (e.g., this could be satisfied if the nuisance functions and their

corresponding estimators are not too complex/belong to Donsker class; and that $\phi^1_{\hat{\mathbf{P}}_{\mathcal{I}_{\mathbf{joint}}}}(O)$ converges to $\phi^1_{\mathbf{P}_{\mathcal{I}_{\mathbf{joint}}}}(O)$ in $L_2(P)$). Alternatively, one can use sample splitting and cross fitting to overcome issues with overfitting (Chernozhukov *and others*, 2018).

The third term ("Rem") is a remainder term, which can be further expanded upon. By an application of Cauchy-Schwartz, it is not difficult to show that

$$\hat{\Psi}^a+P(\phi^1_{\hat{\mathbf{P}}})-\Psi^a \lesssim \left\|\hat{T}_1(L)-T_1(L)\right\|_2 \|\hat{\pi}_A(L)-\pi_A(L)\|_2+\left\|\hat{T}_0(L_O)-T_0(L_O)\right\|_2 \|\hat{\pi}_R(L_O)-\pi_R(L_O)\|_2$$

Thus in order for TMLE-Joint to converge asymptotically to a Normal distribution, it is necessary that $\sqrt{n}\{\hat{\Psi}^a+P(\phi^1_{\hat{\mathbf{P}}})-\Psi^a\}=o_p(1)$. □

TMLE-A

Theorem H.2 (Weak convergence of TMLE-A)**.** *Suppose that the conditions C1–C3 hold (where $\mathcal{I}_{joint}$ is replaced with $\mathcal{I}_A$), and further suppose that the following condition also holds:*

$$\left\|\hat{T}_1(L)-T_1(L)\right\|_2 \|\hat{\pi}_A(L)\hat{\pi}_{R_A}(L)-\pi_A(L)\pi_{R_A}(L)\|_2+ \left\|\hat{T}_0(L_O)-T_0(L_O)\right\|_2 \|\hat{\pi}_{R_L}(L_O)-\pi_{R_L}(L_O)\|_2=o_p(n^{-1/2}).$$

Then, $\sqrt{n}(\widehat{\Psi}^a_{\mathbf{TMLE},\mathcal{I}_\mathbf{A}}-\Psi^a_{\mathcal{I}_A})\rightsquigarrow N(0,\sigma^2),\quad \sigma^2=Var(\phi^1_{P_{\mathcal{I}_\mathbf{A}}})$.

TMLE-B

Theorem H.3 (Weak convergence of TMLE-B)**.** *Suppose that the conditions C1–C3 hold (where $\mathcal{I}_{joint}$ is replaced with $\mathcal{I}_B$), and further suppose that the following condition also holds:*

$$\sum_{k=1}^{q}\left\|\hat{\tilde{T}}_{k-1}(L_O,\bar{L}_{M,k-1})-\tilde{T}_{k-1}(L_O,\bar{L}_{M,k-1})\right\|_2\left\|\hat{\pi}_{R_{Lk}}(L_O,\bar{L}_{M,k-1})-\pi_{R_{Lk}}(L_O,\bar{L}_{M,k-1})\right\|_2+ \left\|\hat{\tilde{T}}_q(L_O,\bar{L}_{Mq})-\tilde{T}_q(L_O,\bar{L}_{Mq})\right\|_2 \|\hat{\pi}_A(L)\hat{\pi}_{R_A}(L)-\pi_A(L)\pi_{R_A}(L)\|_2=o_p(n^{-1/2}).$$

Then, $\sqrt{n}(\widehat{\Psi}^a_{\mathbf{TMLE},\mathcal{I}_\mathbf{B}}-\Psi^a_{\mathcal{I}_B})\rightsquigarrow N(0,\sigma^2),\quad \sigma^2=Var(\phi^1_{P_{\mathcal{I}_\mathbf{B}}})$.

TMLE-C

Theorem H.4 (Weak convergence of TMLE-C)**.** *Suppose that the conditions C1–C3 hold (where $\mathcal{I}_{joint}$ is replaced with $\mathcal{I}_C$), and further suppose that the following condition also holds:*

$$\left\|\hat{T}_1(L)-T_1(L)\right\|_2 \Big(\|\hat{\pi}_A(L)\hat{\pi}_{R_A}(L)-\pi_A(L)\pi_{R_A}(L)\|_2+\|\hat{\pi}_A(L)-\pi_A(L)\|_2\Big)+ \left\|\hat{\tilde{T}}_0(L_O)-\tilde{T}_0(L_O)\right\|_2 \left\|\hat{P}(R_L=1|L_O,R_A)-P(R_L=1|L_O,R_A)\right\|_2=o_p(n^{-1/2}).$$

Then, $\sqrt{n}(\widehat{\Psi}^a_{\mathbf{TMLE},\mathcal{I}_\mathbf{C}}-\Psi^a_{\mathcal{I}_C})\rightsquigarrow N(0,\sigma^2),\quad \sigma^2=Var(\phi^1_{P_{\mathcal{I}_\mathbf{C}}})$.

I. Proof of multiple robustness

I.1 Proof of double robustness for $\mathcal{I}_{joint}$ and $\mathcal{I}_A$ We prove double robustness of our proposed TMLE-Joint under $\mathcal{I}_{Joint}$. (The proof of the proposed estimator under $\mathcal{I}_A$ is analogous, and we omit it here.) In particular, we show that an estimator based on Equation (A.2) is doubly robust in the sense that it will be consistent as long as

(1) the models for $P(A{=}a|R{=}1,L)$ and $P(R{=}1|L_O)$ are correctly specified, or
(2) the models for $T_1{=}E(Y|A{=}a,L,R{=}1)$ and $T_0{=}E(T_1|R{=}1,L_O)$ are correctly specified.

We consider an estimator based on Equation (A.2). Suppose that the models for $P(A{=}a|R{=}1,L)$ and $P(R{=}1|L_O)$ and parameterized by α_A and $\alpha_R{:=}\alpha_{R,\mathcal{I}_A}$, respectively, and the estimates of these parameters are denoted by $\hat{\alpha}_A$ and $\hat{\alpha}_R$. Suppose that α_A^*, α_R^*, $\kappa^*{:=}(\kappa_0^*,\kappa_1^*)$ and $\epsilon^*{:=}(\epsilon_0^*,\epsilon_1^*)$ are probability limits of $\hat{\alpha}_A$, $\hat{\alpha}_R$, $\hat{\kappa}{:=}(\hat{\kappa}_0,\hat{\kappa}_1)$ and $\hat{\epsilon}{:=}(\hat{\epsilon}_0,\hat{\epsilon}_1)$, respectively. Furthermore, let T_1^* be the limiting value of estimates for T_1, and T_0^* the limiting value of estimates for T_0. Under Equation (A.2) it suffices to show that

$$E\left\{\frac{I(A{=}a,R{=}1)}{P(A{=}a|R{=}1,L;\alpha_A^*)P(R{=}1|L_O;\alpha_R^*)}(Y-T_1^*)+\frac{I(R{=}1)}{P(R{=}1|L_O;\alpha_R^*)}(T_1^*-T_0^*)+T_0^*-\Psi^a_{\mathcal{I}_{joint}}\right\}=0$$

under scenario **(1)** where $(\alpha_A^*,\alpha_R^*)=(\alpha_A,\alpha_R)$, and thus $P(A{=}a|R{=}1,L;\alpha_A^*)P(R{=}1|L_O;\alpha_R^*)=P(A{=}a|R{=}1,L)P(R{=}1|L_O)$ and $P(R{=}1|L_O;\alpha_R^*)=P(R{=}1|L_O)$, **or** under scenario **(2)** where $(\kappa^*,\epsilon^*)=(\kappa,0)$ and thus $(T_1^*,T_0^*)=(T_1,T_0)$.

Proof. Suppose first that only the models for $P(A{=}a|R{=}1,L)$ and $P(R{=}1|L_O)$ are correctly specified. Then,

$$\begin{aligned}
&E\left\{\frac{I(A{=}a,R{=}1)}{P(A{=}a|R{=}1,L;\alpha_A^*)P(R{=}1|L_O;\alpha_R^*)}(Y-T_1^*)+\frac{I(R{=}1)}{P(R{=}1|L_O;\alpha_R^*)}(T_1^*-T_0^*)+T_0^*\right\}-\Psi^a_{\mathcal{I}_{joint}}\\
&=E\left\{\frac{I(A{=}a,R{=}1)}{P(A{=}a|R{=}1,L)P(R{=}1|L_O)}(Y-T_1^*)+\frac{I(R{=}1)}{P(R{=}1|L_O)}(T_1^*-T_0^*)+T_0^*\right\}-\Psi^a_{\mathcal{I}_{joint}}\\
&=E\{E(Y|A{=}a,R{=}1,L)-T_1^*+T_1^*-T_0^*+T_0^*\}-\Psi^a_{\mathcal{I}_{joint}}\\
&=E\{E(Y|A{=}a,L)\}-\Psi^a_{\mathcal{I}_{joint}}=0
\end{aligned}$$

Next, suppose that only the models for $T_1{=}E(Y|A{=}a,L,R{=}1)$ and $T_0{=}E(T_1|R{=}1,L_O)$ are correctly specified. Then,

$$\begin{aligned}
&E\left\{\frac{I(A{=}a,R{=}1)}{P(A{=}a|R{=}1,L;\alpha_A^*)P(R{=}1|L_O;\alpha_R^*)}(Y-T_1)+\frac{I(R{=}1)}{P(R{=}1|L_O;\alpha_R^*)}(T_1-T_0)+T_0\right\}-\Psi^a_{\mathcal{I}_{joint}}\\
&=E\left\{\frac{I(A{=}a,R{=}1)}{P(A{=}a|R{=}1,L;\alpha_A^*)P(R{=}1|L_O;\alpha_R^*)}(E(Y|A{=}a,R{=}1,L)-T_1)+\right.\\
&\qquad\qquad\left.\frac{I(R{=}1)}{P(R{=}1|L_O;\alpha_R^*)}(T_1-T_0)+T_0\right\}-\Psi^a_{\mathcal{I}_{joint}}
\end{aligned}$$

$$=E\left\{\frac{I(R=1)}{P(R=1|L_O;\alpha_R^*)}(E(T_1|R=1,L_O)-T_0)+T_0\right\}-\Psi^a_{\mathcal{I}_{joint}}$$
$$=E(T_0)-\Psi^a_{\mathcal{I}_{joint}}=0$$

□

I.2 Proof of double robustness for $\mathcal{I}_B$ The estimator based on TMLE-B is consistent as long as:

(1) the models for $P(A=a|R=1,L)$, $P(R_A=1|L,\bar{R}_{Lq}=1_q)$ and $P(R_{Lk}=1|\bar{L}_{M,k-1},R_{L,k-1}=1_k,L_O)$, $\forall k=1,\ldots,q$ are correctly specified, or
(2) the models for $\tilde{T}_k(\bar{L}_{Mk},L_O)$, $\forall k=0,\ldots,q$ are correctly specified.

The proof of double robustness mimicks the proofs for $\mathcal{I}_{joint}$ and $\mathcal{I}_A$ and thus we omit most of it here. However, we note that in order to prove that TMLE-B is consistent when the exposure and treatment models are correctly specified at all time points, we make use of the following representation of the efficient influence function:

$$\begin{aligned}\phi^1_{P_{\mathcal{I}_\mathbf{B}}}=&\frac{I(A=a,R_A=1,\bar{R}_{Lq}=1_q)}{\pi_A(L)\pi_{R_A}(L)\prod_{j=1}^q\pi_{R_{Lj}}(\bar{L}_{M,j-1},L_O)}\{Y-\tilde{T}_q(L)\}+\\&\sum_{k=1}^q\frac{I(\bar{R}_{Lk}=1_k)}{\prod_{j=1}^k\pi_{R_{Lj}}(\bar{L}_{M,j-1},L_O)}\{\tilde{T}_k(\bar{L}_{Mk},L_O)-\tilde{T}_{k-1}(\bar{L}_{M,k-1},L_O)\}+\tilde{T}_0(L_O)-\Psi^a_{\mathcal{I}_B}\\=&\frac{I(A=a,R_A=1,\bar{R}_{Lq}=1_q)}{\pi_A(L)\pi_{R_A}(L)\prod_{j=1}^q\pi_{R_{Lj}}(\bar{L}_{M,j-1},L_O)}Y-\\&\frac{I(\bar{R}_{Lq}=1_q)}{\prod_{j=1}^q\pi_{R_{Lj}}(\bar{L}_{M,j-1},L_O)}\left\{\frac{I(A=a,R_A=1)}{\pi_A(L)\pi_{R_A}(L)}-1\right\}\tilde{T}_q(L)-\\&\sum_{k=1}^q\left[\frac{I(\bar{R}_{L,k-1}=1_{k-1})}{\prod_{j=1}^{k-1}\pi_{R_{Lj}}(\bar{L}_{M,j-1},L_O)}\left\{\frac{I(R_{Lk}=1)}{\pi_{R_{Lk}}(\bar{L}_{M,k-1},L_O)}-1\right\}\right]\tilde{T}_{k-1}(\bar{L}_{M,k-1},L_O)-\Psi^a_{\mathcal{I}_B}\end{aligned}$$

noting that by convention, we define $\prod_{j=1}^0(\cdot)=1$, for any $(\cdot)$ expression.

I.3 Proof of double robustness for $\mathcal{I}_C$ We prove double robustness of our proposed estimator under $\mathcal{I}_C$. In particular, we show that an estimator based on Equation (D.4) is doubly robust in the sense that it will be consistent as long as

(1) the models for $P(A=a|R=1,L)$, $P(R_A=1|L,R_L=1)$ and $P(R_L=1|L_O,R_A)$ are correctly specified, or
(2) the models for $T_1=E(Y|A=a,L,R=1)$ and $\tilde{T}_0=E(T_1|R_L=1,L_O,R_A)$ are correctly specified.

We consider an estimator based on Equation (D.4). Suppose that the models for $P(A=a|R=1,L)$ and $P(R_L=1|L_O,R_A)$ and parameterized by α_A, $\alpha_{RA}:=\alpha_{RA,\mathcal{I}_C}$ and $\alpha_{RL}:=\alpha_{RL,\mathcal{I}_C}$,

respectively, and the estimates of these parameters are denoted by $\hat{\alpha}_A$ and $\hat{\alpha}_{RL}$. Suppose that α_A^*, α_{RA}^*, α_{RL}^*, κ^* and ϵ^* are probability limits of α_A, α_{RA}, α_{RL}, κ and ϵ, respectively. Furthermore, let T_1^* be the limiting value of estimates for T_1, and $\widetilde{T}_0^*$ the limiting value of estimates for $\widetilde{T}_0$. Under Equation (D.4) suffices to show that

$$E\left[\frac{I(A{=}a,R_A{=}1,R_L{=}1)}{P(A{=}a|R{=}1,L;\alpha_A^*)P(R_A{=}1|L,R_L{=}1;\alpha_{RA}^*)}\left\{\sum_{r_A}\frac{P(R_A{=}r_A|L,R_L{=}1;\alpha_{RA}^*)}{P(R_L{=}1|L_O,R_A{=}r_A;\alpha_{RL}^*)}\right\}(Y-T_1^*)+\frac{I(R_L{=}1)}{P(R_L{=}1|L_O,R_A;\alpha_{RL}^*)}(T_1^*-\widetilde{T}_0^*)+\widetilde{T}_0^*-\Psi_{\mathcal{I}_C}^a\right]{=}0$$

under scenario **(1)** where $(\alpha_A^*,\alpha_{RA}^*,\alpha_{RL}^*){=}(\alpha_A,\alpha_{RA},\alpha_{RL})$, and thus $P(A{=}a|R{=}1,L;\alpha_A^*)P(R_A{=}1|L,R_L{=}1;\alpha_{RA}^*)P(R_L{=}1|L_O,R_A;\alpha_{RL}^*){=}P(A{=}a|R{=}1,L)P(R_A{=}1|L,R_L{=}1)P(R_L{=}1|L_O,R_A)$, **or** under scenario **(2)** where $(\kappa^*,\epsilon^*){=}(\kappa,0)$ and thus $(T_1^*,\widetilde{T}_0^*){=}(T_1,\widetilde{T}_0)$.

Proof. Suppose first that only the models for $P(A{=}a|R{=}1,L)$, $P(R_A{=}1|L,R_L{=}1)$ and $P(R_L{=}1|R_A,L_O)$ are correctly specified. Then,

$$E\left[\frac{I(A{=}a,R_A{=}1,R_L{=}1)}{P(A{=}a|R{=}1,L;\alpha_A^*)P(R_A{=}1|L,R_L{=}1;\alpha_{RA}^*)}\left\{\sum_{r_A}\frac{P(R_A{=}r_A|L,R_L{=}1;\alpha_{RA}^*)}{P(R_L{=}1|L_O,R_A{=}r_A;\alpha_{RL}^*)}\right\}(Y-T_1^*)+\frac{I(R_L{=}1)}{P(R_L{=}1|L_O,R_A;\alpha_{RL}^*)}(T_1^*-\widetilde{T}_0^*)+\widetilde{T}_0^*-\Psi_{\mathcal{I}_C}^a\right]$$

$$=E\left[\frac{I(A{=}a,R_A{=}1,R_L{=}1)}{P(A{=}a|R{=}1,L)P(R_A{=}1|L,R_L{=}1)}\left\{\sum_{r_A}\frac{P(R_A{=}r_A|L,R_L{=}1)}{P(R_L{=}1|L_O,R_A{=}r_A)}\right\}(Y-T_1^*)+\frac{I(R_L{=}1)}{P(R_L{=}1|L_O,R_A)}(T_1^*-\widetilde{T}_0^*)+\widetilde{T}_0^*-\Psi_{\mathcal{I}_C}^a\right]$$

$$=\sum_l\left\{\sum_{r_A}\frac{p(r_A|L{=}l,R_L{=}1)}{P(R_L{=}1|l_O,R_A{=}r_A;\alpha_{RL}^*)}\right\}(T_1-T_1^*)P(R_L{=}1,L{=}l)+E\left[\frac{I(R_L{=}1)}{P(R_L{=}1|L_O,R_A)}(T_1^*-\widetilde{T}_0^*)+\widetilde{T}_0^*-\Psi_{\mathcal{I}_C}^a\right]$$

$$=\sum_{r_A,l}(T_1-T_1^*)p(l_M|l_O,r_A,R_L{=}1)p(r_A,l_O)+\sum_{r_A,l}T_1^*p(l_M|l_O,r_A,R_L{=}1)p(r_A,l_O)+E(\widetilde{T}_0^*-\widetilde{T}_0^*)-\Psi_{\mathcal{I}_C}^a$$

$$=\sum_{r_A,l}T_1p(l_M|l_O,r_A,R_L{=}1)p(r_A,l_O)-\Psi_{\mathcal{I}_C}^a=0$$

Next, suppose that only the models for $T_1{=}E(Y|A{=}a,L,R{=}1)$ and $\tilde{T}_0{=}E(T_1|R{=}1,L_O,R_A)$ are correctly specified. Then,

$$E\left[\frac{I(A{=}a,R_A{=}1,R_L{=}1)}{P(A{=}a|R{=}1,L;\alpha_A^*)P(R_A{=}1|L,R_L{=}1;\alpha_{RA}^*)}\left\{\sum_{r_A}\frac{P(R_A{=}r_A|L,R_L{=}1;\alpha_{RA}^*)}{P(R_L{=}1|L_O,R_A{=}r_A;\alpha_{RL}^*)}\right\}(Y-T_1^*)+\right.$$

$$\left.\frac{I(R_L{=}1)}{P(R_L{=}1|L_O,R_A;\alpha^*_{RL})}(T_1^*-\widetilde{T}_0^*)+\widetilde{T}_0^*-\Psi^a_{\mathcal{I}_C}\right]$$

$$E\left[\frac{I(A{=}a,R_A{=}1,R_L{=}1)}{P(A{=}a|R{=}1,L;\alpha^*_A)P(R_A{=}1|L,R_L{=}1;\alpha^*_{RA})}\left\{\sum_{r_A}\frac{P(R_A{=}r_A|L,R_L{=}1;\alpha^*_{RA})}{P(R_L{=}1|L_O,R_A{=}r_A;\alpha^*_{RL})}\right\}(T_1-T_1)+\right.$$

$$\left.\frac{I(R_L{=}1)}{P(R_L{=}1|L_O,R_A;\alpha^*_{RL})}(T_1-\widetilde{T}_0)+\widetilde{T}_0-\Psi^a_{\mathcal{I}_C}\right]$$

$$=E\left\{\frac{I(R_L{=}1)}{P(R_L{=}1|L_O,R_A;\alpha^*_{RL})}(E(T_1|R_L{=}1,R_A,L)-\widetilde{T}_0)+\widetilde{T}_0\right\}-\Psi^a_{\mathcal{I}_C}$$

$$=E(\widetilde{T}_0)-\Psi^a_{\mathcal{I}_C}=0$$

□

I.4 Additional robustness against model misspecification Here, we show that our proposed estimator is actually robust against one more model misspecficiation scenario under $\mathcal{I}_{Joint}$. In particular, it is consistent and asymptotically normal as long as the model for T_1 and the model for $P(R{=}1|L_O)$ is correctly specified. This multiple robustness property was first noted in Molina *and others* (2017) and also described in Wen *and others* (2022).

Proof. Suppose that only the models for $T_1{=}E(Y|A,L,R{=}1)$ and $\pi_R{=}P(R{=}1|L_O)$ are correctly specified. Then,

$$E\left\{\frac{I(A{=}a,R{=}1)}{P(A{=}a|R{=}1,L;\alpha^*_A)P(R{=}1|L_O;\alpha^*_R)}(Y-T_1)+\frac{I(R{=}1)}{P(R{=}1|L_O;\alpha^*_R)}(T_1-T_0)+T_0\right\}-\Psi^a_{\mathcal{I}_{joint}}$$

$$=E\left\{\frac{I(A{=}a,R{=}1)}{P(A{=}a|R{=}1,L;\alpha^*_A)\pi_R}(Y-T_1)+\frac{I(R{=}1)}{\pi_R}(T_1-T_0^*)+T_0^*\right\}-\Psi^a_{\mathcal{I}_{joint}}$$

$$=E\left[\frac{R}{\pi_R}\left\{\frac{I(A{=}a)}{P(A{=}a|R{=}1,L;\alpha^*_A)}(Y-T_1)+T_1-T_0^*\right\}+T_0^*\right]-\Psi^a_{\mathcal{I}_{joint}}$$

$$=E\left[\frac{R}{\pi_R}\left\{\frac{\pi_A}{P(A{=}a|R{=}1,L;\alpha^*_A)}(T_1-T_1)+T_1-T_0^*\right\}+T_0^*\right]-\Psi^a_{\mathcal{I}_{joint}}$$

$$=E\left[\frac{\pi_R}{\pi_R}\left\{\underbrace{E(T_1|R{=}1,L_O)}_{=T_0}-T_0^*\right\}+T_0^*\right]-\Psi^a_{\mathcal{I}_{joint}}$$

$$=E(T_0)-\Psi^a_{\mathcal{I}_{joint}}=0$$

□

Analogously, we can show that our TMLE-A estimator proposed under $\mathcal{I}_A$ is also consistent and asymptotically normal under another scenario where the model for T_1 and for $P(R_L{=}1|L_O)$ are correctly specified. Note that TMLE-C estimator proposed under $\mathcal{I}_C$ does not enjoy the multiple robustness of TMLE-Joint and TMLE-A under $\mathcal{I}_{joint}$ and $\mathcal{I}_A$, respectively.

Next we show that TMLE-B is $q+2$ multiply robust. That is, TMLE-B is robust against $q+2$ model misspecification scenarios. That is, it is consistent for $\Psi^{a}_{\mathcal{I}_B}$ if (i) the models for $\pi_A(L)$, $P(R_A=1|L,\bar{R}_{Lq}=1_q)$ and $P(R_{Lk}=1|\bar{L}_{M,k-1},R_{L,k-1}=1_k,L_O)$, $\forall k=1,\ldots,q$ are correctly specified; (ii) the models for $\tilde{T}_k(\bar{L}_{Mk},L_O)$, $\forall k=0,\ldots,q$ are correctly specified; or (iii) for $k=1,\ldots,q$, $P(R_{Lj}=1|\bar{L}_{M,j-1},R_{L,j-1}=1_j,L_O)$ are correctly specified for $j=1,\ldots,k$ and $\tilde{T}_l(\bar{L}_{Mk},L_O)$ are correctly specified for $l=k,\ldots,q$. We give a sketch proof below, as it is analogous to the proof given in Wen *and others* (2022).

Suppose that the exposure and the first k models for missing $L_{M1},\ldots,L_{Mk}$ are correctly specified, and the models for $\tilde{T}_k,\ldots,\tilde{T}_q$ outcome models are correctly specified. For L_M a vector of length q, we work backwards from $q,\ldots,1$ and take the iterative conditional expectation of the first three terms in the efficient influence function evaluated at limiting values, i.e.

$$\frac{I(A=a,R_A=1,\bar{R}_{Lq}=1_q)}{\pi^*_A\pi^*_{R_A}\prod_{j=1}^{q}\pi^*_{R_{Lj}}}\{Y-\tilde{T}^*_q\}+\frac{I(\bar{R}_{Lq}=1_q)}{\prod_{j=1}^{q}\pi_{R_{Lj}}}\tilde{T}^*_q$$

first conditioning on R_L, $\bar{L}$ gives us:

$$E\left[\frac{I(\bar{R}_{Lq}=1_q)}{\prod_{j=1}^{q}\pi_{R_{Lj}}}\left\{\frac{I(A=a,R_A=1)}{\pi^*_A\pi^*_{R_A}}\left\{E(Y|L,\bar{R}_{Lq}=1_q)-\tilde{T}^*_q\right\}+T^*_q\right\}\right]$$
$$=E\left[\frac{I(\bar{R}_{Lq}=1_q)}{\prod_{j=1}^{q}\pi_{R_{Lj}}}\tilde{T}_q\right]$$

since $\tilde{T}^*_q=\tilde{T}_q$ if the outcome model for T_q is correctly specified, or if the exposure and R_A models are correctly specified. Following this logic and adding more terms from the efficient influence function (evaluated at limiting values) gives the desired result.

J. Complete case analysis

We define Missing Completely at Random as the following:

Assumption J.5 (MCAR). $R \perp\!\!\!\perp \{A,L,Y\}$.

Assumption J.6 (Positivity: MCAR). $P(R{=}1){>}0$.

Under MCAR, we can identify $E(Y^a)$ using the following:

$$\Psi^a_{MCAR} = E\{E(Y|A{=}a,L,R{=}1)|R{=}1\} \tag{J.17}$$

since the following two conditions hold under MCAR:

- $R \perp\!\!\!\perp Y|A,L$
- $R \perp\!\!\!\perp L$

Thus, under MCAR, a complete-case analysis based on the identifying formula (J.17) would be valid. An example of such an analysis is given by the following:

Algorithm 7 Algorithm for ICE under MCAR

Perform the following analysis on those whose $R{=}1$:

1: Fit a regression model $\eta(A,L;\kappa){=}g^{-1}([A,L]'\kappa)$ by regressing Y on A and L.
2: Predict $\hat{T}{:=}\hat{E}(Y|A{=}a,L,R{=}1;\hat{\kappa})$ (estimate of $T{:=}E(Y|A{=}a,L,R{=}1)$) from $\eta(A{=}a,L;\hat{\kappa})$.
3: Calculate the ICE estimator $\hat{\Psi}^a_{ICE-MCAR}{=}\mathbb{P}_{n_c}(\widehat{T})$, where n_c are the total number of individuals whose data are completely observed.

Note that MCAR is sufficient for deriving the identifying formula (J.17) for $E(Y^a)$, but not necessary. The two conditions given by $R \perp\!\!\!\perp Y|A,L$ and $R \perp\!\!\!\perp L$ are necessary, and these assumptions together imply $\mathcal{I}_{Joint}$.

K. Missing Exposure Only

Suppose that L include baseline covariates, A denotes binary treatment indicator taking values values a or a° (that may or may not be observed), Y denotes outcome and R_A denotes missing treatment indicator. For simplicity suppose that treatment is the only variable that contains missing data such that L and Y are fully observed. We observe $O{=}(L,R_A,R_AA,Y)$. Moreover, let Y^a denote the potential outcome variable if, possibly contrary to fact, the treatment had taken a value a for $a{\in}\{a,a^{\circ}\}$.

Our target estimand of interest is $E(Y^a)$ the marginal average potential outcome variable if, possibly contrary to fact, the treatment had taken a value a. Note that it is possible to estimate the average causal effect defined by $E(Y^{a=1}){-}E(Y^{a=0})$ for continuous outcomes under strong parametric assumptions (e.g., no effect modification of the causal effect) by complete case analysis and still yield consistent estimates of average causal effect (Hughes *and others*, 2019). However, otherwise complete case analysis is not valid (nor is the method valid for marginal potential causal mean outcomes defined by $E(Y^a)$).

K.1 Missingness mechanism assumptions associated with missing exposure or treatment variable In addition to Assumptions 1–2 of the main text, we consider the following missingness mechanisms:

- **M1.** $R_A{\perp\!\!\!\perp}A|Y,L$: Missingness of treatment is independent of treatment given outcome and covariates. (Treatment is missing at random)
- **M2.** $R_A{\perp\!\!\!\perp}Y|A,L$: Missingness of treatment is independent of outcome given treatment and covariates. (Treatment-outcome conditional independence)

Note that the first mechanism corresponds to the classic Missing At Random (MAR) assumption. We argue in the next section that our target estimand, $E(Y^a)$, is non-parametrically identified under Assumptions M1 & M2, along with respective positivity assumptions (see below), respectively.

We first note that $E(Y^a){=}E(Y|A{=}a^{\dagger})$ and is non-parametrically identified if $P(Y{=}y|A{=}a,L{=}l)$ is non-parametrically identified. Consider first the following decomposition of the joint probability distribution:

$$P(Y{=}y,A{=}a,L{=}l){=}\frac{P(R_A{=}1,Y{=}y,A{=}a,L{=}l)}{P(R_A{=}1|Y{=}y,A{=}a,L{=}l)}$$

This implies the following decomposition of the conditional probability:

$$P(Y{=}y|A{=}a,L{=}l){=}\frac{P(R_A{=}1,Y{=}y,A{=}a,L{=}l)}{P(R_A{=}1|Y{=}y,A{=}a,L{=}l)P(A{=}a,L{=}l)}.$$

Next, we consider this conditional distribution under assumptions M1–M2.

Assumption M1: MAR Under Assumption M1 and a positivity condition given by $P(R_A{=}1|Y,L){>}0$ for all $(l,y){\in}\text{supp}(L,Y)$, the conditional probability of $P(Y{=}y|A{=}a,L{=}l)$ is identifiable under the observed data:

$$\begin{aligned}
P(Y{=}y|A{=}a,L{=}l) &= \frac{P(R_A{=}1,Y{=}y,A{=}a,L{=}l)}{P(R_A{=}1|Y{=}y,A{=}a,L{=}l)P(A{=}a,L{=}l)} \\
&= \frac{P(R_A{=}1|Y{=}y,A{=}a,L{=}l)P(A{=}a|Y{=}y,L{=}l)P(Y{=}y|L{=}l)}{P(R_A{=}1|Y{=}y,A{=}a,L{=}l)P(A{=}a|L{=}l)} \\
&= \frac{P(A{=}a|Y{=}y,L{=}l)P(Y{=}y|L{=}l)}{P(A{=}a|L{=}l)} \\
&= \frac{P(A{=}a|Y{=}y,R_A{=}1,L{=}l)P(Y{=}y|L{=}l)}{P(A{=}a|L{=}l)} \quad \text{(since } R_A \perp\!\!\!\perp A|Y \text{ by M1)} \\
&= \frac{P(A{=}a|Y,R_A{=}1,L{=}l)P(Y{=}y|L{=}l)}{\sum_y P(A{=}a|Y{=}y,L{=}l)P(Y{=}y|L{=}l)} \\
&= \frac{P(A{=}a|Y,R_A{=}1,L{=}l)P(Y{=}y|L{=}l)}{\sum_y P(A{=}a|Y{=}y,R_A{=}1,L{=}l)P(Y{=}y|L{=}l)} \quad \text{(since } R_A \perp\!\!\!\perp A|Y \text{ by M1)}
\end{aligned}$$

Thus, $E(Y^a)$ is non-parametrically identified by the following formula:

$$\Psi^a_{M1} = \sum_l \sum_y y \frac{P(A{=}a|Y,R_A{=}1,L{=}l)P(Y{=}y|L{=}l)}{\sum_{y'} P(A{=}a|Y{=}y',R_A{=}1,L{=}l)P(Y{=}y'|L{=}l)} p(l)$$

This result was first shown in Kennedy (2020), who also derive the efficient influence function under a non-parametric model that places no restrictions of the observed data distribution.

Assumption M2: Treatment-outcome independence Under Assumption M2 and positivity conditions given by $P(R_A{=}1|L{=}l){>}0$ and $P(A{=}a|R_A{=}1,L{=}l){>}0$ for all $l{\in}\text{supp}(L)$, the conditional probability of $P(Y{=}y|A{=}a,L{=}l)$ is identifiable under the observed data:

$$P(Y{=}y|A{=}a,L{=}l) = P(Y{=}y|A{=}a,R_A{=}1,L{=}l) \quad \text{(since } R_A \perp\!\!\!\perp Y|A,L \text{ by M2)}$$

Thus, $E(Y^a)$ is non-parametrically identified by the following formula:

$$\begin{aligned}
\Psi^a_{M2} &= \sum_l \sum_y y P(Y{=}y|A{=}a,R_A{=}1,L{=}l)p(l) \\
&= E\{E(Y|A{=}a,R_A{=}1,L)\}
\end{aligned}$$

K.2 DAGs with Missing Treatment For simplicity, we assume $L{=}\emptyset$, but results are generalizable to include L. We will consider Directed Acyclic Graphs (DAGs) for Assumptions M1–M2 in turn. The topological ordering of R_A and Y has important implications, so we first consider the scenario in which R_A precedes Y, and then consider the case where R_A follows Y. In both cases we assume R_A follows A. We consider the most complex scenarios for each of the three assumptions. In addition, we let H_j, $j{\in}\mathbb{Z}$, denote a hidden (unmeasured) common cause of the aforementioned variables. Throughout, variables and arrows in

red denote causal relationships and variables (with causal relationships as indicated by their adjacent red arrows) that cannot exist by assumptions, respectively.

K.2.1 R_A *precedes* Y When R_A appears topologically after A but before Y, we consider the most complex scenario in Figure 7. By the Conditional treatment-outcome exchangeability (Assumption 2), there can be no unmeasured confounder H_3 that is a common cause A and Y.

[Figure 7 about here.]

DAG under Assumption M1:

In addition to the absence of unmeasured confounders H_3 of A and Y, M1 further requires that there be no arrow from $A \to R_A$, and there are no unmeasured common causes H_1 of R_A and A. Moreover, there can be no arrow from $R_A \to Y$ nor can there be unmeasured common causes H_2 between R_A and Y because both would cause Y act as a collider, opening the backdoor path $R_A \to Y \leftarrow A$. Figure 8 shows the reduced figure as a result of making Assumption M1.

[Figure 8 about here.]

DAG under Assumption M2:

In addition to the absence of unmeasured confounders H_3 of A and Y, M2 requires that there be no arrow from $R_A \to Y$, and there are no unmeasured common causes H_2 of R_A and Y. Figure 9 shows the reduced figure as a result of making Assumption M2. Interestingly, we see that the DAG in Figure 8 is nested within that of Figure 9. M2 can thus be viewed as a relaxation of the MAR assumption for treatment when R_A occurs before Y.

[Figure 9 about here.]

K.2.2 R_A *follows* Y When R_A appears topologically after A and Y, we consider the following most complex scenario in Figure 10. As before, assumption 3 implies there are no unmeasured common causes H_3 of A and Y.

[Figure 10 about here.]

DAG under Assumption M1:

In addition to the absence of unmeasured confounders H_3 of A and Y, there can be no arrow from $A \to R_A$, and there can be no unmeasured common causes H_1 of R_A and A. Moreover, there can be no unmeasured common causes H_2 of R_A and Y because this would cause Y to act as a collider, opening the backdoor path $R_A \leftarrow H_2 \to Y \leftarrow A$. Figure 11 shows the reduced figure as a result of making Assumption M1.

[Figure 11 about here.]

DAG under Assumption M2:

In addition to the absence of unmeasured confounders H_3 of A and Y, there can be no arrow from $Y \to R_A$, and there cann be no unmeasured common causes H_2 of R_A and Y. Figure 12 shows the reduced figure as a result of making Assumption M2. Interestingly, the reduced graphs from Figures 9 and 12 are identical regardless of topological ordering. However, unlike the case where R_A precedes Y, the DAG in Figure 8 is not nested in that of Figure 11; so M2 is not simply a relaxation of MAR in this case.

[Figure 12 about here.]

K.3 TMLE estimator under treatment-outcome conditional independence The efficient influence function under the treatment-outcome conditional independence assumption is given by:

$$\phi^1_{P_{M2}} = \frac{I(A=a, R_A=1)}{\pi_A \pi_R}(Y-T) + T - \Psi^a_{M2} \tag{K.18}$$

where we let $\pi_A = P(A=a|R_A=1, L)$, $\pi_R = P(R_A=1|L)$, and $T = E(Y|A=a, R_A=1, L)$. As such, we can construct TMLE estimators that will be guaranteed attain semiparametric efficiency bound when models for the nuisance functions are correctly specified. More over, the estimator will be doubly robust in the sense that it is consistent as long as the model for the outcome $T$is correctly specified, or if the models for the exposure and missingness mechanisms are correctly specified. The TMLE estimator is given by:

Algorithm 8 Algorithm for TMLE under treatment-outcome conditional independence

1: Obtain estimates $\hat{\pi}_A$ and $\hat{\pi}_R$ of π_A and π_R, respectively.
2: Among those with in those whose $R_A=1$, fit a regression model $\eta_1(A, L; \kappa) = g^{-1}([A, L]'\kappa)$ by regressing Y on A and L.[1]
3: Among those with $A=a$ and $R_A=1$, regress Y on an intercept with observational weight $(\hat{\pi}_A \hat{\pi}_R)^{-1}$ and an offset given by $g\{\eta(A, L; \hat{\kappa})\}$, i.e., solve for ϵ in

$$\mathbb{P}_n \left\{ \frac{I(A=a, R=1)}{\hat{\pi}_A \hat{\pi}_R} \left(Y - g^{-1}[g\{\eta(A, L; \hat{\kappa})\} + \epsilon] \right) \right\} = 0$$

4: Predict $\hat{T}$ (estimate of T) from $g^{-1}[g\{\eta(A=a, L; \hat{\kappa})\} + \hat{\epsilon}]$ for all observations.
5: Calculate the TMLE estimator $\widehat{\Psi}_{TMLE,M2} = \mathbb{P}_n(\hat{T})$.

[1]Alternatively, we can regress Y on L in those whose $A=a$ and $R_A=1$ through stratification on A.

L. Missing values in all variables

L.1 Simultaneous missingness Let R denote the observation indicator such that $R=1$ if L_M, A and Y are both observed and $R=0$ otherwise. The observed data are $O=(L_O,R,RL_M,RA,RY)$. Without further assumptions, $E(Y^a)$ is no longer identifiable under the standard causal assumptions above. In the following, we consider several further sets of assumptions and show that $E(Y^a)$ is identified under each.

We modify the missingness assumptions to accomodate missing values in all variables. First we consider a MAR assumption for both treatment and confounders as well as a positivity assumption for this missingness process:

*Assumption L.*7 (Modified MAR). $R \perp\!\!\!\perp \{A,L_M,Y\}|L_O$.

*Assumption L.*8 (Positivity: MAR). $P(R=1|L_O=l)>0, \ \forall(l_O)\in\text{supp}(L_O)$.

Note that the Modified MAR Assumption H.7 will now imply the $\mathcal{I}_{joint}$ Assumption under the new definition of R, and the identifying formula under $\mathcal{I}_{joint}$ will remain the same as the one in the main manuscript. Moreover, we can also utilize the TMLE-Joint estimator proposed for $\mathcal{I}_{joint}$ (again, using the new definition of R).

L.2 Separating observation indicators When we separate the observation indicators and let R_A, R_L, and R_Y denote the observation indicators for A, L_M and Y, respectively, $\mathcal{I}_A$ can be modified to the following ($\mathcal{I}_B$ can be modified in the same way):

*Assumption L.*9 (Modified $\mathcal{I}_A$). $(R_A,R_L,R_Y) \perp\!\!\!\perp Y|A,L$, and $R_L \perp\!\!\!\perp L_M|L_O$.

To identify $E(Y^a)$ from the observed data, we require the same positivity assumption (Positivity: $\mathcal{I}_A$) as the one in the main manuscript, the identifying formula under $\mathcal{I}_A$ will remain the same as the one in the main manuscript. The TMLE-A estimator under the modified $\mathcal{I}_A$ assumption is almost identical to the one in the main manuscript. A key difference is that we now are required to estimate $\pi_{R_Y}:=P(R_Y=1|R_A=R_L=1,L)$. We highlight the differences in steps 1–3 as follows (TMLE-B can be modified analogously):

1: Obtain estimates $\hat{\pi}_A$, $\hat{\pi}_{R_A}$, $\hat{\pi}_{R_L}$ and $\hat{\pi}_{R_Y}$ of π_A, π_{R_A}, π_{R_L} and π_{R_Y}, respectively.
2: Among those with $R_A=1$, $R_L=1$ and $R_Y=1$, fit a regression model $\eta_1(A,L;\kappa_1)=g^{-1}([A,L]'\kappa_1)$ by regressing Y on A and L, where g^{-1} denote a known inverse link function satisfying $\inf(Y)\le g^{-1}(u)\le\sup(Y)$ for all u.
3: Among those with $A=a$, $R_A=1$, $R_L=1$ and $R_Y=1$, regress Y on an intercept with observational weight $(\hat{\pi}_A\hat{\pi}_{R_A}\hat{\pi}_{R_L}\hat{\pi}_{R_Y})^{-1}$ and an offset given by $g\{\eta_1(A,L;\hat{\kappa}_1)\}$, i.e., solve for ϵ_1 in

$$\mathbb{P}_n\left\{\frac{I(A=a,R_A=1,R_L=1,R_Y=1)}{\hat{\pi}_A\hat{\pi}_{R_A}\hat{\pi}_{R_L}\hat{\pi}_{R_Y}}\left(Y-g^{-1}[g\{\eta_1(A,L;\hat{\kappa}_1)\}+\epsilon_1]\right)\right\}=0$$

4: ...

Steps 4 and onwards remain the same as the algorithm provided in the main manuscript.

Analogously, $\mathcal{I}_C$ can be modified to the following:

Assumption L.10 (modified $\mathcal{I}_C$)*.* $(R_A,R_L,R_Y)\perp\!\!\!\perp Y|A,L \quad \text{and} \quad R_L\perp\!\!\!\perp L_M|L_O,R_A$.

To identify $E(Y^a)$ from the observed data, we require the same positivity assumption (Positivity: $\mathcal{I}_C$) as the one in the main manuscript, and the identifying formula under $\mathcal{I}_C$ will remain the same as the one in the main manuscript. The modifications to the TMLE-B estimator under the modified $\mathcal{I}_C$ assumption is very similar to the one made in the TMLE-A estimator under the modified $\mathcal{I}_A$ assumption. Again, a key difference is that we now are required to estimate $\pi_{R_Y}:=P(R_Y=1|R_A=R_L=1,L)$, and an estimate of this will be included in the estimation procedure.

M. Simulation details and additional simulation studies

R codes that can be used to replicate the simulation study results are provided at `https://github.com/lw499/missing_data_causal_codes`. In all scenarios, we first generated unmeasured variables

$$U_1,U2,U3 \sim Ber(0.5).$$

We then generated covariates:

$$\begin{aligned} L_{M1} &\sim Ber(\text{expit}(1-4U_1+U_2)) \\ L_{M2} &\sim Ber(\text{expit}(1+3U_1-2U_2)) \\ L_O &\sim Ber(\text{expit}(U_1-U_3)). \end{aligned}$$

We then generated binary treatment A (as described below), observation indicator(s) (as described below) and a binary outcome as follows:

$$Y \sim Ber((\text{expit}(-1+A-L_{M1}-L_{M2}-2L_{M1}L_{M2}+L_O-2L_{M1}L_O)).$$

We generated treatment A and observation indicators differently in each of the four simulation scenarios:

I Jointly considering $\{R_A,R_L\}$ + MAR (Assumption 3):

$$\begin{aligned} A &\sim Ber(\text{expit}(-1+L_{M1}-2L_{M2}+2L_O)) \\ R &\sim Ber(\text{expit}(2-2L_O)), \end{aligned}$$

and $\{A,L_M\}$ was observed only when $R=1$.

II Considering R_A and joint of R_L + $\mathcal{I}_A$ (Assumption 5): We first generated $U_4 \sim N(0,1)$, which was an unmeasured common cause of $\{A,R_A\}$, $U_5 \sim N(0,1)$, an unmeasured common cause of $\{R_A,R_L\}$, and $U_6 \sim N(0,1)$, an unmeasured common cause of $\{A,R_L\}$. We allowed A and L_M to affect R_A:

$$\begin{aligned} A &\sim Ber(\text{expit}(-1+L_{M1}-2L_{M2}+L_O+3U_4+U_6)), \\ R_L &\sim Ber(\text{expit}(1+U_5-U_6+3L_O)), \\ R_A &\sim Ber(\text{expit}(0.5+2U_4-U_5+A+3L_O+L_{M1}-L_{M2}+R_L)). \end{aligned}$$

Here A was observed only when $R_A=1$, and L_M was observed when $R_L=1$.

III Separately considering $\{R_A,R_{L1},\ldots,R_{Lq}\}$ + $\mathcal{I}_B$ (Assumption 7): We first generated $U_4 \sim N(0,1)$, which was an unmeasured common cause of $\{A,R_A\}$, $U_5 \sim N(0,1)$, an unmeasured common cause of $\{R_A,R_L\}$, and $U_6 \sim N(0,1)$, an unmeasured common cause of $\{A,R_L\}$. We allowed A and L_M to affect R_A:

$$\begin{aligned} A &\sim Ber(\text{expit}(-1+L_{M1}-2L_{M2}+L_O+3U_4+U_6)), \\ R_{L1} &\sim Ber(\text{expit}(1+U_5-U_6+3L_O)), \end{aligned}$$

$$R_{L2}{\sim}Ber(\text{expit}(2{+}U_5{-}U_6{+}3L_O{-}2L_{M1})),$$
$$R_A{\sim}Ber(\text{expit}(0.5{+}2U_4{-}U_5{+}A{+}3L_O{+}L_{M1}{-}L_{M2}{+}R_{L1}{-}R_{L2})).$$

Here A was observed only when $R_A{=}1$, L_{M1} was observed when $R_{L1}{=}1$ and L_{M2} was observed when $R_{L2}{=}1$.

In the simulation study, the true parametric models for the outcome processes were the ones used for the simulation because we used a data-generating mechanism without unmeasured common causes of covariates and outcomes. The true parametric models for the treatment and missingness processes were saturated models that cannot be misspecified. Additionally, our misspecified exposure model was given by $\text{logit}\{\pi_A(\alpha_A)\}{=}\alpha_{A0}{+}\alpha_{A1}L_O{+}\alpha_{A2}L_{M2}$, and our misspecified outcome model was given by $\text{logit}\{\hat{T}_1(L;\kappa)\}{=}\kappa_0{+}\kappa_1A{+}\kappa_2L_O{+}\kappa_3L_{M1}L_{M2}$ (same model misspecification for $\hat{\hat{T}}_q(L;\kappa)$).

M.1 Additional simulation study to show efficiency gain in TMLE-B We show that in scenarios where ordering of variables in L_M does not matter, efficiency can be optimized by ordering the variables in increasing amount of missingness. With slight abuse in notation, we let X_1 and X_2 denote the previously defined L_{M1} and L_{M2} variables in the data generating mechanism from the previous subsection (such that $L_M{=}\{X_1,X_2\}$). We define L_{M1} and L_{M2} only after ordering of the variables in L_M are established. Similarly, we let R_{X1} and R_{X2} denote the observation indicator for X_1 and X_2, respectively. Continuing the data generating mechanism from the previous subsection, we generate A, R_A, R_{X1} and R_{X2} as follows:

$$A{\sim}Ber(\text{expit}(-1{+}X_1{-}2X_2{+}L_O{+}3U_4{+}U_6)),$$
$$R_A{\sim}Ber(\text{expit}(0.5{+}2U_4{-}U_5{+}A{+}3L_O{+}X_1{-}X_2{+}R_{X1}{-}R_{X2})),$$
$$R_{X1}{\sim}Ber(\text{expit}(1{+}U_5{-}U_6{+}3L_O)),$$
$$R_{X2}{\sim}Ber(\text{expit}(2{-}U_5{+}U_6{-}3L_O)).$$

Here A was observed only when $R_A{=}1$, X_1 was observed when $R_{X1}{=}1$ and X_2 was observed when $R_{X2}{=}1$. The amount of missingness in X_1 is approximately 20%, and the amount of missingness in X_2 is approximately 40%. Since neither R_{X1} nor R_{X2} depends on any of the incompletely observed covariates, any ordering of X_1 and X_2 will satisfy assumption 7 (since $\{R_{L1},R_{L2}\}\perp\!\!\!\perp\{L_{M2},L_{M1}\}|L_O$ regardless of what ordering we choose). We compare standard error of ICE-B and TMLE-B in two scenarios when all models are correctly specified. In scenario (1), we let X_1 be L_{M1}, and in scenario (2), we let X_2 be L_{M1}. In scenario (1), we treat approximately 20% of L_{M1} (X_1) as missing and 60% of L_{M2} (X_2) as missing. Conversely, in scenario (2), we treat approximately 40% of L_{M1} (X_2) as missing and 60% of L_{M2} (X_1) as missing. Additional comparison with ICE-A and TMLE-A are also conducted.

The results are given in Table 1. Not surprisingly, ICE-B and TMLE-B in scenario (i) are slightly more efficient than ICE-B and TMLE-B in scenario (ii), as estimators in scenario (i) use more available data than scenario (ii). Moreover, the performance of ICE-A and TMLE-A is almost identical to ICE-B and TMLE-B from scenario (ii). We would likely see more efficiency gains if there were more covariates with differing levels of observed data.

[Table 1 about here.]

M.2 Additional simulation study under $\mathcal{I}_C$ Separate Missingness + $\mathcal{I}_C$ (Assumption C.3): We first generated $U_4{\sim}N(0{,}1)$, which was an unmeasured common cause of $\{A{,}R_A\}$. We then generated $U_6{\sim}N(0{,}1)$, an unmeasured common cause of A and R_L, and allowed R_A to affect R_L. Finally we allowed both A and L_M to affect R_A:

$$A{\sim}Ber(\text{expit}(-1{+}L_{M1}{-}2L_{M2}{+}3L_O{+}3U_4{+}2U_6)),$$
$$R_A{\sim}Ber(\text{expit}(1{+}2U_4{+}A{+}2L_O{+}L_{M1}{-}L_{M2}))$$
$$R_L{\sim}Ber(\text{expit}(1{+}R_A{+}3L_O{-}2U_6)).$$

Here A was observed only when $R_A{=}1$, and L_M was observed when $R_L{=}1$. The results from the additional simulation study are shown in Table 2. When all models are correctly specified, IPW, ICE and TMLE are nearly unbiased. As long as one set of nuisance models (outcome models or exposure/missingness models) are correctly specified, TMLE remains nearly unbiased, but ICE and IPW are not. The TMLE is at least as efficient as IPW when all of the models are correctly specified, which is not predicted by theory (see Remark 1).

[Table 2 about here.]

N. Additional results from data analysis

Here we show results from TMLE when nuisance functions are estimated using the Highly Adaptive Lasso (HAL; Hejazi *and others*, 2020).

[Table 3 about here.]

References

Chernozhukov, V., Chetverikov, D., Demirer, M., Duflo, E., Hansen, C., Newey, W. and Robins, J. (2018). Double/debiased machine learning for treatment and structural parameters.

Hejazi, Nima S, Coyle, Jeremy R and van der Laan, Mark J. (2020). hal9001: Scalable highly adaptive lasso regression inr. *Journal of Open Source Software* **5**(53), 2526.

Hughes, R.A., Sterne, J.A.C. and Tilling, K. (2016). Comparison of imputation variance estimators. *Statistical methods in medical research* **25**(6), 2541–2557.

Hughes, Rachael A, Heron, Jon, Sterne, Jonathan AC and Tilling, Kate. (2019). Accounting for missing data in statistical analyses: multiple imputation is not always the answer. *International journal of epidemiology* **48**(4), 1294–1304.

Kennedy, Edward H. (2020). Efficient nonparametric causal inference with missing exposure information. *The international journal of biostatistics* **16**(1).

Little, R.J. A. and Rubin, D.B. (2002). *Statistical Analysis with Missing Data*, 2 edition. Hoboken, NJ.

Molina, J., Rotnitzky, A., Sued, M. and Robins, J.M. (2017). Multiple robustness in factorized likelihood models. *Biometrika* **104**, 561–581.

Paik, M.C. (1997). The generalized estimating equation approach when data are not missing completely at random. *Journal of the American Statistical Association* **92**, 1320–1329.

Rubin, Donald B. (2004). *Multiple imputation for nonresponse in surveys*, Volume 81. John Wiley & Sons.

van der Laan, Mark J and Rose, Sherri. (2011). *Targeted learning: causal inference for observational and experimental data*, Volume 4. Springer.

Wang, Naisyin and Robins, James M. (1998). Large-sample theory for parametric multiple imputation procedures. *Biometrika* **85**(4), 935–948.

Wen, L., Hernán, M. A. and Robins, J. M. (2022). Multiply robust estimators of causal effects for survival outcomes. *Scandinavian Journal of Statistics* **49**(3), 1304–1328.

Wen, L., Muniz-Terrera, G. and S.R., Seaman. (2017). Methods for handling longitudinal outcome processes truncated by dropout and death. *Biostatistics*. Manuscript submitted for publication.

Wen, L., Young, J. G., Robins, J. M. and Hernán, M. A. (2021). Parametric g-formula implementations for causal survival analyses. *Biometrics*.

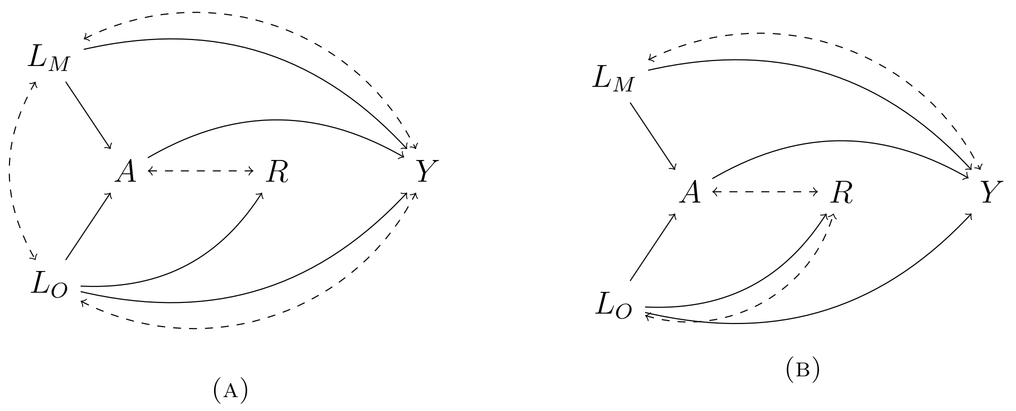

Figure 1. DAGs satisfying $\mathcal{I}_{joint}$ assumption A.1. Dashed lines represent unmeasured common causes.

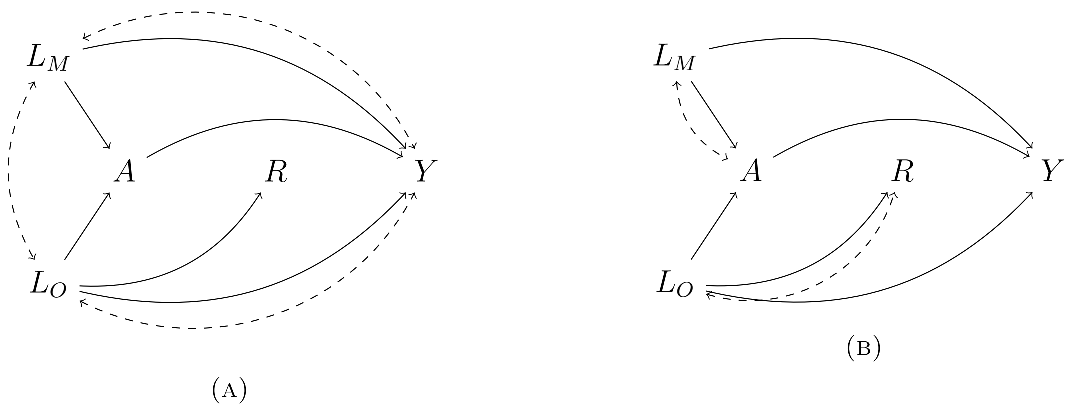

FIGURE 2. DAGs satisfying Assumption 3 (MAR) when R precedes Y. Dashed lines represent unmeasured common causes.

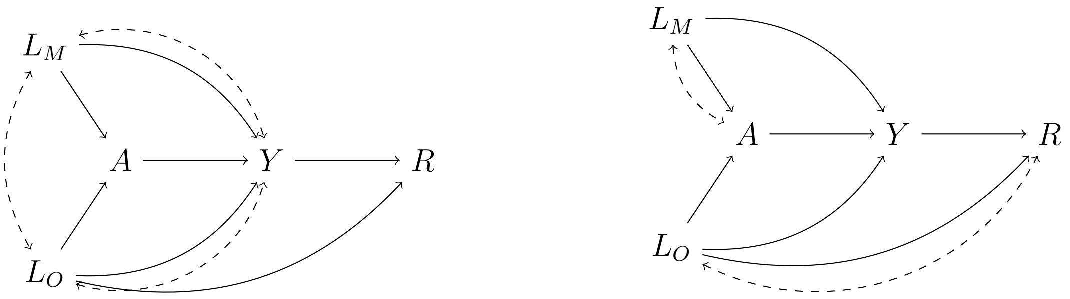

(A) (B)

FIGURE 3. DAGs satisfying Assumption 3 (MAR) when Y precedes R. Dashed lines represent unmeasured common causes.

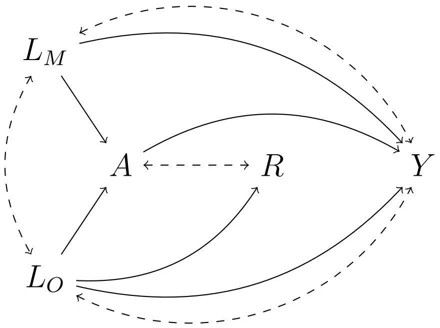

(A)

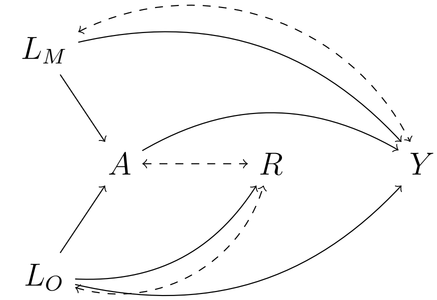

(B)

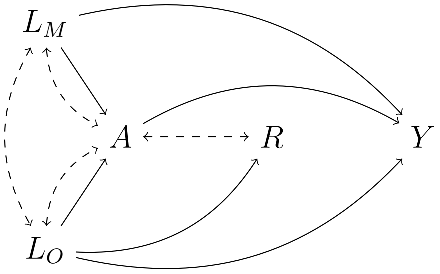

(C)

FIGURE 4. DAGs satisfying Assumption A.1 ($\mathcal{I}_{joint}$). Dashed lines represent unmeasured common causes.

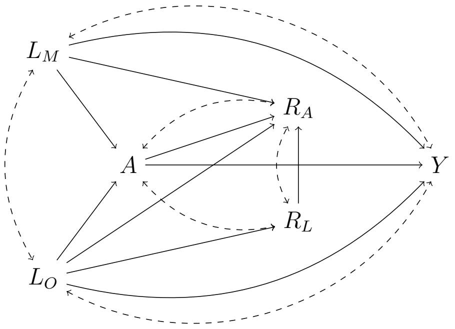

(A)

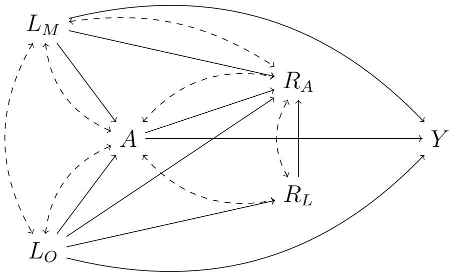

(B)

FIGURE 5. Examples of DAGs satisfying Assumption 5 ($\mathcal{I}_A$). Dashed lines represent unmeasured common causes.

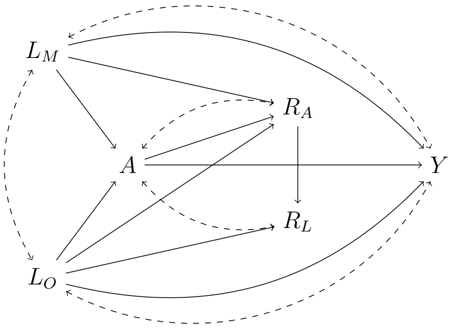

(A)

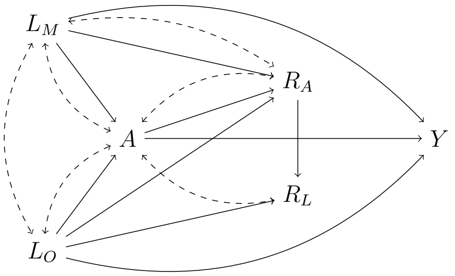

(B)

FIGURE 6. Examples of DAGs satisfying Assumption C.3. Dashed lines represent unmeasured common causes.

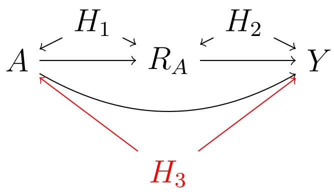

FIGURE 7. General complex scenario where the subgraph (A, R_A, Y) is complete, and hidden variables are added between all pairs of variables in (A, R_A, Y).

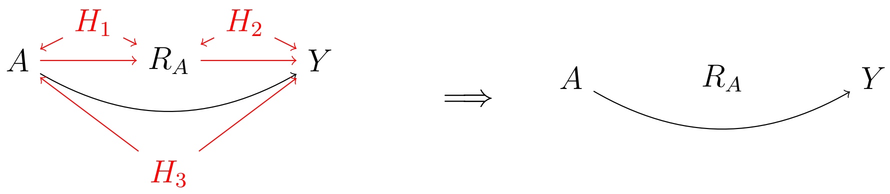

FIGURE 8. Missingness is independent of treatment given outcome (and covariates – omitted).

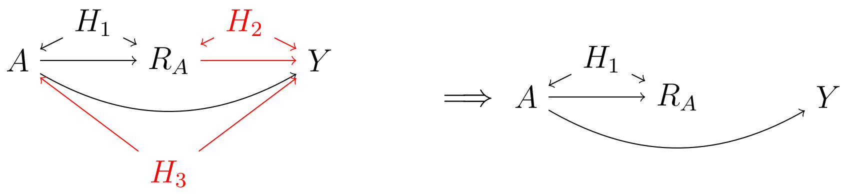

FIGURE 9. Missingness is independent of outcome given treatment (and covariates – omitted).

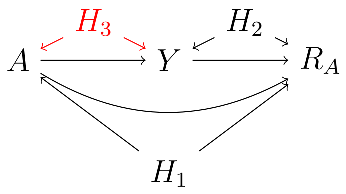

FIGURE 10. General complex scenario where the subgraph (A, Y, R_A) is complete, and hidden variables are added between all pairs of variables in (A, Y, R_A).

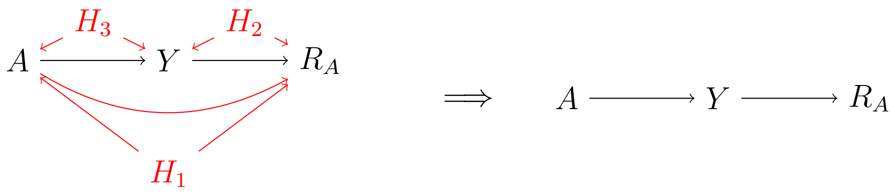

FIGURE 11. Missingness is independent of treatment given outcome (and covariates – omitted).

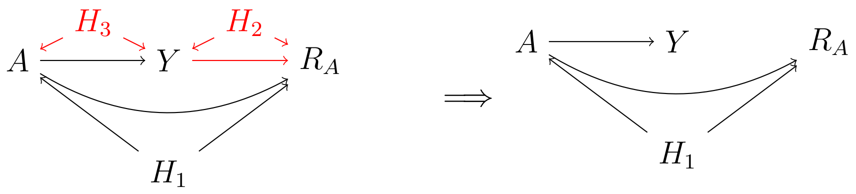

FIGURE 12. Missingness is independent of outcome given treatment (and covariates – omitted).

Ordering	(i) X_1 then X_2			(ii) X_2 then X_1		
**IV**	Bias	SE	Bias_s	Bias	SE	Bias_s
ICE-B	0.09	2.43	3.54	0.08	2.48	3.17
TMLE-B	0.09	2.53	3.57	0.08	2.57	3.18

**IV**	Bias	SE	Bias_s
ICE-A	0.08	2.48	3.18
TMLE-A	0.08	2.58	3.23

TABLE 1. Results for efficiency comparison of TMLE-B for n=2500 in simulation IV: Bias, standard error (SE), and standardized bias (Bias_s) all multiplied by 100. True value of $E(Y^{a=1})$=0.288.

	(i) Correctly Specified			(ii) Misspecified outcome model			(iii) Misspecified exposure model		
**V** ($\mathcal{I}_C$)	Bias	SE	Bias$_s$	Bias	SE	Bias$_s$	Bias	SE	Bias$_s$
CC	1.33	1.39	95.24	1.27	1.39	91.28	1.33	1.39	95.23
MI	-2.09	1.13	-184.95	-2.09	1.14	-184.47	-2.09	1.13	-184.94
ICE	0.01	1.42	0.64	-0.92	1.39	-66.23	0.01	1.42	0.64
IPW	0.01	1.49	0.38	0.01	1.49	0.38	-1.78	1.44	-124.00
TMLE	0.01	1.49	0.38	0.01	1.49	0.38	0.01	1.47	0.82

TABLE 2. Results for simulation V for n=2500: Bias, standard error (SE), and standardized bias (Bias$_s$) all multiplied by 100. True value of $E(Y^{a=1})$=0.288.

	Main analysis	
	$\text{TMLE}_{\mathcal{I}_\mathbf{A}}$	$\text{TMLE}_{\mathcal{I}_\mathbf{B}}$
Estimate	0.65%	0.59%
95% CI	(−0.10, 1.40)	(−0.16, 1.34)
	**Sensitivity analysis**	
	$\text{TMLE}_{\mathcal{I}_\mathbf{A}}$	$\text{TMLE}_{\mathcal{I}_\mathbf{B}}$
Estimate	1.02%	0.98%
95% CI	(0.18, 1.85)	(0.15, 1.81)

TABLE 3. Results for data analysis using the NHANES study (three cycles from 1999—2004). $\text{TMLE}_{\mathcal{I}_\mathbf{A}}$ and $\text{TMLE}_{\mathcal{I}_\mathbf{B}}$ denote TMLE estimators for the identifying formulae under $\mathcal{I}_A$ and $\mathcal{I}_B$, respectively. All results are multiplied by 100 (in %).
